# Publisher Correction: Relaxed selection underlies genome erosion in socially parasitic ant species

**DOI:** 10.1038/s41467-021-23950-y

**Published:** 2021-06-15

**Authors:** Lukas Schrader, Hailin Pan, Martin Bollazzi, Morten Schiøtt, Fredrick J. Larabee, Xupeng Bi, Yuan Deng, Guojie Zhang, Jacobus J. Boomsma, Christian Rabeling

**Affiliations:** 1 Centre for Social Evolution, Department of Biology, University of Copenhagen, Copenhagen, Denmark; 2 Institute for Evolution and Biodiversity, University of Münster, Münster, Germany; 3 BGI-Shenzhen, Shenzhen, China; 4 Entomología, Facultad de Agronomía, Universidad de la República, Montevideo, Uruguay; 5 Department of Entomology, National Museum of Natural History, Smithsonian Institution, Washington, DC, USA; 6 State Key Laboratory of Genetic Resources and Evolution, Kunming Institute of Zoology, Chinese Academy of Sciences, Kunming, China; 7 Department of Biology, University of Rochester, Rochester, NY USA; 8 School of Life Sciences, Arizona State University, Tempe, AZ USA

**Keywords:** Coevolution, Molecular evolution, Social evolution, Genome evolution

Correction to: *Nature Communications* 10.1038/s41467-021-23178-w, published online 18 May 2021.

The original version of this Article contained an error in Fig. 1, in which the species names are incorrectly displayed. The correct version of Fig. 1 is:


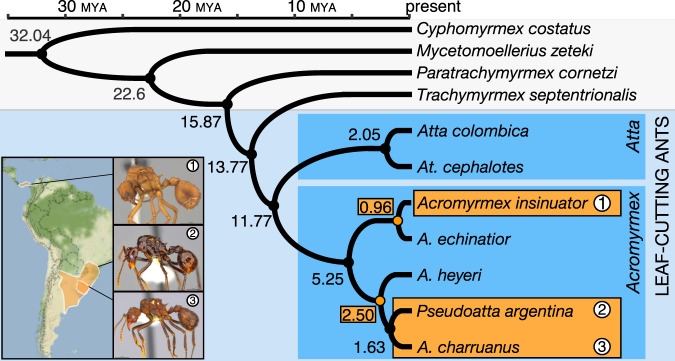


The error has been corrected in both the PDF and HTML versions of the Article.

