# Supplementary material for: Relaxed selection underlies genome erosion in socially parasitic ant species
Source: Nat Commun. 2021 May 18;12:2918. doi: 10.1038/s41467-021-23178-w (PMC8131649; doi:10.1038/s41467-021-23178-w)
Supplement: Supplementary file 1 — Supplementary Information [file 41467_2021_23178_MOESM1_ESM.pdf]

Supplementary Information: Relaxed selection underlies genome erosion in socially parasitic ant species

Lukas Schrader, Hailin Pan, Martin Bollazzi, Morten Schiøtt, Fredrick J. Larabee,
Xupeng Bi, Yuan Deng, Guojie Zhang, Jacobus J. Boomsma, Christian Rabeling
Email: Lukas.Schrader@wwu.de

1 Sample collection

Colonies of *Acromyrmex heyeri* and its social parasite *Acromyrmex charruanus* and *Pseudoatta argentina* were collected at Plantación Cruz Roja, a Eucalyptus tree plantation that is located six km southwest of Cerro Colorado in the Department of Florida in Uruguay (GPS coordinates: S33.9042°, W55.59418°, ± 600 m, elevation 224 m above sea level). Fieldwork was conducted during the southern hemisphere fall, between the 25th and the 27th of February 2013, and during the southern spring, between the 1st and the 5th of November 2013. *Acromyrmex insinuator* was collected from colonies of its host *Acromyrmex echinatior* in Gamboa, Panama in May 2014. For *A. insinuator*, we genotyped all individuals used for genome and transcriptome sequencing to ensure that they were indeed *A. insinuator* and not the morphologically very similar host *A. echinatior*.

2 DNA isolation

Whole ants were ground in 5 ml CTAB buffer (2% CTAB, 1.4 M NaCl, 0.1 M Tris-HCl, 20 mM EDTA, 1% PVP, 1% 2-mercaptoethanol, 0.2 mg/ml proteinase K [Roche Applied Science], 1 mg/ml RNAse A [Qiagen], pH 8.0) in a 10 ml disposable tube using a teflon pestle. The homogenate was incubated at 65°C for 3 hours and centrifuged at 7000 g for 5 min. The supernatant was transferred to a new tube and 5 ml phenol:CHCl3:IAA (25:24:1) pH 8 (Sigma Aldrich) was added and incubated for 5 min on a rotating wheel. The tubes were centrifuged for 30 min at 7000 g and 20°C, and the upper phase was transferred to a new tube. 5 ml CHCl3:IAA (24:1) was added to the tube and it was again incubated for 5 min on a rotating wheel. The tube was then centrifuged for 15 min at 7000 g and 20°C, and the upper phase was transferred to a new tube. The sample was mixed with 1/3 sample volume of 5 M NaCl and 2/3 sample volume of isopropanol, mixed briefly and then centrifuged for 30 min at 7000 g and 20°C. The supernatant was discarded and the pellet was washed with 75% ethanol and then air dried and

resuspended in 300 ul 10 mM Tris pH 8. DNA quantitation and quality analyses were performed using a NanoDrop ND-1000 instrument and agarose gel electrophoresis. Library preparation and sequencing was performed as described in Nygaard et al. (2016)[1].

3 RNA isolation

Total RNA was extracted using an RNeasy Plus Universal Mini Kit (Qiagen) from pools (between 10 to 30 individuals) of males for *A. insinuator*, of pupae, males, and queens, for *A. charruanus* and *P. argentina*, and of workers for *A. heyeri*. First tissues were homogenized in 900 µl Lysis Reagent in 2 ml screw cap tubes with a 5 mm steel bead using a Fastprep instrument at level 5.5 for 2 x 25 seconds. The sample was then centrifuged for 5 min at 20.000 g and the supernatant was transferred to a clean eppendorf tube and processed according to the protocol enclosed in the kit. RNA quantitation and quality analyses were performed using a NanoDrop ND-1000 instrument and agarose gel electrophoresis. We constructed RNA sequencing libraries for each species by first-strand cDNA synthesis with random hexamers and reverse transcription (Superscript II, Invitrogen). Second strand cDNA was synthesized with E. coli DNA PolI (Invitrogen). Double-stranded cDNA was purified with a Qiaquick PCR purification kit (QIAGEN) and sheared to 100 to 500 bp. After end repair, cDNA were ligated to 39 dA overhang and Illumina PE adapter oligo mix and size selected to 200 bp fragments by agarose gel. After PCR amplification, libraries were sequenced on an Illumina HiSeq 2000, generating 5 GB of paired-end data for each species.

4 Genome Assembly

de novo genome assemblies for the genomes of *A. charruanus*, *A. heyeri*, *A. insinuator*, and *P. argentina* were produced from Illumina sequencing data, using six different insert-size libraries (ranging from 200 bp to 10 kb) for each genome. After filtering raw read data, reads were corrected based on K-mer frequency. Initial genome assemblies were generated with SOAPdenovo based on corrected reads that passed quality control. Gap filling was done for each assembly using paired-end reads of short insert sizes. Details information are provided below. Supplementary Tables 1 to 4 provide information about the Illumina sequencing data generated per species.

4.1 Read filtering and correction

Reads meeting one of the following conditions were filtered:

- Reads with $> 10\%$ of Ns
- Reads with more than 40 low quality bases which are the bases with quality scores less than 3

- Reads with adapter contamination (match length >= 10 bp, mismatch <= 3)
- PCR duplicates
- The first 5 bases at the 5' end were trimmed for the reads of *A. insinuator* because of bad quality

To correct sequencing errors in raw reads, we used a k-mer frequency-based method to correct bases that were likely sequenced incorrectly. First, we indexed each read with 17-mer to build a library of 17-mer frequencies. Second, we corrected bases that were likely sequenced incorrectly, based on the concept that bases wrongly sequenced are likely within low frequency k-mers compared to other normal bases.

Supplementary Table 1: Sequencing data generated for *A. insinuator*.

Insert size (bp)	Total data (Gb)	Read length (bp)	Usable bases (Gb)
**200**	8.41936	100	7.57259
**500**	10.02576	100	8.97281
**800**	9.27466	100	7.6567
**2000**	4.04474	49	2.89631
**5000**	2.16241	49	1.6142
**10000**	2.26548	49	1.64559
**total**	36.19241		30.3582

Supplementary Table 2: Sequencing data generated for *A. charruanus*.

Insert size (bp)	Total data (Gb)	Read length (bp)	Usable bases (Gb)
**200**	9.00008	100	8.50639
**500**	10.604	100	9.11454
**800**	10.9858	100	8.50372
**2000**	4.08955	49	3.53523
**5000**	4.1161	49	3.0598
**10000**	4.96251	49	2.85055
**total**	43.758		35.5702

Supplementary Table 3: Sequencing data generated for *A. heyeri*.

Insert size (bp)	Total data (Gb)	Read length (bp)	Usable bases (Gb)
**200**	13.4779	100	12.8529
**500**	11.2218	100	9.82348
**800**	12.0886	100	9.37546
**2000**	5.18113	49	2.96348
**5000**	3.98544	49	2.87012
**10000**	4.65755	49	1.88709
**total**	50.6124		39.7725

Supplementary Table 4: Sequencing data generated for *P. argentina*.

Insert size (bp)	Total data (Gb)	Read length (bp)	Usable bases (Gb)
**200**	9.5821	100	9.11255
**500**	11.4759	100	9.5376
**800**	9.47501	100	6.95888
**2000**	4.24907	49	3.4878
**5000**	4.64131	49	2.97543
**10000**	3.61196	49	2.04852
**total**	43.0353		34.1208

4.2 Genome assembly with SOAPdenovo

We used `SOAPdenovo` to assemble the genomes. Based on previous experience that k-mer sizes can greatly affect the quality of the generated assemblies, we tested different k-mer sizes until we achieved

an optimal assembly for each genome, based on the N50 statistics of scaffolds and contigs. The best k-mer sizes we found are 49, 49, 43 and 49 for *A. charruanus*, *A. heyeri*, *A. insinuator*, and *P. argentina*, respectively. The versions of `SOAPdenovo` for *A. insinuator* was `Init_V1.06`, and `V2.04` for *A. charruanus*, *A. heyeri*, and *P. argentina*.

4.3 Gap filling

To achieve a more complete assembly, we further filled gaps within scaffolds using reads of short insert size (200 bp, 500 bp and 800 bp insert size) by `GapCloser`. Generally, `GapCloser` uses paired-end relationships and alignments to the raw assembly to close the gap within scaffold. It first aligns paired-end reads to assembly and identifies reads that located within a gap by paired-end relationship. For example, one read from a pair aligns to a non-gap region in a scaffold, while the other read from the same pair is inferred to locate within gap based on the insert size of this pair. Then, local assembling is performed using all reads located within a particular gap to close or shorten the gap. Summary statistics for the four genome assemblies are listed in Supplementary Tables 5 to 8.

Supplementary Table 5: Contig and scaffold summary statistics for the genome assembly of *A. insinuator*

	Contig		Scaffold	
	**Size (bp)**	**Number**	**Size (bp)**	**Number**
**N50**	32,678	2,639	1,146,926	69
**N60**	26,708	3,626	792,177	101
**N70**	20,590	4,868	628,676	143
**N80**	14,868	6,531	406,301	201
**N90**	8,945	9,014	238,380	296
**Longest**	268,529	-	7,732,671	-

Supplementary Table 6: Contig and scaffold summary statistics for the genome assembly of *A. charruanus*

	Contig		Scaffold	
	**Size (bp)**	**Number**	**Size (bp)**	**Number**
**N50**	2,901	27,074	1,858,278	38
**N60**	2,188	38,347	1,527,203	56
**N70**	1,559	53,680	1,067,240	79
**N80**	955	76,799	671,699	114
**N90**	374	123,145	310,675	182
**Longest**	35,030	-	11,773,965	-

Supplementary Table 7: Contig and scaffold summary statistics for the genome assembly of *A. heyeri*

	Contig		Scaffold	
	**Size (bp)**	**Number**	**Size (bp)**	**Number**
**N50**	1,254	62,568	1,503,884	56
**N60**	974	87,660	1,151,435	79
**N70**	715	120,731	679,510	114
**N80**	457	168,757	429,268	170
**N90**	219	256,199	170,970	277
**Longest**	16,852	-	7,573,979	-

Supplementary Table 8: Contig and scaffold summary statistics for the genome assembly of *P. argentina*

	Contig		Scaffold	
	**Size (bp)**	**Number**	**Size (bp)**	**Number**
**N50**	3,650	20,904	3,367,105	25
**N60**	2,860	29,032	2,775,721	34
**N70**	2,146	39,613	2,261,871	46
**N80**	1,457	54,353	1,400,759	62
**N90**	707	79,380	690,460	93
**Longest**	34,656	-	11,921,367	-

5 Repeat Annotation

We searched genomes for tandem repeats with Tandem Repeat Finder (`TRF v4.07b`). Transposable elements (TEs) were identified in the genome using a combination of homology-based and *de novo* approaches. The homology-based approach involves commonly used databases of known repeats.

- **Homology-based prediction**

  We used Repbase (release 21.01) annotations to annotate repeats based on homology. TEs in the genome assemblies were identified both at the DNA and protein level. `RepeatMasker` was applied for DNA-level identification. At the protein level, `RepeatProteinMask` was used to perform `Wu-BlastX 2.0` searches against the TE protein database.

- ***de novo*** **prediction**

  We used `LTR_FINDER` and `PILER` to build denovo repeat libraries based on the assembled ant genomes. `LTR_FINDER v1.06` searches the whole genome for typical LTR (long terminal repeat) elements. `PILER` (implemented in `PALS v1.0`) searches repeats in the genome by aligning the genome to itself. The results were used to construct a new library for `RepeatMasker v4.0.6`, which was run again to annotate *de novo* identified repeats in the genome. The combined repeat annotation results are listed in Supplementary Table 9. Supplementary Tables 10 to 20 provide detailed overviews for each of the eleven annotated genomes.

Supplementary Table 9: Repeat annotations of the assembled ant genomes.

**Species**	***A. insinuator***		***A. charruanus***		***A.heyeri***		***P. argentina***	
**Type**	**Sum (bp)**	**% genome**	**Sum (bp)**	**% genome**	**Sum (bp)**	**% genome**	**Sum (bp)**	**% genome**
**Trf**	7,741,906	2.62	7,201,795	2.4	5,901,854	1.96	5,251,993	1.86
**Repeatmasker**	39,725,953	13.47	32,388,958	10.8	28,226,547	9.38	32,655,204	11.58
**Proteinmask**	24,966,534	8.46	36,438,021	12.16	32,443,933	10.78	35,025,965	12.42
**De novo**	76,943,953	26.09	86,940,128	29	81,777,449	27.16	86,678,221	30.75
**Total**	86,831,390	29.44	93,183,398	31.08	87,876,708	29.19	91,806,971	32.57

6 Gene annotation

To generate comparable gene annotations, we (re-)annotated all eleven attine genomes analysed in this study using the same annotation pipeline. For annotating protein-coding genes of the four *de*

Supplementary Table 10: Summary of annotated repeats in the genome of *A. insinuator*.

	Repbase TEs		TE protiens		De novo		Total	
**Type**	**Length (bp)**	**% in genome**	**Length (bp)**	**% in genome**	**Length (bp)**	**% in genome**	**Length (bp)**	**% in genome**
**DNA**	24,193,663	8.2	6,527,298	2.21	43,550,025	14.77	48,892,297	16.58
**LINE**	4,687,969	1.59	1,876,380	0.64	5,256,969	1.78	8,088,168	2.74
**SINE**	14,370	0	0	0	0	0	14,370	0
**LTR**	5,676,456	1.92	4,767,959	1.62	8,138,129	2.76	12,408,041	4.21
**Other**	694	0	111	0	0	0	805	0
**Satellite**	0	0	0	0	24,428	0.01	24,428	0.01
**Simple_repeat**	5,271,643	1.79	10,237,596	3.47	8,923,041	3.03	10,958,633	3.72
**Low_complexity**	880,917	0.3	1,686,423	0.57	1,296,762	0.44	1,712,867	0.58
**Unknown**	0	0	0	0	14,322,306	4.86	14,322,306	4.86
**Total**	39,725,953	13.47	24,966,534	8.46	76,943,953	26.09	84,684,017	28.71

Supplementary Table 11: Summary of annotated repeats in the genome of *A. charruanus*.

	Repbase TEs		TE protiens		De novo		Total	
**Type**	**Length (bp)**	**% in genome**	**Length (bp)**	**% in genome**	**Length (bp)**	**% in genome**	**Length (bp)**	**% in genome**
**DNA**	8,219,306	2.74	8,626,654	2.88	21,592,412	7.2	23,871,469	7.96
**LINE**	1,479,969	0.49	4,330,474	1.44	6,722,738	2.24	8,312,617	2.77
**SINE**	19,573	0.01	0	0	293,855	0.1	310,041	0.1
**LTR**	3,617,260	1.21	4,108,859	1.37	6,870,530	2.29	8,682,975	2.9
**Other**	895	0	0	0	0	0	895	0
**Satellite**	34	0	74	0	601,225	0.2	601,265	0.2
**Simple_repeat**	3,293,477	1.1	1,659,429	0.55	5,186,994	1.73	5,750,848	1.92
**Low_complexity**	16,137,545	5.38	14,862,267	4.96	12,908,257	4.31	16,734,431	5.58
**Unknown**	48,436	0.02	0	0	37,173,983	12.4	37,221,367	12.42
**Total**	32,388,958	10.8	36,438,021	12.16	86,940,128	29	91,412,750	30.49

Supplementary Table 12: Summary of annotated repeats in the genome of *A. heyeri*.

	Repbase TEs		TE protiens		De novo		Total	
**Type**	**Length (bp)**	**% in genome**	**Length (bp)**	**% in genome**	**Length (bp)**	**% in genome**	**Length (bp)**	**% in genome**
**DNA**	6,987,577	2.32	7,699,626	2.56	15,392,952	5.11	17,869,681	5.94
**LINE**	1,381,910	0.46	4,134,406	1.37	7,560,982	2.51	9,712,339	3.23
**SINE**	17,475	0.01	0	0	354,198	0.12	368,385	0.12
**LTR**	3,517,248	1.17	4,048,826	1.34	6,040,546	2.01	8,047,966	2.67
**Other**	1,328	0	0	0	0	0	1,328	0
**Satellite**	310	0	64	0	104,581	0.03	104,645	0.03
**Simple_repeat**	2,283,919	0.76	1,395,407	0.46	3,399,427	1.13	3,863,855	1.28
**Low_complexity**	14,349,342	4.77	13,217,563	4.39	11,414,175	3.79	14,839,344	4.93
**Unknown**	31,748	0.01	0	0	40,992,838	13.62	41,022,406	13.63
**Total**	28,226,547	9.38	32,443,933	10.78	81,777,449	27.16	86,230,008	28.64

Supplementary Table 13: Summary of annotated repeats in the genome of *P. argentina*.

	Repbase TEs		TE protiens		De novo		Total	
**Type**	**Length (bp)**	**% in genome**	**Length (bp)**	**% in genome**	**Length (bp)**	**% in genome**	**Length (bp)**	**% in genome**
**DNA**	12,077,863	4.28	12,100,478	4.29	57,582,857	20.43	59,941,313	21.26
**LINE**	947,944	0.34	2,737,310	0.97	2,204,895	0.78	4,249,416	1.51
**SINE**	18,142	0.01	0	0	4,383	0	21,726	0.01
**LTR**	3,369,052	1.2	3,782,780	1.34	6,647,620	2.36	8,439,210	2.99
**Other**	526	0	0	0	0	0	526	0
**Satellite**	282	0	98	0	1,606	0	1,670	0
**Simple_repeat**	2,592,705	0.92	1,314,790	0.47	2,689,722	0.95	3,341,593	1.19
**Low_complexity**	13,979,127	4.96	12,880,144	4.57	9,947,277	3.53	14,438,840	5.12
**Unknown**	51,763	0.02	0	0	11,984,812	4.25	12,027,455	4.27
**Total**	32,655,204	11.58	35,025,965	12.42	86,678,221	30.75	90,621,451	32.15

novo sequenced ant species (*Acromyrmex charruanus*, *Acromyrmex heyeri*, *Acromyrmex insinuator* and *Pseudoatta argentina*) and seven previously published attine genomes (*Acromyrmex echinatior*, *Atta cephalotes*, *Atta colombica*, *Cyphomyrmex costatus*, *Paratrachymyrmex ("Trachymyrmex") cornetzi*, *Trachymyrmex septentrionalis*, and *Mycetomoellerius ("Trachymyrmex") zeteki*), we generated homology-based, *de novo* and transcriptome-based predictions and combined the results of three methods in `glean v1.0.1` to produce integrated gene sets for all species. Combined predictions from

Supplementary Table 14: Summary of annotated repeats in the genome of *A. echinatior*.

	Repbase TEs		TE protiens		De novo		Total	
**Type**	**Length (bp)**	**% in genome**	**Length (bp)**	**% in genome**	**Length (bp)**	**% in genome**	**Length (bp)**	**% in genome**
**DNA**	23,292,492	7.83	6,331,446	2.13	49,500,970	16.64	53,754,877	18.07
**LINE**	3,984,717	1.34	1,671,236	0.56	4,786,337	1.61	7,088,668	2.38
**SINE**	13,796	0	0	0	5,042	0	15,874	0.01
**LTR**	5,190,992	1.74	2,870,580	0.96	11,933,164	4.01	14,213,450	4.78
**Other**	816	0	0	0	0	0	816	0
**Satellite**	0	0	0	0	396,283	0.13	396,283	0.13
**Simple_repeat**	7,756,729	2.61	8,939,468	3	7,905,046	2.66	10,187,075	3.42
**Low_complexity**	1,535,441	0.52	1,695,875	0.57	1,238,671	0.42	1,729,644	0.58
**Unknown**	0	0	0	0	13,797,995	4.64	13,797,995	4.64
**Total**	41,314,251	13.89	21,433,784	7.2	83,274,046	27.99	88,297,631	29.68

Supplementary Table 15: Summary of annotated repeats in the genome of *At. cephalotes*.

	Repbase TEs		TE protiens		De novo		Total	
**Type**	**Length (bp)**	**% in genome**	**Length (bp)**	**% in genome**	**Length (bp)**	**% in genome**	**Length (bp)**	**% in genome**
**DNA**	21,492,575	6.77	7,390,269	2.33	37,740,786	11.88	42,338,607	13.33
**LINE**	2,866,370	0.9	444,919	0.14	1,067,990	0.34	3,275,120	1.03
**SINE**	10,667	0	0	0	0	0	10,667	0
**LTR**	2,894,048	0.91	1,568,645	0.49	3,347,031	1.05	5,983,410	1.88
**Other**	270	0	0	0	0	0	270	0
**Satellite**	0	0	0	0	43,815	0.01	43,815	0.01
**Simple_repeat**	9,962,950	3.14	11,053,168	3.48	10,041,897	3.16	11,857,380	3.73
**Low_complexity**	1,651,181	0.52	1,796,506	0.57	1,476,115	0.46	1,830,125	0.58
**Unknown**	0	0	0	0	14,512,882	4.57	14,512,882	4.57
**Total**	38,475,236	12.11	22,182,853	6.98	64,611,124	20.34	70,632,969	22.23

Supplementary Table 16: Summary of annotated repeats in the genome of *At. colombica*.

	Repbase TEs		TE protiens		De novo		Total	
**Type**	**Length (bp)**	**% in genome**	**Length (bp)**	**% in genome**	**Length (bp)**	**% in genome**	**Length (bp)**	**% in genome**
**DNA**	23,149,520	7.9	8,648,122	2.95	40,998,987	14	48,011,018	16.39
**LINE**	2,932,728	1	654,557	0.22	969,014	0.33	3,576,028	1.22
**SINE**	14,574	0	0	0	3,662	0	15,807	0.01
**LTR**	2,967,046	1.01	1,670,860	0.57	4,626,262	1.58	7,350,544	2.51
**Other**	544	0	0	0	0	0	544	0
**Satellite**	0	0	278	0	886,706	0.3	886,984	0.3
**Simple_repeat**	8,738,077	2.98	9,857,199	3.37	7,822,590	2.67	10,837,124	3.7
**Low_complexity**	1,659,936	0.57	1,828,077	0.62	1,255,606	0.43	1,858,991	0.63
**Unknown**	0	0	0	0	13,113,828	4.48	13,113,828	4.48
**Total**	38,994,700	13.31	22,586,372	7.71	65,690,769	22.43	75,005,981	25.61

Supplementary Table 17: Summary of annotated repeats in the genome of *C. costatus*.

	Repbase TEs		TE protiens		De novo		Total	
**Type**	**Length (bp)**	**% in genome**	**Length (bp)**	**% in genome**	**Length (bp)**	**% in genome**	**Length (bp)**	**% in genome**
**DNA**	18,562,378	5.74	5,339,353	1.65	39,842,433	12.33	46,499,844	14.39
**LINE**	3,166,319	0.98	2,031,451	0.63	7,358,328	2.28	9,147,886	2.83
**SINE**	19,910	0.01	0	0	38,025	0.01	56,601	0.02
**LTR**	6,007,369	1.86	5,089,479	1.58	20,080,494	6.21	21,351,531	6.61
**Other**	499	0	411	0	0	0	910	0
**Satellite**	0	0	0	0	10,334	0	10,334	0
**Simple_repeat**	7,822,893	2.42	9,178,095	2.84	7,536,109	2.33	9,870,411	3.05
**Low_complexity**	1,495,361	0.46	1,636,883	0.51	1,202,614	0.37	1,660,733	0.51
**Unknown**	0	0	0	0	25,872,838	8.01	25,872,838	8.01
**Total**	36,542,828	11.31	23,190,323	7.18	95,759,869	29.64	101,277,408	31.34

homology, *de novo* and transcriptome-based methods are summarized in Supplementary Table 21.

Whole protein sequences of 17 species (*A. echinatior*, *At. cephalotes*, *At. colombica*, *Camponotus floridanus*, *Ooceraea biroi*, *C. costatus*, *Dinoponera quadriceps*, *Harpegnathos saltator*, *Linepithema humile*, *Monomorium pharaonis*, *Pogonomyrmex barbatus*, *Solenopsis invicta*, *Pa. cornetzi*, *T. septentrionalis*, *M. zeteki*, *Vollenhovia emeryi*, and *Wasmannia auropunctata*; see Table 22) were used as references to perform homology-based gene predictions. The homology-based method consists of

Supplementary Table 18: Summary of annotated repeats in the genome of *Pa. cornetzi*.

	Repbase TEs		TE protiens		De novo		Total	
**Type**	**Length (bp)**	**% in genome**	**Length (bp)**	**% in genome**	**Length (bp)**	**% in genome**	**Length (bp)**	**% in genome**
**DNA**	35,901,669	8.93	10,535,270	2.62	78,045,625	19.41	85,243,800	21.2
**LINE**	6,735,485	1.68	4,091,204	1.02	15,254,094	3.79	17,881,903	4.45
**SINE**	19,124	0	0	0	54,608	0.01	73,405	0.02
**LTR**	7,498,557	1.87	5,936,998	1.48	20,554,025	5.11	22,414,034	5.58
**Other**	476	0	0	0	0	0	476	0
**Satellite**	0	0	0	0	742,366	0.18	742,366	0.18
**Simple_repeat**	8,127,556	2.02	9,745,686	2.42	7,141,027	1.78	10,419,442	2.59
**Low_complexity**	1,663,640	0.41	1,841,802	0.46	1,262,825	0.31	1,873,846	0.47
**Unknown**	0	0	0	0	39,400,815	9.8	39,400,815	9.8
**Total**	59,280,137	14.75	32,049,351	7.97	152,490,187	37.93	158,441,039	39.41

Supplementary Table 19: Summary of annotated repeats in the genome of *T. septentrionalis*.

	Repbase TEs		TE protiens		De novo		Total	
**Type**	**Length (bp)**	**% in genome**	**Length (bp)**	**% in genome**	**Length (bp)**	**% in genome**	**Length (bp)**	**% in genome**
**DNA**	20,255,909	6.86	4,522,232	1.53	30,844,615	10.44	35,881,456	12.15
**LINE**	3,917,799	1.33	1,366,833	0.46	5,098,146	1.73	6,944,047	2.35
**SINE**	13,191	0	0	0	30,344	0.01	39,781	0.01
**LTR**	3,668,915	1.24	2,067,595	0.7	6,586,922	2.23	8,296,454	2.81
**Other**	644	0	408	0	0	0	1,052	0
**Satellite**	0	0	0	0	0	0	0	0
**Simple_repeat**	7,762,807	2.63	8,833,334	2.99	7,335,893	2.48	8,969,592	3.04
**Low_complexity**	1,480,208	0.5	1,632,416	0.55	1,308,814	0.44	1,657,884	0.56
**Unknown**	0	0	0	0	22,193,401	7.51	22,193,401	7.51
**Total**	36,641,384	12.4	18,364,138	6.22	69,608,902	23.56	74,634,816	25.27

Supplementary Table 20: Summary of annotated repeats in the genome of *M. zeteki*.

	Repbase TEs		TE protiens		De novo		Total	
**Type**	**Length (bp)**	**% in genome**	**Length (bp)**	**% in genome**	**Length (bp)**	**% in genome**	**Length (bp)**	**% in genome**
**DNA**	14,626,379	5.43	3,214,557	1.19	28,695,497	10.65	33,400,037	12.4
**LINE**	1,754,417	0.65	1,119,264	0.42	2,721,752	1.01	4,028,499	1.5
**SINE**	14,365	0.01	0	0	6,821	0	18,526	0.01
**LTR**	2,616,403	0.97	2,111,071	0.78	5,848,451	2.17	7,100,199	2.64
**Other**	285	0	0	0	0	0	285	0
**Satellite**	0	0	0	0	617,774	0.23	617,774	0.23
**Simple_repeat**	6,111,343	2.27	7,084,573	2.63	6,686,588	2.48	8,348,218	3.1
**Low_complexity**	1,278,758	0.47	1,395,268	0.52	1,070,828	0.4	1,415,663	0.53
**Unknown**	0	0	0	0	22,308,082	8.28	22,308,082	8.28
**Total**	25,979,058	9.64	14,866,854	5.52	63,455,669	23.56	68,672,064	25.49

Supplementary Table 21: Summary statistics for protein-coding gene annotations for all eleven attine genomes used in this study.

**Species**	**Gene count**	**Avg. gene length (bp)**	**Avg. CDS length (bp)**	**Avg. exon number per gene**	**Avg. exon length (bp)**	**Avg. intron length (bp)**
A. insinuator	14599	9344.10	1468.37	6.04	243.10	1562.56
A. charruanus	14672	8849.33	1469.27	5.93	247.77	1497.01
A.heyeri	16144	7790.74	1346.29	5.43	247.73	1453.22
P. argentina	13259	9517.09	1478.59	5.76	256.72	1688.94
A. echinatior	15414	8529.12	1415.90	5.78	245.17	1489.64
At. cephalotes	14688	8775.13	1375.24	5.50	249.84	1642.76
At. colombica	15382	8566.23	1356.59	5.49	247.17	1606.25
C. costatus	15507	6836.18	1473.01	5.28	278.86	1252.43
Pa. cornetzi	15849	6875.00	1457.46	5.39	270.5	1234.63
T. septentrionalis	14432	9080.09	1476.64	5.84	253.04	1572.42
M. zeteki	14345	7962.11	1483.87	5.67	261.83	1387.99

four steps: 1) homology search across the whole genome to get a non-redundant collection of alignments using `tblastn`; 2) connect alignment blocks from the same protein sequence using `solar`; 3) use `genewise` (version 2.0) to generate gene structures based on the homology alignments. We used `augustus v2.5.5` with default parameters to produce *de novo* predictions, using homology-based annotations from *A. echinatior* as the training set. For transcriptome-based gene predictions, RNAseq

data were aligned to the genome of each corresponding species with `Tophat v2.1.0` (options: `-r 20 -mate-std-dev 10 -I 50000 -solexa1.3-quals`). Secondly, we used `Cufflinks v2.0.2` (with `-I 10000`) to predict gene structures according to the alignment results from `Tophat`.

Supplementary Table 22: GenBank accession IDs for seventeen ant species used for homology-based gene prediction.

Species	GenBank assembly accession
Acromyrmex echinatior	GCA_000204515.1
Atta cephalotes	GCA_000143395.2
Atta colombica	GCA_001594045.1
Camponotus floridanus	GCA_000147175.1
Ooceraea biroi	GCA_000611835.1
Cyphomyrmex costatus	GCA_001594065.1
Dinoponera quadriceps	GCA_001313825.1
Harpegnathos saltator	GCA_000147195.1
Linepithema humile	GCA_000217595.1
Monomorium pharaonis	GCA_000980195.3
Pogonomyrmex barbatus	GCA_000187915.1
Solenopsis invicta	GCA_000188075.1
Paratrachymyrmex "Trachymyrmex" cornetzi	GCA_001594075.1
Trachymyrmex septentrionalis	GCA_001594115.1
Mycetomoellerius "Trachymyrmex" zeteki	GCA_001594055.1
Vollenhovia emeryi	GCA_000949405.1
Wasmannia auropunctata	GCA_000956235.1

Cufflinks predicted 26419 transcripts in *P. argentina*, 30090 in *A. charruanus*, 32131 in *A. heyeri*, and 34082 in *A. insinuator* with similar distributions of FPKM values and transcript lengths (Figure 1).

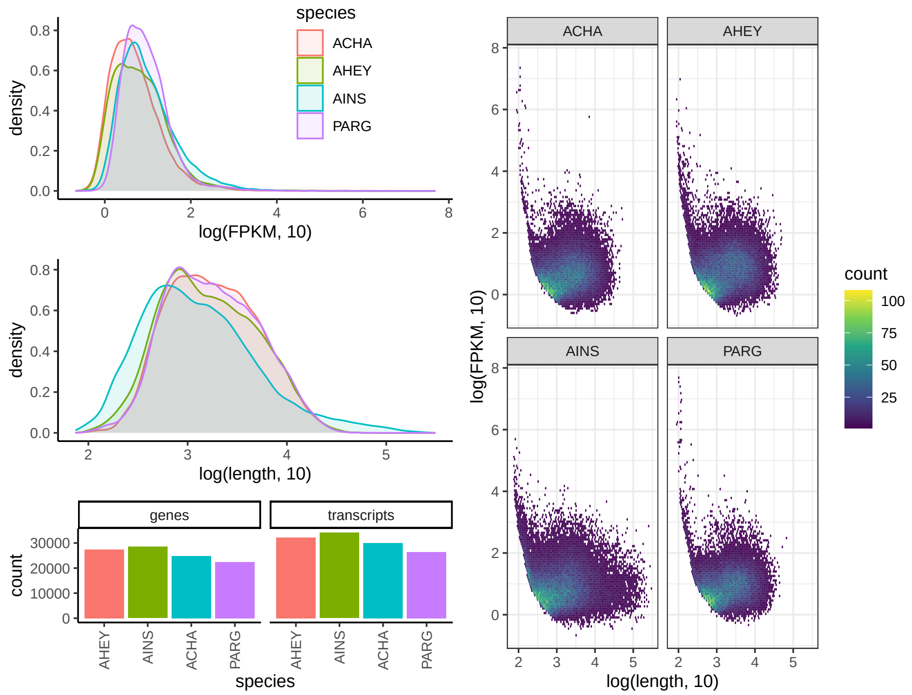

Supplementary Figure 1: Comparison of RNAseq data used for cufflinks transcriptome prediction.

7 Assembly filtering

Genome assemblies were filtered to remove bacterial and other non-insect scaffolds and contigs, creating version 2.1 of the assemblies. This was done by performing `blastn` (`-evalue 1e-10 -num_alignments 1`) runs of scaffolds and contigs against NCBI's nt database (retrieved June 2016). We then retrieved taxon identities for each hit and filtered out any scaffold and contig that did not align to a bilaterian taxon. This way, we identified fungal and bacterial contaminants in the assemblies. Furthermore, we identified mitochondrial sequences by blasting (`blastn`) against the mitochondrial genome of the ant *Wasmannia auropunctata* (GenBank: KX146469.1). Any scaffold/contig containing over 50% mitochondrial sequence was flagged as putative mitochondrial.

The filtering of the assemblies revealed the following contaminating and ant-specific scaffolds/contigs (Table 23), which were removed for subsequent analyses.

Supplementary Table 23: Contaminating and ant-specific sequences in the initial genome assemblies (v2.0)

	**Acha**	**Ahey**	**Ains**	**Parg**
**Ant scf/contig**	30758	51972	892	12930
**Mitochondrial scf/contig**	14	11	0	3
**Bacterial scf/contig**	0	0	0	0
**Fungal scf/contig**	0	11	18	28

8 Assembly and annotation quality

We used `quast v5.0.2` (`http://quast.bioinf.spbau.ru/`), to assess genome assembly qualities (summarized in Supplementary Table 24).To assess the quality of the gene annotation, we used `GAG v2.0.1` and `BUSCO v3` (using `hymenoptera_odb9` as background). The summary statistics for the gene annotations for v2.1 genomes are given in Supplementary Table 25, BUSCO-derived completeness estimates are provided in Supplementary Tables 26 (assembly completeness, BUSCO run in `genome` mode) and Supplementary Table 27 (annotation completeness, BUSCO run in `protein` mode). The analysis of the genome assembly completeness (BUSCO genome scores) showed that all genomes are above 90%, with *A. charruanus* having the lowest score. BUSCO scores of gene annotations range between 89.1% (Parg) to 96% (Acha).

```
#Genome annotation BUSCO scores (protein mode)
Acha  C:96.0% [S:95.6%,D:0.4%], F:2.7%, M:1.3%, n:4415
Ahey  C:92.5% [S:92.1%,D:0.4%], F:5.5%, M:2.0%, n:4415
Ains  C:95.5% [S:95.2%,D:0.3%], F:3.1%, M:1.4%, n:4415
Parg  C:89.1% [S:88.7%,D:0.4%], F:3.3%, M:7.6%, n:4415

#Genome annotation BUSCO scores (protein mode) for previously published attine genomes
Aech  C:95.9%[S:95.4%,D:0.5%],F:3.0%,M:1.1%,n:4415
Acep  C:91.6%[S:91.3%,D:0.3%],F:5.3%,M:3.1%,n:4415
Acol  C:95.5%[S:95.3%,D:0.2%],F:2.7%,M:1.8%,n:4415
Ccos  C:94.7%[S:94.0%,D:0.7%],F:3.0%,M:2.3%,n:4415
Pcor  C:95.4%[S:95.1%,D:0.3%],F:3.3%,M:1.3%,n:4415
Tsep  C:93.9%[S:93.6%,D:0.3%],F:3.6%,M:2.5%,n:4415
Mzet  C:94.0%[S:93.8%,D:0.2%],F:3.9%,M:2.1%,n:4415

#Genome assembly BUSCO scores (genome mode)
Acha  C:91.4% [S:91.1%,D:0.3%], F:3.3%, M:5.3%, n:4415
Ahey  C:94.9% [S:94.6%,D:0.3%], F:3.8%, M:1.3%, n:4415
Ains  C:97.3% [S:97.0%,D:0.3%], F:2.1%, M:0.6%, n:4415
Parg  C:97.1% [S:96.8%,D:0.3%], F:2.0%, M:0.9%, n:4415

#Genome assembly BUSCO scores (genome mode) for previously published attine genomes
Aech        C:97.0%[S:96.7%,D:0.3%],F:2.3%,M:0.7%,n:4415
Acep        C:94.6%[S:94.5%,D:0.1%],F:4.0%,M:1.4%,n:4415
Acol        C:97.8%[S:97.6%,D:0.2%],F:1.6%,M:0.6%,n:4415
Ccos        C:97.4%[S:96.7%,D:0.7%],F:1.9%,M:0.7%,n:4415
Pcor        C:97.4%[S:97.1%,D:0.3%],F:2.0%,M:0.6%,n:4415
Tsep        C:97.9%[S:97.6%,D:0.3%],F:1.5%,M:0.6%,n:4415
Mzet        C:97.4%[S:97.2%,D:0.2%],F:2.0%,M:0.6%,n:4415
```

Supplementary Table 24: Assembly statistics for v2.1 genomes of the inquiline project species. The columns *_brk give information about contig stats.

Assembly	Acha.v2.1	Acha_brk	Ahey.v2.1	Ahey_brk	Ains.v2.1	Ains_brk	Parg.v2.1	Parg_brk
# contigs (>= 0 bp)	30758	-	51972	-	892	-	12930	-
# contigs (>= 1000 bp)	1196	15129	1711	38826	762	13161	667	24491
# contigs (>= 5000 bp)	491	10835	729	17945	629	9920	255	15385
# contigs (>= 10000 bp)	426	8137	616	8474	604	7630	203	9379
# contigs (>= 25000 bp)	368	3855	495	1230	533	3914	174	2499
# contigs (>= 50000 bp)	311	1317	419	88	479	1438	163	342
Total length (>= 0 bp)	299772032	-	300797589	-	294656718	-	281522024	-
Total length (>= 1000 bp)	294481326	288376990	292026315	273251096	294571307	288212221	279368540	270756212
Total length (>= 5000 bp)	293120636	277181923	290369728	218363601	294294473	279590643	278499697	245684421
Total length (>= 10000 bp)	292670596	257338220	289587385	150741384	294119874	262752410	278152309	202022841
Total length (>= 25000 bp)	291733312	187323565	287608312	41984935	292934654	201854095	277729774	94144468
Total length (>= 50000 bp)	289538230	98557475	285041068	5274346	290903204	115211353	277350964	21750248
# contigs	2292	17875	3707	50949	855	15079	1104	28025
Largest contig	11725386	246377	7533355	115430	7732671	268529	11917447	155445
Total length	295248208	289917982	293440224	279225539	294641670	289267766	279678066	272586669
GC (%)	34.05	34.05	34.36	34.36	33.62	33.62	34.32	34.32
N50	1865358	35177	1543604	10905	1153669	39961	3483283	18070
N75	918883	18535	566575	5623	526957	21189	1744164	9714
L50	37	2426	53	7409	68	2099	24	4491
L75	91	5255	129	16258	165	4570	51	9625
# N's per 100 kbp	1801.02	4.43	4833.24	9.72	1817.87	3.24	2535.57	5.41

Supplementary Table 25: Annotation statistics for v2.1 genomes produced by `GAG v2.0.1`.

	Acha	Ahey	Ains	Parg
**Total sequence length**	299772032	300797589	294656718	281522024
**Number of genes**	14672	16139	14585	13249
**Number of mRNAs**	14672	16139	14585	13249
**Number of CDS**	14672	16139	14585	13249
**Overlapping genes**	1779	1887	1986	2165
**Contained genes**	864	949	928	1058
**Total gene length**	129837405	125763103	136380340	126102845
**Total CDS length**	21557150	21729798	21418088	19594779
**Shortest gene**	144	131	150	116
**Longest gene**	267585	253397	297908	294531
**mean gene length**	8849	7792	9351	9518
**% of genome covered by genes**	43.3	41.8	46.3	44.8

Supplementary Table 26: Assessing genome assembly completeness using `BUSCO v3` (`hymenoptera_odb9`) for v2.1 genomes of the inquiline project species.

	Acha	Ahey	Ains	Parg
**Complete BUSCOs (C)**	4035	4190	4294	4288
**Complete and single-copy BUSCOs (S)**	4022	4175	4281	4273
**Complete and duplicated BUSCOs (D)**	13	15	13	15
**Fragmented BUSCOs (F)**	145	167	94	87
**Missing BUSCOs (M)**	235	58	27	40
**Total BUSCO groups searched**	4415	4415	4415	4415

Supplementary Table 27: Assessing genome annotation completeness using BUSCO (`hymenoptera_odb9`) for v2.1 genomes of the inquiline project species.

	Acha	Ahey	Ains	Parg
**Complete BUSCOs (C)**	4238	4087	4217	3935
**Complete and single-copy BUSCOs (S)**	4221	4068	4202	3916
**Complete and duplicated BUSCOs (D)**	17	19	15	19
**Fragmented BUSCOs (F)**	118	242	136	144
**Missing BUSCOs (M)**	59	86	62	336
**Total BUSCO groups searched**	4415	4415	4415	4415

A comparison of BUSCO scores for the assembly (`genome` mode) and for the predicted gene set (`protein` mode) showed that our gene annotation pipeline in general recovers fewer BUSCOs than apparently contained in the assembly. The extent of this difference varies across species and is particularly strong in *P. argentina*, raising the question whether technical artefacts and/or biological differences could affect the perfomance of the annotation pipeline in the different species. Annotations in *P. argentina* are on average longer than in the other species, largely explained by an increase in intron size (see Supplementary Table 21). This could be the consequence of an increased tendency for annotating several consecutive genes into a single gene model (a known challenge when annotating ant genomes), resulting in a total decrease in gene count and an increase in average gene length. In general, the genome of *P. argentina* has diverged more strongly from the related species than the other species included in our annotation. This stronger divergence is apparent at the level of the genome structure and coding sequence (as we show in our study), which could very likely negatively affect the accuracy of our annotation pipeline. We did not find evidence for correlations between the perfomance of our gene annotation pipeline and technical differences (e.g. genome assembly quality (Table 24) or RNAseq data quality (Figure 1)). To minimize bias, we accounted for differences in annotation performance in the analysis of gene family size evolution by implementing an error correction (see below). We considered other analyses included in this study as not affected by differences in annotation com-

pleteness, as they are either based on genes shared across all species (e.g. phylogenetic inference, evolutionary rate analyes, synteny analysis), independent of gene annotations (analysis of genome rearrangements), or are based on independently produced gene annotation (analyses of specific gene families).

9 Single Copy Ortholog inference

We used `orthofinder v.2.2.6` to infer single copy orthologs across all available attine genomes. Supplementary Table 28 shows pairwise orthologs across all eleven species. Supplementary Table 29 shows an overall summary of the ortholog prediction.

Supplementary Table 28: 1-to-1 orthologs across all eleven attine genomes.

	Acep	Acha	Acol	Aech	Ahey	Ains	Ccos	Parg	Pcor	Tsep	Mzet
**Acep**	0	9781	11125	9823	9815	9849	9091	8903	9288	9507	9280
**Acha**	9781	0	10062	10762	11149	10639	9374	9748	9595	9953	9617
**Acol**	11125	10062	0	10041	9994	10022	9235	9112	9432	9708	9456
**Aech**	9823	10762	10041	0	10803	11217	9330	9422	9623	9957	9614
**Ahey**	9815	11149	9994	10803	0	10719	9288	9611	9651	9962	9575
**Ains**	9849	10639	10022	11217	10719	0	9300	9406	9511	9891	9604
**Ccos**	9091	9374	9235	9330	9288	9300	0	8562	9347	9171	9392
**Parg**	8903	9748	9112	9422	9611	9406	8562	0	8778	8981	8806
**Pcor**	9288	9595	9432	9623	9651	9511	9347	8778	0	9410	9432
**Tsep**	9507	9953	9708	9957	9962	9891	9171	8981	9410	0	9504
**Mzet**	9280	9617	9456	9614	9575	9604	9392	8806	9432	9504	0

Supplementary Table 29: Summary of Orthofinder results.

**Number of genes**	164262
**Number of genes in orthogroups**	156613
**Number of unassigned genes**	7649
**Percentage of genes in orthogroups**	95.3
**Percentage of unassigned genes**	4.7
**Number of orthogroups**	13656
**Number of species-specific orthogroups**	27
**Number of genes in species-specific orthogroups**	154
**Percentage of genes in species-specific orthogroups**	0.1
**Mean orthogroup size**	11.5
**Median orthogroup size**	11
**G50 (assigned genes)**	11
**G50 (all genes)**	11
**O50 (assigned genes)**	3755
**O50 (all genes)**	4103
**Number of orthogroups with all species present**	7425
**Number of single-copy orthogroups**	6338

Orthofinder identified 6338 single-copy orthologs across all eleven genomes (see Supplementary Table 29).

Orthofinder inferred 288 lineage specific genes in *A. charrauanus*, 267 in *P. argentina*, and 329 in *A. insinuator*. Analyses with `topGO` revealed no significantly enriched GO terms among these genes. Gene Ontology terms of the lineage-specific genes were generally associated with generic cellular functions (e.g. ”GO:0005515; protein binding” or ”GO:0055114; oxidation-reduction process”), with no initial evidence for changes associated with social parasitism. Supplementary Tables 30, 31, and 32 show all lineage-specific genes of the three parasites for which a homolog in swissprot could be retrieved ($e - value < 1e - 5$).

Supplementary Table 30: SwissProt homologs for lineage-specific genes in *P. argentina*

GeneID	SwissProt homolog	e-value	description
PARG06139	P10978	5.00E-11	POLX_TOBAC Retrovirus-related Pol polyprotein from transposon TNT 1-94
PARG00014	P13676	1.00E-14	ACPH_RAT Acylamino-acid-releasing enzyme
PARG04651	Q04164	2.00E-08	SAS_DROME Putative epidermal cell surface receptor
PARG05903	P33510	2.00E-08	NU5M_ANOQU NADH-ubiquinone oxidoreductase chain 5
PARG09385	Q9UBH6	0	XPR1_HUMAN Xenotropic and polytropic retrovirus receptor 1
PARG08558	Q7JQ07	7.00E-27	MOS1T_DROMA Mariner Mos1 transposase
PARG08686	P35072	3.00E-27	TCB1_CAEBR Transposable element Tcb1 transposase
PARG00023	O70511	1.00E-37	ANK3_RAT Ankyrin-3
PARG12497	Q31696	4.00E-39	NU5M_ANOQN NADH-ubiquinone oxidoreductase chain 5 (Fragment)
PARG00015	Q3UES3	9.00E-15	TNKS2_MOUSE Tankyrase-2
PARG00084	Q9VCU9	8.00E-139	DCR1_DROME Endoribonuclease Dcr-1
PARG06049	Q8BHG1	5.00E-48	NRDC_MOUSE Nardilysin
PARG12716	P19096	2.00E-13	FAS_MOUSE Fatty acid synthase
PARG07385	Q9VBP3	4.00E-20	TNKS_DROME Tankyrase
PARG00019	B2GV87	1.00E-08	PTPRE_RAT Receptor-type tyrosine-protein phosphatase epsilon
PARG06867	P19137	5.00E-06	LAMA1_MOUSE Laminin subunit alpha-1
PARG10278	Q76LL6	1.00E-21	FHOD3_MOUSE FH1/FH2 domain-containing protein 3
PARG00080	P91620	6.00E-82	SIF2_DROME Protein still life, isoforms C/SIF type 2
PARG05045	Q9LFG2	1.00E-15	DAPF_ARATH Diaminopimelate epimerase, chloroplastic
PARG00380	Q9VGZ1	0	CK5P1_DROME CDK5RAP1-like protein
PARG01468	O61365	1.00E-07	NACH_DROME Sodium channel protein Nach
PARG02164	P49632	9.00E-43	RL40_CAEEL Ubiquitin-60S ribosomal protein L40
PARG03379	Q9H0J4	1.00E-43	QRIC2_HUMAN Glutamine-rich protein 2
PARG03381	Q964T2	3.00E-39	CP9E2_BLAGE Cytochrome P450 9e2
PARG05094	Q9WUV0	7.00E-74	ORC5_MOUSE Origin recognition complex subunit 5
PARG07284	Q960E8	1.00E-13	TF2H1_DROME General transcription factor IIH subunit 1
PARG03997	Q7JQ07	1.00E-07	MOS1T_DROMA Mariner Mos1 transposase
PARG12300	Q8BZH4	3.00E-28	POGZ_MOUSE Pogo transposable element with ZNF domain
PARG02683	Q5RDP4	4.00E-21	SYLM_PONAB Probable leucine–tRNA ligase, mitochondrial
PARG08253	Q9VHD2	7.00E-36	MAAI2_DROME Probable maleylacetoacetate isomerase 2
PARG08319	Q7JQ07	5.00E-08	MOS1T_DROMA Mariner Mos1 transposase

Supplementary Table 31: SwissProt homologs for lineage-specific genes in *A. insinuator*

GeneID	SwissProt homolog	e-value	description
AINS02685	Q8NDN9	1.00E-14	RCBT1_HUMAN RCC1 and BTB domain-containing protein 1
AINS07972	Q6H236	6.00E-08	PEG3_BOVIN Paternally-expressed gene 3 protein
AINS07973	Q01133	4.00E-10	FMRA_CALPA Antho-RFamide neuropeptides
AINS06360	P49871	5.00E-97	ACT_MANSE Actin, muscle
AINS06013	Q53H47	5.00E-09	SETMR_HUMAN Histone-lysine N-methyltransferase SETMAR
AINS06041	Q6ZTA4	1.00E-101	TRI67_HUMAN Tripartite motif-containing protein 67
AINS00715	Q53H47	5.00E-16	SETMR_HUMAN Histone-lysine N-methyltransferase SETMAR
AINS11256	P47929	2.00E-10	LEG7_HUMAN Galectin-7
AINS12075	P35069	6.00E-71	H2B3_TIGCA Histone H2B.3
AINS11143	Q53H47	2.00E-06	SETMR_HUMAN Histone-lysine N-methyltransferase SETMAR
AINS02310	A0A1F4	1.00E-29	EYS_DROME Protein eyes shut
AINS00193	Q9Y5Q5	4.00E-31	CORIN_HUMAN Atrial natriuretic peptide-converting enzyme
AINS00194	P98073	6.00E-35	ENTK_HUMAN Enteropeptidase
AINS10960	Q9I8D0	1.00E-81	VPP1_CHICK V-type proton ATPase 116 kDa subunit a isoform 1
AINS03952	P53997	2.00E-44	SET_DROME Protein SET
AINS11980	Q8CDJ3	3.00E-13	BAKOR_MOUSE Beclin 1-associated autophagy-related key regulator
AINS02527	P30322	2.00E-162	DPOM_AGABT Probable DNA polymerase (Fragment)
AINS02896	Q96Q15	2.00E-150	SMG1_HUMAN Serine/threonine-protein kinase SMG1
AINS03031	Q9ULJ7	1.00E-24	ANR50_HUMAN Ankyrin repeat domain-containing protein 50
AINS09533	Q9VIQ9	6.00E-32	SICK_DROME Protein sickie
AINS09659	Q9JIT3	7.00E-31	TLE3_RAT Transducin-like enhancer protein 3
AINS09660	P16371	2.00E-20	GROU_DROME Protein groucho
AINS10855	Q6X0I2	1.00E-67	VGR_SOLIN Vitellogenin receptor
AINS11579	Q868Z9	6.00E-12	PPN_DROME Papilin
AINS04239	F1Q4S1	0	ATP9B_DANRE Probable phospholipid-transporting ATPase IIB
AINS01505	Q9VWE0	6.00E-08	DOME_DROME Cytokine receptor
AINS07336	P17971	8.00E-102	KCNAL_DROME Potassium voltage-gated channel protein Shal
AINS14172	Q3E811	3.00E-11	RRT15_YEAST Regulator of rDNA transcription protein 15
AINS13433	B2D0J4	1.00E-21	VDPP4_APIME Venom dipeptidyl peptidase 4
AINS00928	P47173	7.00E-26	YJ9J_YEAST Uncharacterized protein YJR142W
AINS07392	O15943	4.00E-14	CADN_DROME Neural-cadherin
AINS11216	Q3ZCU0	6.00E-10	YK006_HUMAN Putative uncharacterized protein FLJ37770
AINS00006	Q53H47	3.00E-38	SETMR_HUMAN Histone-lysine N-methyltransferase SETMAR
AINS04330	Q55EH8	3.00E-14	ABCGN_DICDI ABC transporter G family member 23
AINS04555	Q9ERM3	1.00E-16	DGAT1_RAT Diacylglycerol O-acyltransferase 1
AINS04623	P48809	1.00E-77	RB27C_DROME Heterogeneous nuclear ribonucleoprotein 27C
AINS12361	A8YPR6	2.00E-09	SVMI_ECHOC Snake venom metalloprotease inhibitor 02D01

Supplementary Table 32: SwissProt homologs for lineage-specific genes in *A. charruanus*

GeneID	SwissProt homolog	e-value	description
ACHA04622	Q53H47	3.00E-13	SETMR_HUMAN Histone-lysine N-methyltransferase SETMAR
ACHA03621	Q54XD0	2.00E-26	COQ3_DICDI Ubiquinone biosynthesis O-methyltransferase, mitochondrial
ACHA04268	Q7PWB1	2.00E-28	RETM_ANOGA Protein real-time
ACHA08007	Q31696	3.00E-10	NU5M_ANOQN NADH-ubiquinone oxidoreductase chain 5 (Fragment)
ACHA08316	Q53H47	7.00E-08	SETMR_HUMAN Histone-lysine N-methyltransferase SETMAR
ACHA05095	Q24174	2.00E-27	ABRU_DROME Protein abrupt
ACHA06930	Q3ZCU0	3.00E-17	YK006_HUMAN Putative uncharacterized protein FLJ37770
ACHA08168	Q34941	3.00E-11	COX1_LUMTE Cytochrome c oxidase subunit 1
ACHA10224	Q5IS68	2.00E-35	DCE1_PANTR Glutamate decarboxylase 1
ACHA02553	Q7G192	6.00E-10	ALDO2_ARATH Indole-3-acetaldehyde oxidase
ACHA03379	P07706	3.00E-28	NU5M_DROYA NADH-ubiquinone oxidoreductase chain 5
ACHA08353	P51899	4.00E-24	NU5M_ANOAR NADH-ubiquinone oxidoreductase chain 5 (Fragment)
ACHA03819	P18173	1.00E-11	DHGL_DROME Glucose dehydrogenase [FAD, quinone]
ACHA01971	P25003	2.00E-16	COX3_PISOC Cytochrome c oxidase subunit 3
ACHA08808	Q27746	7.00E-29	DNMT1_PARLI DNA (cytosine-5)-methyltransferase PliMCI
ACHA10869	Q9W1C9	4.00E-08	PEB3_DROME Ejaculatory bulb-specific protein 3
ACHA10886	Q4LDE5	6.00E-09	SVEP1_HUMAN Sushi, von Willebrand factor type A, EGF and pentraxin domain-containing protein 1
ACHA08746	B4NY70	2.00E-11	PESC_DROYA Pescadillo homolog
ACHA10436	Q3ZCU0	2.00E-15	YK006_HUMAN Putative uncharacterized protein FLJ37770
ACHA03410	B4NDL8	1.00E-18	NAAT1_DROWI Sodium-dependent nutrient amino acid transporter 1
ACHA14607	P43234	5.00E-25	CATO_HUMAN Cathepsin O
ACHA03848	P13607	3.00E-54	ATNA_DROME Sodium/potassium-transporting ATPase subunit alpha
ACHA04371	P17207	1.00E-08	SER3_DROME Serine protease 3
ACHA14651	Q03445	6.00E-36	GLR1_DROME Glutamate receptor 1
ACHA13238	Q9VW47	2.00E-79	MED12_DROME Mediator of RNA polymerase II transcription subunit 12
ACHA12815	Q36428	6.00E-19	NU5M_LOCMI NADH-ubiquinone oxidoreductase chain 5
ACHA04040	Q53H47	8.00E-10	SETMR_HUMAN Histone-lysine N-methyltransferase SETMAR
ACHA00059	O46108	6.00E-10	LIP3_DROME Lipase 3
ACHA00114	Q9BDT0	2.00E-16	GLCM_PANTR Glucosylceramidase
ACHA00134	Q9VLT5	1.00E-28	POE_DROME Protein purity of essence
ACHA00135	Q9VLT5	9.00E-08	POE_DROME Protein purity of essence
ACHA00138	Q8JH92	2.00E-15	PPD1A_XENLA Blood vessel epicardial substance-A
ACHA00151	P11247	5.00E-08	PERM_MOUSE Myeloperoxidase
ACHA07587	P23773	4.00E-31	GATA3_XENLA GATA-binding factor 3
ACHA00570	P98164	5.00E-10	LRP2_HUMAN Low-density lipoprotein receptor-related protein 2
ACHA13424	P47173	3.00E-12	YJ9J_YEAST Uncharacterized protein YJR142W
ACHA09310	Q9ERK4	9.00E-34	XPO2_MOUSE Exportin-2

10 Phylogeny

We used 4-fold degenerate sites of 1-to-1 single-copy orthologs (inferred with orthofinder) to compute a phylogenetic tree of attines.

We retrieved 1-to-1 single-copy orthologs across all eleven genomes (2 *Atta*, 4 *Acromyrmex*, 1 *Pseudoatta*, 1 *Trachymyrmex*, *Paratrachymyrmex*, 1 *Mycetomoellerius*, 1 *Cyphomyrmex*) and for each orthogroup aligned peptide sequences of the longest isoform of each gene using `prank v.150803`.

We used the following species cladistic tree as input for prank:

```
(CCOS,(MZET,(PCOR,(TSEP,((ACOL,ACEP),((AHEY,(ACHA,PARG)),(AECH,AINS)))))));
```

Amino-acid alignments were back-translated to CDS sequences with `pal2nal v.14`. Orthogroups that showed significant signs of recombination ($p < 0.01$ as tested with `Phi`) were removed. Subsequently, we extracted 4-fold degenerate sites from the remaining codon-alignments with a custom perl script (`4dSites.pl`). To avoid including alignments with saturated sites, we removed alignments of OGs where the correlation coefficient of the uncorrected genetic distance to the `TN93` genetic distance is smaller than the 1.5 x inter-quantile range for all OGs (< 0.6241, 1.5 IQR for all correlations).Supplementary Figure 2 shows that the data is not structured, suggesting all alignments come from a single population of unsaturated alignments. The final alignment contained 1,105,280 sites, with 355,593 variable and 160,416 parsimony informative sites. By running `jModelTest v.2.1.10` on

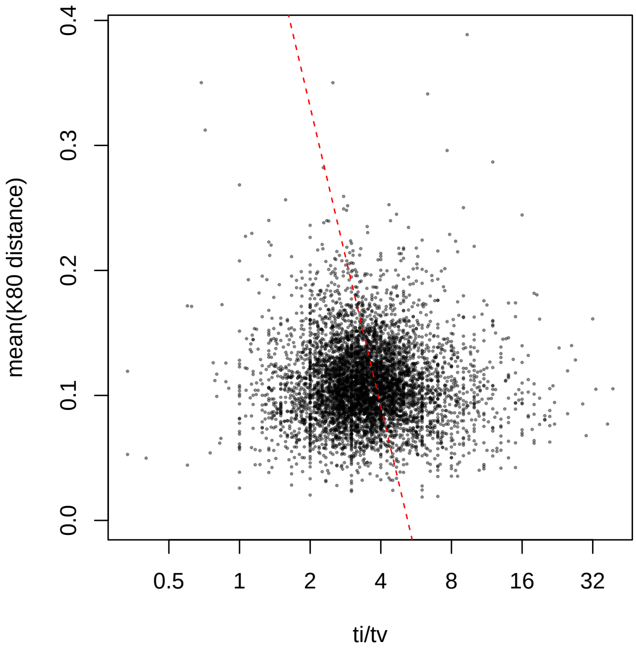

Supplementary Figure 2: The decadic logarithm of the transition/transversion ratios are shown on the x-axis and mean genetic distance (Kimura-80 substitution model) on the y-axis for each alignment. The red dotted line shows a fitted linear model (F-statistic: 8.89, p = 0.002886, corrected R-squared = 0.001339, 5879 degrees of freedom)

the alignment, we determined `GTR+I+G` as the best fitting nucleotide substitution model for the 4d-site

alignment.

We used PAUP* v.4a163 with bootstrap nreps=1000 search=heuristic/ addseq=random wap=tbr hold=1; to perform a Parsimony Bootstrap Analysis of all attines using the concatenated 4-fold degenerate site alignment as input. PAUP* was run in a heuristic search usign 1000 random-taxon-addition replicates, yielding a single optimal tree (Figure 3).

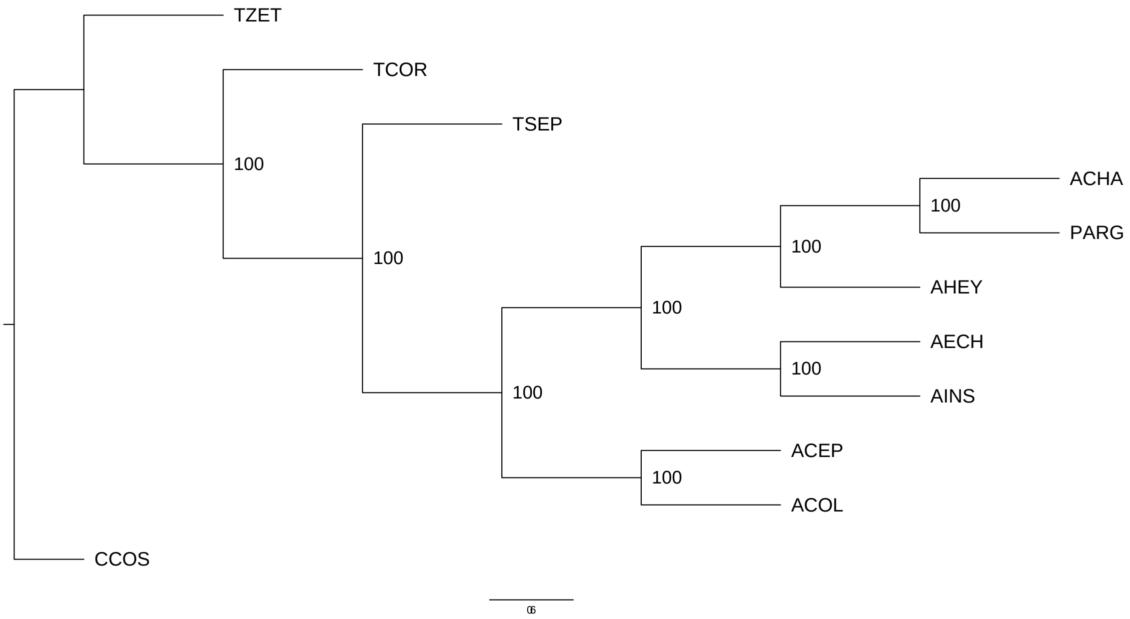

Supplementary Figure 3: Maximum parsimony tree for all attine genomes, using a concatenated alignment of 4-fold degenerate sites.

10.1 Phylogenetic tree inference using Maximum Likelihood and Bayesian Inference

Using the Maximum Parsimony-inferred tree as starting tree, we reconstruced the phylogeny of the eleven species under a Maximum Likelihood framework with RaxML v8.2.12 and under a Bayesian framework using MrBayes v3.2.6. We ran unpartitioned analyses on the concatenated alignment based on the assumption that 4-fold degenerate sites evolve similarly and largely under strict neutrality. For the Maximum Likelihood analyses runs with GTRCAT (-m GTRCAT) and GTRGAMMA (-m GTRGAMMA) nucleotide substitution models yielded very similar results. We finally settled for GTRCAT, set *Cyphomyrmex costatus* as outgroup, and performed 50 alternative Maximum Likelihood runs on distinct starting trees (-# 50). To infer support values for the best-scoring ML tree, we enabled random bootstrapping (-b 12345), using the pthreads version of RaxML and ran 500 regular bootstraps (-# 500).Supplementary Figure 4 shows the inferred species tree for all eleven species with corresponding bootstrap support in percent. Analysing the dataset using rapid bootstrapping (-x 12345 -# 500) and

GTRGAMMA substitution model produced very similar results.

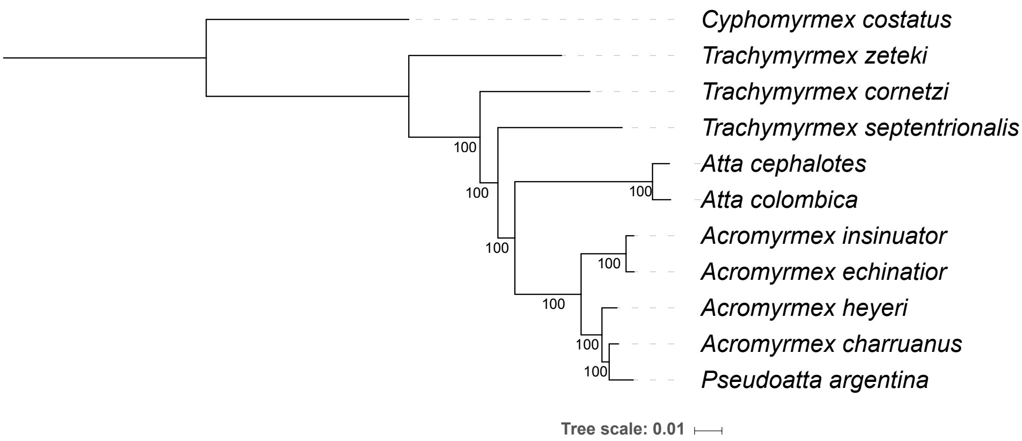

Supplementary Figure 4: Species tree for all available attine species, inferred under a Maximum Likelihood framework with RAxML, based on an unpartitioned alignment of 4-fold degenerate sites. Numbers below branches indicate bootstrap support for each node in percent (500 bootstrap replicates).

For a Bayesian analysis, MrBayes was run under a GTR model (`Nst=6`) with gamma distribution and a proportion of invariant sites (`lset nst=6 rates=invgamma`). We performed two independent iterations of MrBayes with two runs each (`nruns=2`) running for 200000 generations (`samplefreq = 10, 25% burn-in`). Both iterations produced similar results, suggesting convergence. All nodes showed a posterior probability of 1.00 and the structure of the tree matched the tree inferred from maximum parsimony (Fig. 3).Supplementary Figure 5 shows the species tree inferred with `MrBayes`.

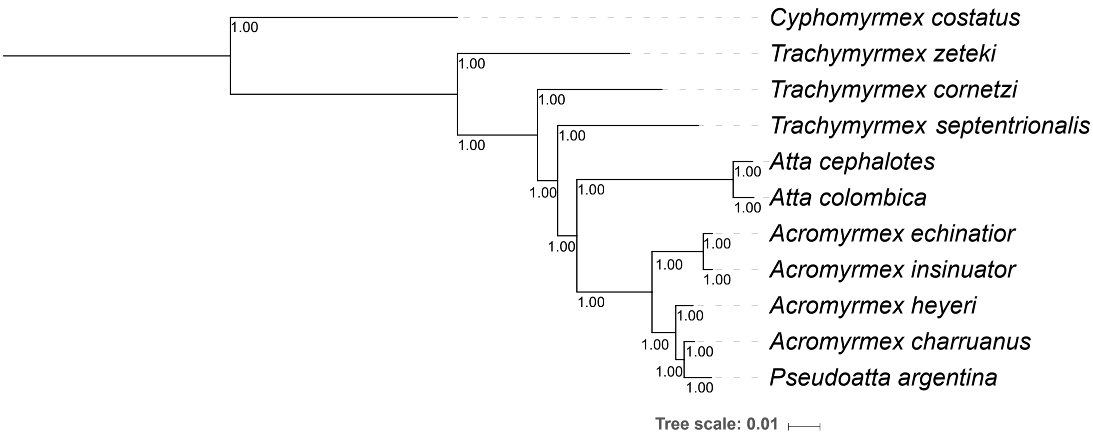

Supplementary Figure 5: Species tree for all available attine species, inferred under a Bayesian framework with MrBayes, based on an unpartitioned alignment of 4-fold degenerate sites. Numbers below branches indicate posterior probability support for each node.

11 Divergence estimates

We used `MCMCtree` (`PAML v4.9h`) to analyse the 4-fold degenerate site alignment for inferring divergence date estimates between the different attine species. `MCMCtree` uses a Bayesian approximate likelihood approach to infer divergence dates using a calibrated topological tree as input. We calibrated the tree using fossil evidence and estimates of genus splits published previously.

Using `baseml`, we estimated an overall substitution rate and alpha that will subsequently be used in `MCMCtree` runs. For generating overall estimates for substitution rates and alpha, we used a point calibrated phylogeny following Li et al 2018[2], which employ fossil calibration for the split between *M. zeteki* and *T. septentrionalis*, *Pa. cornetzi* and the leaf-cutting ants. The fossil referred by Li et al. (2018) is Dominican amber, which is estimated to have a median age of 17.5 million years[2]. This strict setting of a calibration point is used as a starting point for sampling from the prior. The point calibrated phylogeny used in `baseml` is:

```
(((PCOR,(TSEP,(((AHEY,(ACHA,PARG)),(AECH,AINS)),(ACEP,ACOL)))),MZET)'@0.175',CCOS);
```

The subsequent runs of mcmctree were performed using a phylogeny with two soft calibrations at different nodes of the phylogeny. The first calibration is set for the split between *T zeteki* and *T. septentrionalis*, *Pa. cornetzi* and the leaf-cutting ants, following the approach of Li et al. 2018[2]. Hence, this split is calibrated as a truncated Cauchy distribution by `'L(0.15, 0.1, 1, 0.1)'`, defining the split to be at least 15 Ma.

We used a `S2N` (skew 2 normals) distribution for the second calibration point at the split between the two leaf-cutting ant genera *Atta* and *Acromyrmex*. This calibration is based on previous estimates of the divergence dates between the two genera. Some studies suggested a split ca. 10 Ma (Nygaard et al 2011[3], Mikheyev et al 2010[4], Schultz & Brady 2008[5], Li et al 2018[2]), while others suggest an older origin of ca. 16 Ma (Nygaard et al 2016[1], Jesovnik et al 2016[6], Brandstetter et al 2017[7]).
In mcmctree a "skew 2 normal" prior distribution can be set using the following syntax in the input tree: `SN2(p1, loc1, scale1, shape1, loc2, scale2, shape2)`. We defined this distribution to capture older estimates with 16.2, 19.9, and 16.93 Ma from Nygaard et al 2016[1], Jesovnik et al 2016[6] and Brandstetter et al 2017[7] (loc1 = 17.68, scale1 = 2, shape1 = -1) and younger estimates of 12.64 Ma split as proposed by Li et al 2018 (loc2 = 12.64, scale2 = 2, shape2 = 1). Futhermore, we set p to 0.6, accounting a higher likelihood to the distribution around loc1 (see Supplementary Figure 6 for a graphical representation of the S2N distribution):
S2N(0.6,17.68,2,-1,12.64,2,1)

Finally, the input phylogeny for mcmctree is:

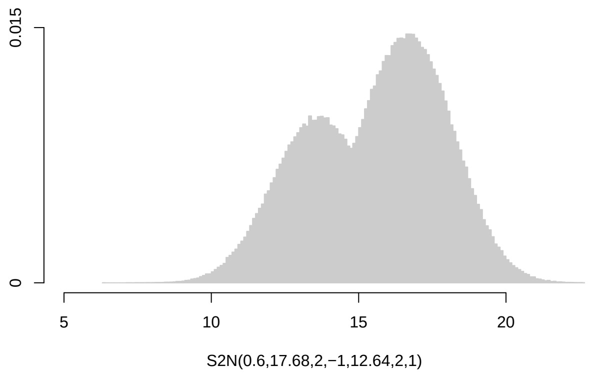

Supplementary Figure 6: Plot of the skew 2 normals distribution S2N(0.6,17.68,2,-1,12.64,2,1) defined as prior for the second calibration point at the split between *Atta* and *Acromyrmex*. x-axis shows divergence times in million years, y-axis shows relative frequencies.

```
(((PCOR,(TSEP,(((AHEY,(ACHA,PARG)),(AECH,AINS)),(ACEP,ACOL))'S2N(0.6,17.68,2,-1,12.64,2,1)')),MZET)'L(0.15,
↪  0.1, 1, 0.1)',CCOS)
```

Based on a substitution rate estimate of 0.394531 (estimated with `baseml`) we calculated shape parameter α and scale parameter β as

$\alpha = (0.394531/0.394531)^2$

$\beta = 0.394531/(0.394531)^2$

The MCMC chain was run with a sample frequency `sampFreq=50000`. We set the rate-drift parameter ($\sigma_2\gamma = 5.5$), otherwise using default priors (gamma-dirichlet prior for locus rates and conditional iid "independent and identically distributed" prior for σ_2).

We ran two mcmc chains and examined convergence of both iterations. Overall, the effective sample size (ESS) ranged between 2,800 to 6,000 in both runs for different nodes in the phylogeny. For run2 the ess was as follows:

	mean.mcmc	ess.mcmc	var.mcmc	se.mcmc
t_n12	0.320456526	4102.949	3.890497e-03	9.737657e-04
t_n13	0.225942071	3138.779	2.907492e-03	9.624515e-04
t_n14	0.158547094	2897.953	1.601081e-03	7.432945e-04
t_n15	0.137668871	2897.458	1.262417e-03	6.600743e-04
t_n16	0.117609921	2984.657	9.804381e-04	5.731429e-04
t_n17	0.052465511	3868.085	2.403652e-04	2.492802e-04
t_n18	0.024947994	4446.807	6.099420e-05	1.171171e-04
t_n19	0.016296529	5146.915	3.138596e-05	7.808978e-05
t_n20	0.009626156	6251.155	1.290330e-05	4.543289e-05
t_n21	0.020463731	5845.835	5.512007e-05	9.710277e-05
mu	0.374347434	3233.345	1.013061e-02	1.770075e-03
sigma2	0.137318608	19301.195	4.741993e-03	4.956651e-04
lnL	-9.525575971	20631.224	9.504064e+00	2.146309e-02

Both MCMC runs yielded very similar results, indicating good convergence and robust estimates of divergence times. Supplementary Figure 7 shows divergence estimates from both runs for each

node, indicating good overlap in posterior estimates in both iterations. We furthermore compared prior and posterior estimates for each variable. For this, we re-ran MCMCtree twice using the same settings, but this time only sampling from the prior for each variable. Supplementary Figure 8 shows that distributions for prior and posterior do not differ considerably. Supplementary Figures 9 and 10 show the phylogenetic trees with divergence estimates generated in the two MCMCtree runs.

The final tree used for subsequent analyses is:

```
Phylogenetic tree with divergence estimates
  (((PCOR: 0.158672, (TSEP: 0.137711, (((AHEY: 0.024983, (ACHA: 0.016335, PARG: 0.016335)
  ↪  [&95%={0.0077802, 0.0293585}]: 0.008648) [&95%={0.0128068, 0.0431821}]: 0.027567, (AECH: 0.009605,
  ↪  AINS: 0.009605) [&95%={0.0044259, 0.0183199}]: 0.042945) [&95%={0.0277912, 0.0878476}]: 0.065115,
  ↪  (ACEP: 0.020492, ACOL: 0.020492) [&95%={0.0094895, 0.0379604}]: 0.097173) [&95%={0.0650718,
  ↪  0.186238}]: 0.020045) [&95%={0.0774672, 0.214752}]: 0.020962) [&95%={0.0902896, 0.244555}]:
  ↪  0.067305, MZET: 0.225977) [&95%={0.133807, 0.340907}]: 0.094428, CCOS: 0.320405) [&95%={0.188064,
  ↪  0.410511}];
```

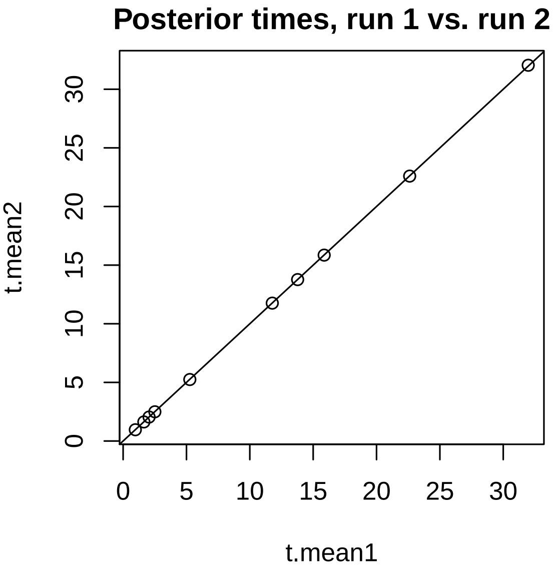

Supplementary Figure 7: Posterior divergence estimates in million years from the first (x-axis) and second run (y-axis) of MCMCtree.

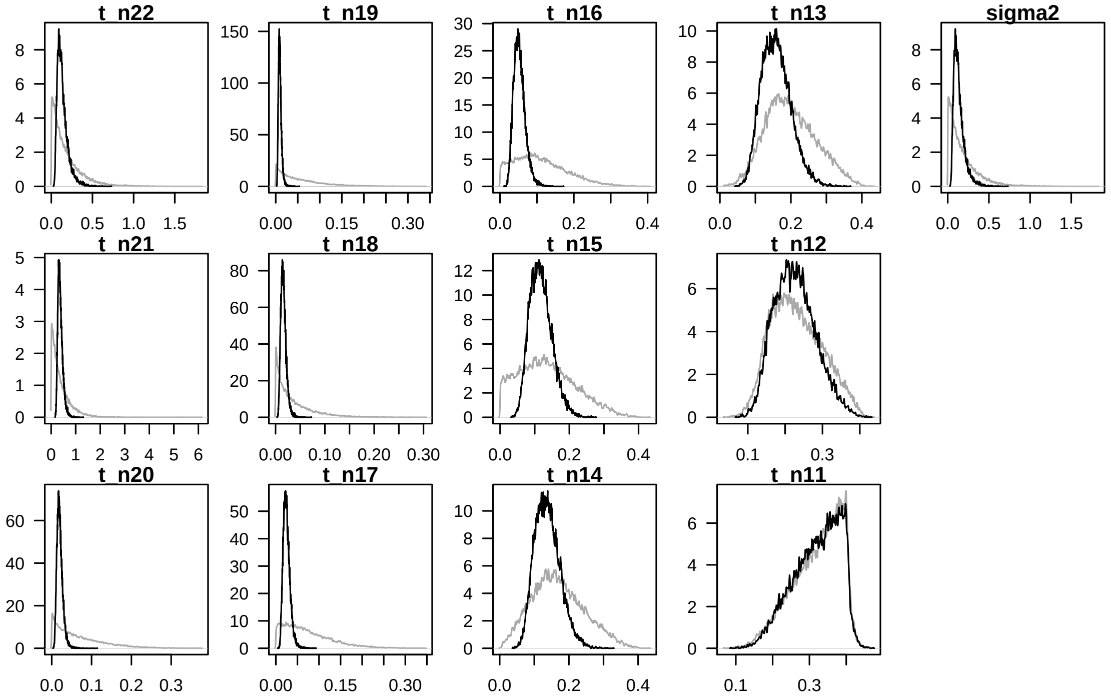

Supplementary Figure 8: Comparison of prior (gray) and posterior estimates (black) from the first run of MCMCtree.

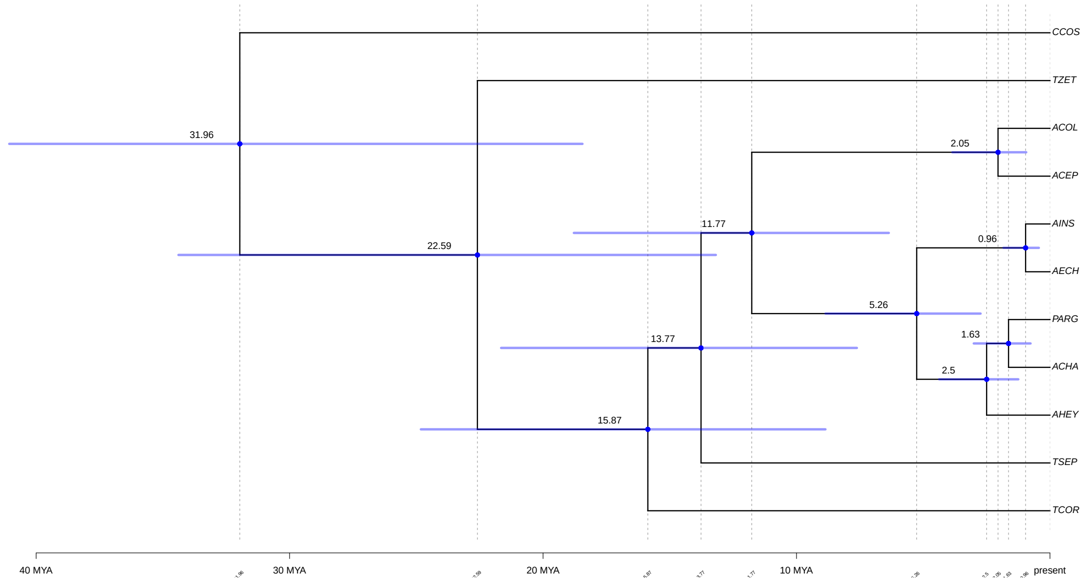

Supplementary Figure 9: Divergence tree estimates produced with MCMCtree, first run. Sampling frequency 50000.

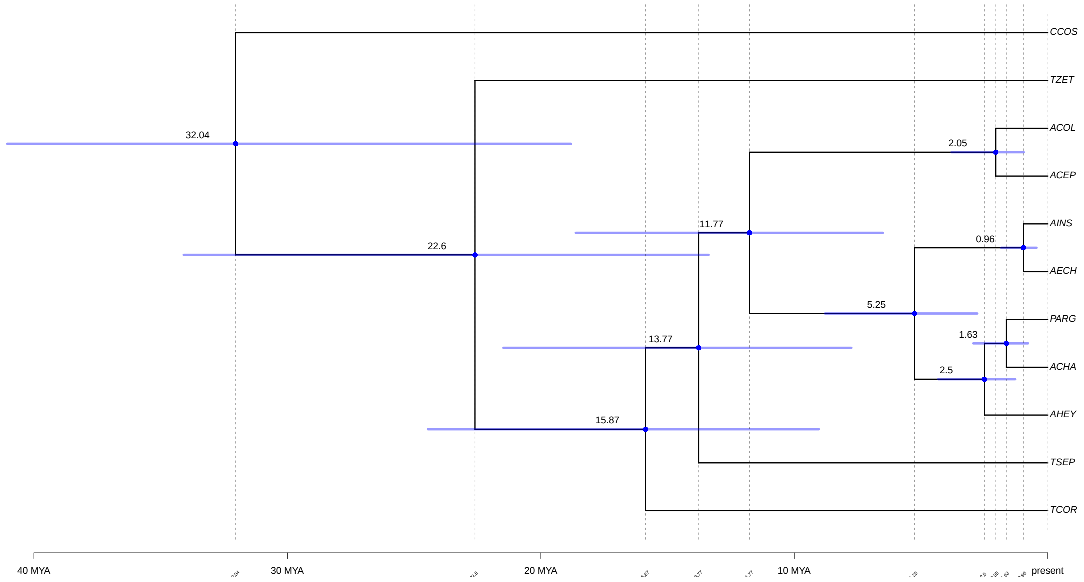

Supplementary Figure 10: Divergence tree estimates produced with MCMCtree, second run. Sampling frequency 50000.

12 Demographic inference

We used MSMC2 v2.1.1 (available at https://github.com/stschiff)[8] to infer effective population sizes across time for *Acromyrmex echinatior*, *Acromyrmex octospinosus* and *Acromyrmex insinuator* from single individual sequencing data of two individuals per species collected in Gamboa, Panama (Table 33). We used short-read (100 bp), small-insert paired end Illumina sequencing aiming to generate ca. 20X coverage per individual. After quality control with trimmomatic 0.39, paired-end mapping to the *A. echinatior* reference genome with bwa v0.7.17-r1188 and duplicate removal with picard, we used samtools, bcftools, and generate_multihetsep.py to generate multihetstep and mask files for all scaffolds and each individual. We restricted our analysis to larger scaffolds over 2 MB and reduced time segments to 1*2+15*1+1*2 to avoid overfitting, following recommendations by Schiffels & Wang (2020)[8].

Supplementary Table 33: Indivual samples used for resequencing

sample ID	Source colony	Population	Species	Collected	Morph
GAi-073	Ae503	Gamboa	A. insinuator	2011	Queen
GAi-020	Ae374	Gamboa	A. insinuator	2008	Queen
GAe-067	Ae459	Gamboa	A. echinatior	2010	Gyne
GAe-077	Ae363	Gamboa	A. echinatior	2008	Gyne
GAo-059	Ao441	Gamboa	A. octospinosus	2010	Gyne
GAo-064	Ao456	Gamboa	A. octospinosus	2010	Gyne

We generated 500 bootstrap replicates from the data and ran MSMC2 for all four haplotypes per species separately and plotted results jointly for each bootstrap replicate. We inferred effective population size per generation based on a mutation rate estimate recently published for bumblebees (3.6e-9)[9] and an approximate generation time of one year. All bootstrap replicates were analyzed and plotted in R. Supplementary Figure 11 shows the results of the MSMC2 analysis for both species. Our analysis shows that the effective population size of *A. insinuator* is substantially and consistently smaller compared to the non-parasitic sister species.

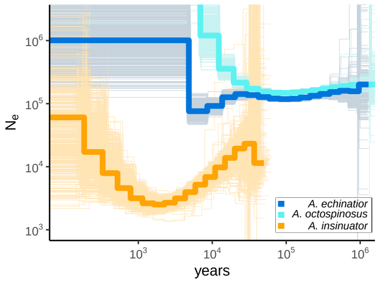

Supplementary Figure 11: Effective population size estimates over past generations for *A. echinatior*, *A. octospinosus* and *A. insinuator*.

13 Signatures of selection

Analyses of signatures of selection were performed on single copy orthologs (SCOs) across the available leaf-cutting ant genomes (*Acromyrmex* and *Atta*). We retrieved 7,750 single-copy orthogroups with `orthofinder`, aligned protein sequences with `prank v.150803` using the inferred species tree as guide tree, and generated CDS alignments with `pal2nal v.14`. Orthogroups that showed significant signs of recombination ($p < 0.01$ as tested with `Phi`) were removed.

We identified genes showing evidence for positive selection by running adaptive branch-site tests (`absREL`, implemented in `HYPHY v2.3.14`) on CDS alignments and the species tree as input. Similarly, we tested for relaxed selection in protein-coding single-copy ortholog genes by running `RELAX` (implemented in `HYPHY v2.3.14`). In `RELAX` we defined test ("T") and reference branches ("R"), aiming to identify genes that showed relaxed selection in the social parasite branches (see Supplementary Figure 12).

```
(((AHEY{R},(ACHA{T},PARG{T})P1{T})A1{R},(AECH{R},AINS{T})A2{R})AO,ACOL)LC;
```

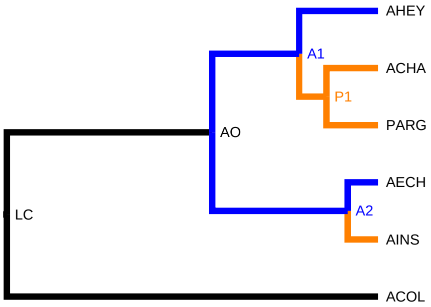

Supplementary Figure 12: Subtree of leaf-cutting ant species used to test for evidence of relaxed selection in social parasite branches. Shown in orange are branches belonging to the test set (P1,ACHA,PARG,AINS). Blue branches (A1,A2,AECH,AHEY) were set as reference.

`RELAX` fits three ω rate categories under the alternative model to the test and reference branch sets and infers a "selection intensity parameter" k, to test for relaxation or intensification along the specified test branches compared to the reference set. $k < 1$ is indicative of relaxed natural selection and $k > 1$ suggests intensification in the test compared to the reference set. The model is fitted so that the first two rate categories ($\omega_{1,2}$) summarize regions in the coding sequence with $\omega < 1$ (i.e. purifying selection) and the third category (ω_3) summarizes regions with $\omega > 1$ (i.e. positive selection). We calculated background omega ratios ω_{bg} from ω_1 and ω_2 for each gene for the test and reference branch sets (as $\omega_{bg} = (proportion_1 * \omega_1) + (proportion_2 * \omega_2)$), and tested whether these background evolutionary rates are significantly different between the test and reference sets. Similarly, we compared ω_3 rates between both branch sets. Finally, we asked whether social parasite branches show evidence of an overall relaxation of selection, by testing for a significant deviation of $log_2(k)$ from 0. Statistical analyses were conducted in `R` using permutation tests with 20,000 samples (`R`-functions `wilcox_test()` from the

library coin for paired two-sample tests on ω_{bg} and ω_3 and perm.test() from the library jmuOutlier for one-sample tests). Together, our analysis showed that both, background omega rates (ω_{bg}) and ω_3 rates were significantly closer to 1 in the social parasite branches compared to the host branches (Fig. 13A). Concordantly, we found that k was on average shifted to $k < 1$ (i.e. $log_2(k) < 0$, Supplementary Figure 13C). For comparing average evolutionary rates across hosts and parasites, we removed genes with average $dN/dS > 10$ in either the test or the reference set.

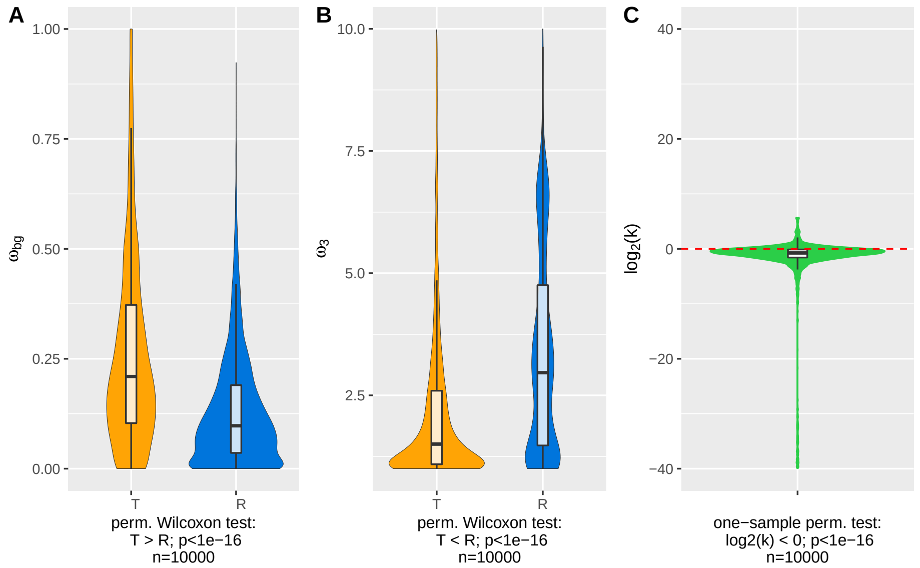

Supplementary Figure 13: Summary of omega rate class differences between test (PARG, P1, ACHA, AINS) and reference (AHEY, AECH, A2) branch sets (see Supplementary Figure 12). (A) Background ω rates in test (red) and reference branch sets (blue). (B) Third ω rate class for test (red) and reference branch sets (blue). (C) Distribution of selection intensity parameter $log_2(k)$ as a measure of intensification ($log_2(k) > 0$) or relaxation ($log_2(k) < 1$) of natural selection in the test set. The small inlet plot in the top right corner is a focus on $k = -2$ to $k = 2$. Boxplot centers show the median, hinges show the first and third quantiles, and whiskers show the 1.5 x inter-quantile range. Statistical significance was assessed using two-sided permutated Wilcoxon-Mann-Whitney tests (A and B) and two-sided one-sample permutation tests (C)

RELAX identified 233 genes showing significant signs of relaxed selection acting in social parasite branches, with $k < 1$ and $fdr < 0.1$ (82 with $fdr < 0.05$). Only 102 genes showed a significant intensification of selection in social parasite branches ($k > 1$ and $fdr < 0.1$ (56 with $fdr < 0.05$)). We tested for gene ontology term enrichment across those genes showing significantly relaxed selection using the parentChild algorithm implemented in the R-package topGO, comparing GO annotations of the gene set to all tested single copy orthologs. Fifety five GO terms showed significant enrichment in one of the three annotation domains ($p < 0.05$, 9 Molecular Function, 32 Biological Process, 14 Cellular Component, Supplementary Table 34). Conversely, among the 102 genes showing significant intensification in social parasite branches enrichment analyses found 21 significantly enriched GO terms ($p < 0.05$, 7 Molecular Function, 6 Biological Process, 8 Cellular Component, Supplementary Table 35). However, after correcting for mutliple testing, no GO terms were enriched at $FDR < 0.05$ among genes showing significant intensification of selection or relaxation of selection in the social parasites.

Testing more explicitly for evidence of positive selection in inquiline social parasite branches using

Supplementary Table 34: Significant Gene Ontology term enrichment of genes under relaxed selection ($k < 1$, $FDR < 0.1$) at inquiline social parasite branches, compared to Ahey, Aech, A1 and A2.

GO.ID	Term	Annotated	Significant	Expected	parentChild	ontology
**GO:0140096**	catalytic activity, acting on a protein	483	26	17.18	0.00044	MF
**GO:0005085**	guanyl-nucleotide exchange factor activi...	34	5	1.21	0.00159	MF
**GO:0005515**	protein binding	1329	72	47.27	0.00296	MF
**GO:1901265**	nucleoside phosphate binding	579	25	20.59	0.00881	MF
**GO:0005158**	insulin receptor binding	4	2	0.14	0.01762	MF
**GO:0044877**	protein-containing complex binding	16	3	0.57	0.02843	MF
**GO:0019787**	ubiquitin-like protein transferase activ...	34	4	1.21	0.03523	MF
**GO:0016298**	lipase activity	11	2	0.39	0.04137	MF
**GO:0005217**	intracellular ligand-gated ion channel a...	1	1	0.04	0.04348	MF
**GO:0035556**	intracellular signal transduction	166	12	4.98	0.0024	BP
**GO:0019219**	regulation of nucleobase-containing comp...	253	11	7.59	0.0028	BP
**GO:0050789**	regulation of biological process	692	32	20.75	0.0038	BP
**GO:0006793**	phosphorus metabolic process	327	19	9.8	0.0045	BP
**GO:0046903**	secretion	17	3	0.51	0.0063	BP
**GO:0065007**	biological regulation	719	32	21.56	0.0071	BP
**GO:0036211**	protein modification process	351	21	10.52	0.0100	BP
**GO:0043412**	macromolecule modification	372	21	11.15	0.0141	BP
**GO:0051049**	regulation of transport	9	2	0.27	0.0180	BP
**GO:0032940**	secretion by cell	17	3	0.51	0.0182	BP
**GO:0070646**	protein modification by small protein re...	20	3	0.6	0.0184	BP
**GO:1903530**	regulation of secretion by cell	5	2	0.15	0.0192	BP
**GO:0051252**	regulation of RNA metabolic process	249	11	7.47	0.0227	BP
**GO:0050794**	regulation of cellular process	676	31	20.27	0.0230	BP
**GO:0009914**	hormone transport	1	1	0.03	0.0245	BP
**GO:0023061**	signal release	4	2	0.12	0.0247	BP
**GO:0071704**	organic substance metabolic process	1508	53	45.21	0.0263	BP
**GO:0048869**	cellular developmental process	8	2	0.24	0.0278	BP
**GO:0070201**	regulation of establishment of protein l...	1	1	0.03	0.0308	BP
**GO:0010468**	regulation of gene expression	264	12	7.92	0.0311	BP
**GO:0051223**	regulation of protein transport	1	1	0.03	0.0315	BP
**GO:0006464**	cellular protein modification process	351	21	10.52	0.0337	BP
**GO:0032880**	regulation of protein localization	1	1	0.03	0.0357	BP
**GO:0006935**	chemotaxis	1	1	0.03	0.0357	BP
**GO:0044271**	cellular nitrogen compound biosynthetic ...	499	15	14.96	0.0363	BP
**GO:0006629**	lipid metabolic process	91	7	2.73	0.0364	BP
**GO:0044260**	cellular macromolecule metabolic process	907	37	27.19	0.0373	BP
**GO:0090087**	regulation of peptide transport	1	1	0.03	0.0373	BP
**GO:0097485**	neuron projection guidance	1	1	0.03	0.0385	BP
**GO:0008614**	pyridoxine metabolic process	2	1	0.06	0.0407	BP
**GO:0051641**	cellular localization	120	6	3.6	0.0441	BP
**GO:0009987**	cellular process	1820	62	54.57	0.0471	BP
**GO:0043226**	organelle	599	27	18.15	0.0065	CC
**GO:0016459**	myosin complex	14	3	0.42	0.0109	CC
**GO:0005856**	cytoskeleton	87	7	2.64	0.0170	CC
**GO:0044424**	intracellular part	757	31	22.94	0.0360	CC
**GO:0005942**	phosphatidylinositol 3-kinase complex	2	1	0.06	0.0419	CC
**GO:0005643**	nuclear pore	10	2	0.3	0.0483	CC

Supplementary Table 35: Significant Gene Ontology term enrichment of genes under intensified selection ($k > 1$, $FDR < 0.1$) at inquiline social parasite branches, compared to Ahey, Aech, A1 and A2.

GO.ID	Term	Annotated	Significant	Expected	parentChild	ontology
**GO:0004001**	adenosine kinase activity	1	1	0.01	0.0047	MF
**GO:0004427**	inorganic diphosphatase activity	1	1	0.01	0.0196	MF
**GO:0003676**	nucleic acid binding	612	11	8.4	0.0199	MF
**GO:0003796**	lysozyme activity	1	1	0.01	0.0217	MF
**GO:0000287**	magnesium ion binding	22	2	0.3	0.0231	MF
**GO:0003998**	acylphosphatase activity	1	1	0.01	0.0258	MF
**GO:0061783**	peptidoglycan muralytic activity	3	1	0.04	0.0478	MF
**GO:0031929**	TOR signaling	4	2	0.06	0.0026	BP
**GO:0038179**	neurotrophin signaling pathway	1	1	0.01	0.0169	BP
**GO:0015748**	organophosphate ester transport	5	1	0.07	0.0318	BP
**GO:0043094**	cellular metabolic compound salvage	3	1	0.04	0.0433	BP
**GO:0043101**	purine-containing compound salvage	2	1	0.03	0.0443	BP
**GO:0034404**	nucleobase-containing small molecule bio...	18	2	0.27	0.045	BP
**GO:0044441**	ciliary part	2	1	0.03	0.0074	CC
**GO:0005929**	cilium	2	1	0.03	0.0133	CC
**GO:0034464**	BBSome	1	1	0.01	0.015	CC
**GO:0038201**	TOR complex	2	1	0.03	0.021	CC
**GO:0071203**	WASH complex	2	1	0.03	0.0246	CC
**GO:0042995**	cell projection	2	1	0.03	0.029	CC
**GO:0044463**	cell projection part	2	1	0.03	0.029	CC
**GO:0031932**	TORC2 complex	2	1	0.03	0.0297	CC

`absREL`, we identified 122 genes with evidence for positive selection in PARG, 101 genes in AINS, 71 in ACHA, and 14 in P1 (at $FDR < 0.05$). Only a single orthogroup (OG0004128) showed evidence for positive selection in three of the four tested branches. This orthogroup codes for a chromodomain-helicase-DNA-binding protein (chromodomain-helicase-DNA-binding protein 7; EC:3.6.4.12). Nine orthogroups showed positive selection in two of four branches. Our analyses with `absREL` further revealed that median $omega$ is higher in social parasite branches compared to non-parasitic *Acromyrmex*: $A1 = 0.157$, $A2 = 0.116$, $ACHA = 0.126$, $ACOL = 0.104$, $AECH = 0.065$, $AHEY = 0.091$, $AINS = 0.130$, $P1 = 0.162$, $PARG = 0.261$. The results of pairwise comparisons between parasites and non-parasitic lineages using Wilcoxon rank sum test with continuity correction (with Holm correction) were as follows: AINS vs AECH: $p = 5.7e - 05$, P1 vs AHEY: $p < 2e - 16$, ACHA vs AHEY: $p = 1.3e - 05$, PARG vs AHEY: $p < 2e - 16$. Similar to the approach above, we removed any gene with $dN/dS > 10$ in any branch.

Across the different branches, between 14 and 241 genes dN/dS was larger than one (Table 36).

Supplementary Table 36: Number of genes with $dN/dS > 1$ in the different branches (branch labels as in Supplementary Figure 12).

branch	genes with $dN/dS > 1$
A1	241
A2	100
ACHA	100
ACOL	14
AECH	123
AHEY	100
AINS	145
P1	97
PARG	215

Supplementary Table 37: Orthogroups showing signatures of positive selection in two of the four inquiline social parasite branches.

OGid	ACHA	AINS	PARG	swissprot	kegg	ACHAfdr	AINSfdr	P1fdr	PARGfdr
**OG0001421**	ACHA00508	AINS09383	PARG05629	Cyclic nucleotide-gated olfactory channel	K04950	4.81E-10	1.57E-05	1	1.0000
**OG0003734**	ACHA04891	AINS09460	PARG07155	Aldehyde dehydrogenase, dimeric NADP-preferring	K00128	4.75E-05	1.0000	1	0.0000
**OG0004128**	ACHA05506	AINS13567	PARG03090	Chromodomain-helicase-DNA-binding protein 9	K14437	0	2.23E-06	1	0.0106
**OG0004852**	ACHA07090	AINS14158	PARG03630	NA		0.0409	1.0000	1	0.0010
**OG0005133**	ACHA07673	AINS05968	PARG09312	Repressor of RNA polymerase III transcription MAF1 homolog		0.0002	2.00E-08	1	1.0000
**OG0005166**	ACHA07770	AINS12762	PARG08218	Claspin		1.0000	1.0000	0.0024	0.0001
**OG0006124**	ACHA09566	AINS13183	PARG04646	SLIT-ROBO Rho GTPase-activating protein 1	K07526	1.0000	3.43E-06	1	0.0002
**OG0006393**	ACHA10251	AINS10282	PARG00219	EF-hand calcium-binding domain-containing protein 14	K14570	1.0000	0.0011	1	5.76E-06
**OG0006830**	ACHA11372	AINS12687	PARG08016	Receptor-type tyrosine-protein phosphatase kappa	K13297	0.0067	1.15E-08	1	1.0000

We again used topGO to test for significantly enriched GO terms among the orthogroups showing positive selection, focusing on the two origins of inquilinism (AINS and P1). Fourty seven GO terms showed significant enrichment in one of the three annotation domains ($p < 0.05$, 7 Molecular Function, 27 Biological Process, 13 Cellular Component, Supplementary Table 38). After correcting for multiple testing, no GO terms showed enrichment at $FDR < 0.05$.

Supplementary Table 38: Significant Gene Ontology term enrichment of genes under positive selection ($FDR < 0.1$) at either of the social parasite origins.

GO.ID	Term	Annotated	Significant	Expected	parentChild	ontology
**GO:0003774**	motor activity	33	4	0.65	0.023	MF
**GO:0008276**	protein methyltransferase activity	12	2	0.24	0.028	MF
**GO:0016817**	hydrolase activity, acting on acid anhyd...	155	7	3.05	0.029	MF
**GO:0005515**	protein binding	1299	36	25.59	0.035	MF
**GO:0005215**	transporter activity	238	9	4.69	0.042	MF
**GO:0043395**	heparan sulfate proteoglycan binding	2	1	0.04	0.043	MF
**GO:0043394**	proteoglycan binding	2	1	0.04	0.049	MF
**GO:0006928**	movement of cell or subcellular componen...	24	3	0.38	0.0062	BP
**GO:0048583**	regulation of response to stimulus	49	4	0.77	0.0072	BP
**GO:1901564**	organonitrogen compound metabolic proces...	802	15	12.54	0.0095	BP
**GO:0006359**	regulation of transcription by RNA polym...	3	1	0.05	0.0123	BP
**GO:0023051**	regulation of signaling	52	4	0.81	0.0126	BP
**GO:0019538**	protein metabolic process	638	13	9.97	0.0129	BP
**GO:0010646**	regulation of cell communication	51	4	0.8	0.0129	BP
**GO:0006022**	aminoglycan metabolic process	43	3	0.67	0.0166	BP
**GO:0007165**	signal transduction	352	11	5.5	0.0169	BP
**GO:0006383**	transcription by RNA polymerase III	5	1	0.08	0.0175	BP
**GO:0023052**	signaling	363	11	5.67	0.0201	BP
**GO:0007154**	cell communication	361	11	5.64	0.0205	BP
**GO:0006040**	amino sugar metabolic process	38	3	0.59	0.0253	BP
**GO:0007017**	microtubule-based process	40	3	0.63	0.0255	BP
**GO:0007265**	Ras protein signal transduction	29	3	0.45	0.0264	BP
**GO:0031098**	stress-activated protein kinase signalin...	1	1	0.02	0.0346	BP
**GO:0080135**	regulation of cellular response to stres...	2	1	0.03	0.0357	BP
**GO:0045892**	negative regulation of transcription, DN...	11	1	0.17	0.037	BP
**GO:1902679**	negative regulation of RNA biosynthetic ...	11	1	0.17	0.0378	BP
**GO:1903507**	negative regulation of nucleic acid-temp...	11	1	0.17	0.0386	BP
**GO:0030705**	cytoskeleton-dependent intracellular tra...	2	1	0.03	0.0386	BP
**GO:0009966**	regulation of signal transduction	47	4	0.73	0.043	BP
**GO:0099111**	microtubule-based transport	2	1	0.03	0.0474	BP
**GO:0051253**	negative regulation of RNA metabolic pro...	11	1	0.17	0.0499	BP
**GO:0044430**	cytoskeletal part	63	6	1.17	0.00059	CC
**GO:0043232**	intracellular non-membrane-bounded organ...	189	8	3.52	0.00643	CC
**GO:0043228**	non-membrane-bounded organelle	189	8	3.52	0.01369	CC
**GO:0030496**	midbody	1	1	0.02	0.01831	CC
**GO:0000346**	transcription export complex	1	1	0.02	0.02771	CC
**GO:0000347**	THO complex	1	1	0.02	0.02771	CC
**GO:0034464**	BBSome	1	1	0.02	0.02806	CC
**GO:0038201**	TOR complex	2	1	0.04	0.03466	CC
**GO:0005875**	microtubule associated complex	26	3	0.48	0.03469	CC
**GO:0042995**	cell projection	2	1	0.04	0.0363	CC
**GO:0044463**	cell projection part	2	1	0.04	0.0363	CC
**GO:0005929**	cilium	2	1	0.04	0.0403	CC
**GO:0005576**	extracellular region	76	4	1.42	0.04944	CC

We further analysed GO enrichment analyses results using REVIGO (http://revigo.irb.hr/), summarizing GO terms with $p < 0.1$ for each annotation domain (semantic similarity = SimRel, GO term sizes derived from Uniprot) (Figures 14, 15, 16 show REVIGO plots of GO enrichment analyses on genes under relaxed selection ($p < 0.1$, $k < 1$) in social parasite branches compared to the hosts; Supplementary Figures 17, 18, 19 show REVIGO plots of GO enrichment analyses on genes under intensified ($p < 0.1$, $k > 1$) selection in social parasite branches compared to the hosts). Among genes under relaxed selection, we find an overrepresentation of several potentially interesting terms, such as "insulin receptor binding", "lipase activity", "locomotion", "secretion", "regulation of synaptic plasticity", and "lipid

metabolic process”. Similarly, among genes under positive selection, we find an overrepresentation of a few potentially interesting terms such as ”response to stimulus” and ”TOR complex”.

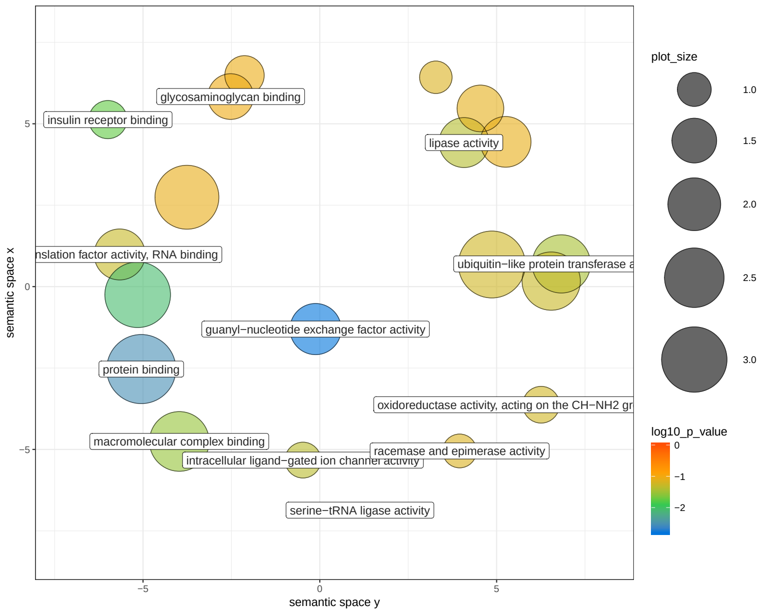

Supplementary Figure 14: REVIGO plot summarizing Biological Process gene ontology terms enriched among genes under relaxed selection in inquilines. Included are GO terms at $p < 0.1$ (according to parentChild tests, see above).

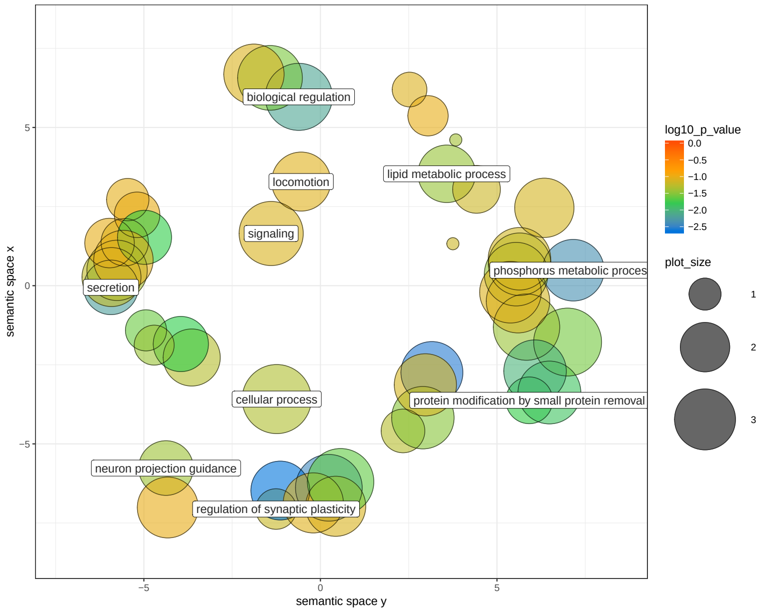

Supplementary Figure 15: REVIGO plot summarizing Molecular Function gene ontology terms enriched among genes under relaxed selection in inquilines. Included are GO terms at $p < 0.1$ (according to parentChild tests, see above).

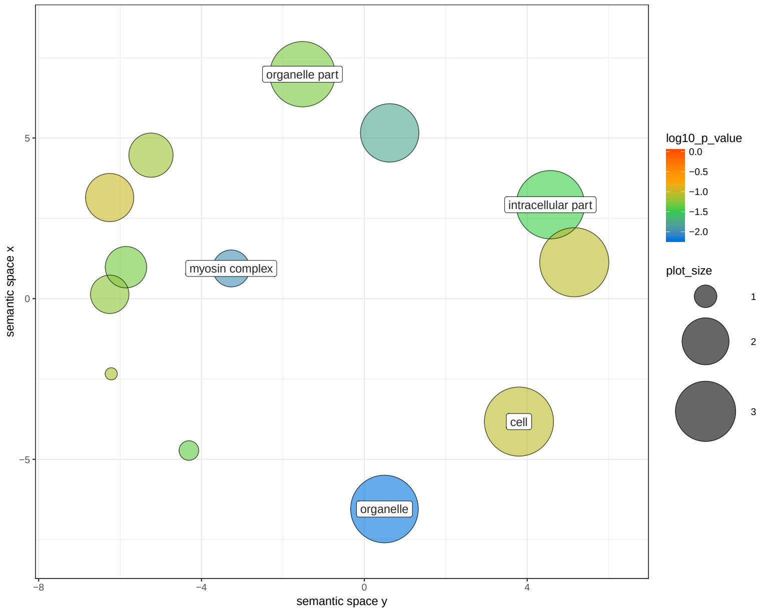

Supplementary Figure 16: REVIGO plot summarizing Cellular Component gene ontology terms enriched among genes under relaxed selection in inquilines. Included are GO terms at $p < 0.1$ (according to parentChild tests, see above).

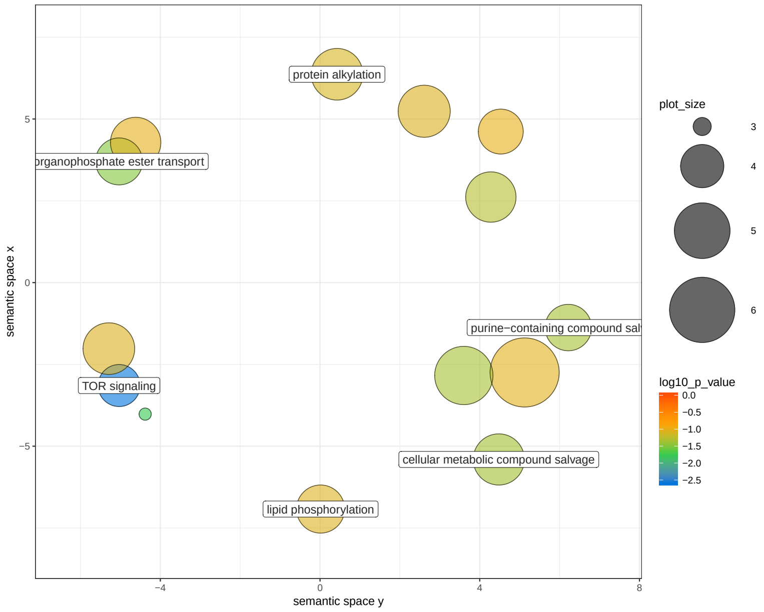

Supplementary Figure 17: REVIGO plot summarizing Biological Process gene ontology terms enriched among genes under intensified selection in inquilines. Included are GO terms at $p < 0.1$ (according to parentChild tests, see above).

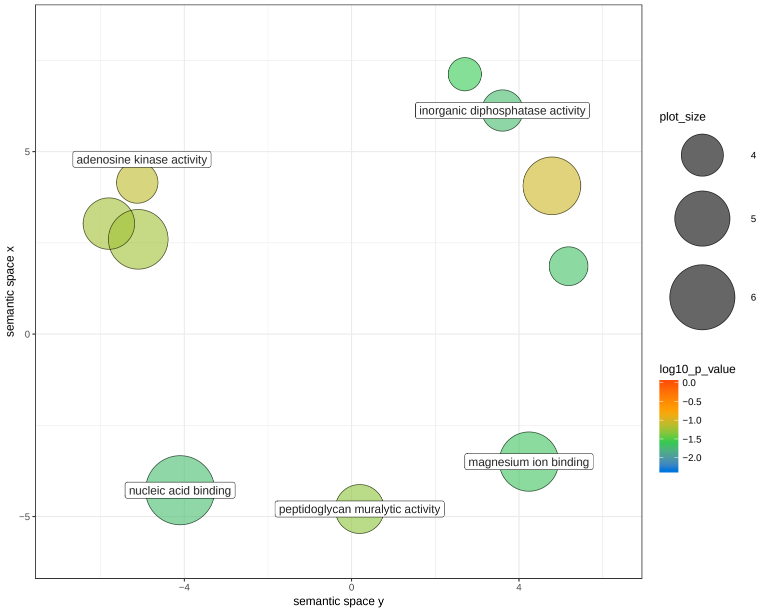

Supplementary Figure 18: REVIGO plot summarizing Molecular Function gene ontology terms enriched among genes under intensified selection in inquilines. Included are GO terms at $p < 0.1$ (according to parentChild tests, see above).

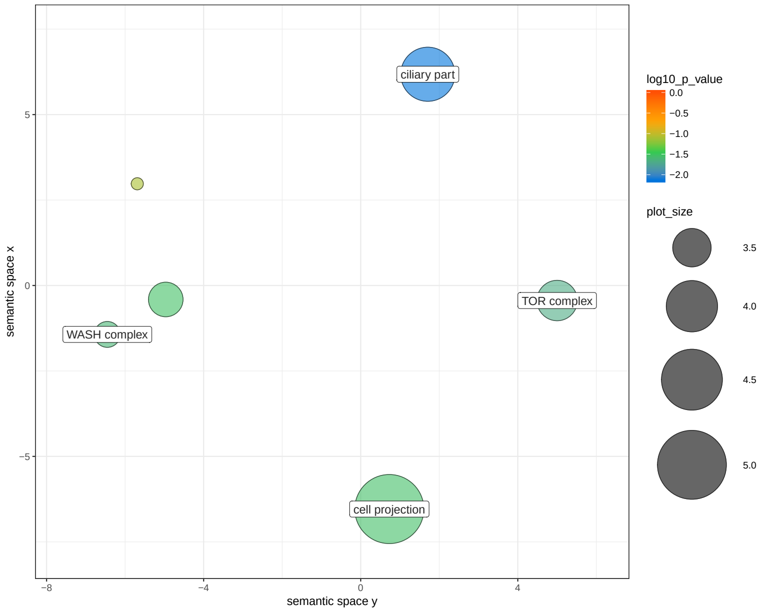

Supplementary Figure 19: REVIGO plot summarizing Cellular Component gene ontology terms enriched among genes under intensified selection in inquilines. Included are GO terms at $p < 0.1$ (according to parentChild tests, see above).

14 Codon Usage Bias

Codon usage bias (CUB, i.e. the preferential usage of certain codons) can be affected by genetic drift, mutation bias, or selection for efficient ribosomal usage. To explore CUB in our study species, we analysed coding sequences of 6966 single-copy orthologs in all hosts and parasites. We calculated GC content at the first (GC1), second (GC2), and third (GC3) codon positions, as well as the effective number of codons (ENC) for each CDS in each species. The ENC is a measure to assess the degree at which all 61 codons are used in a given gene, with extremely biased genes having an ENC of 20 and unbiased genes having an ENC of 61.

In general, CUB is weak in all hosts and parasites, with median ENC ranging from 54.99 in *A. echinatior* to 55.23 in *P. argentina* (Fig. 20). Cluster analysis of all ENC across all orthologs showed that species cluster largely according to their phylogenetic relationship, suggesting that species specific differences are minor and that there is no detectable convergence of CUB in the parasite species (Fig. 21). Reduced efficiency of selection in social parasites should negatively affect CUB, i.e. reduce ENC across genes. $ENC \sim GC3$ plots are used to explore to what extent CUB is dominated by mutational bias or by natural selection. Under strict mutational bias, the relationship between ENC and GC3 is expected to be: $ENC = 2 + GC3 + 29/(GC3^2 + (1 - GC3)^2)$ (Ref. 10).

Deviation of the observed relationship from this expectation is considered to be due to the effect of natural selection on CUB. Across the three *Acromymrex* inquiline parasites and their two host, the average distance between oberved and expected values (R) only differs marginally between species (Fig. 22, left panels). While R was indeed consistently smaller in all parasites compared to their respective hosts, these differences were non-significant ($p > 0.05$) according to pairwise Mann-Whitney tests.

Similary, neutrality plots (Fig. 22, right panels) that compare GC12 (i.e. predominantly non-synonymous sites) and GC3 (i.e. predominantly synonymous sites) of single-copy orthologs in the different species revealed that the regression coefficient (i.e. the slope) of the $GC12 \sim GC3$ relationship is slightly higher in all parasites compared to their respective hosts. The regression coefficient here provides a measure of the neutrality of the first and second codon position relative to the third[11]. Hence, if the second and first codon positions evolve more neutrally, the slope will be closer to 1, as seen in *P. argentina* and *A. echinatior* relative to their hosts. The regression coefficient of the neutrality plot ranges between 0.25 and 0.265 for all species, suggesting again that natural selection plays an important role in shaping codon usage in these species. Finally, while a comparison between dN/dS estimates for hosts and parasites shows a clear shift towards relatively higher dN/dS in parasites (in-

dicative of relaxed selection), no such trend is apparent for ENC. ENC, in fact, is highly correlated between hosts and parasites, with a correlation coefficient of ~ 1 across all single-copy orthologs (Fig. 23). Overall, our results are consistent with a recent study of CUB in different *Pseudomyrmex* species, which reported the strongest bias in a mutualistic species compared to non-mutualists, but also no significant differences in CUB across all mutualistic vs non-mutualistic species.[12] Taken together, these data indicate that CUB is conserved in ants in general, only mildy affected by their particular life history, and only weakly correlated with patterns observed at the level of synonymous and non-synonymous site evolution.

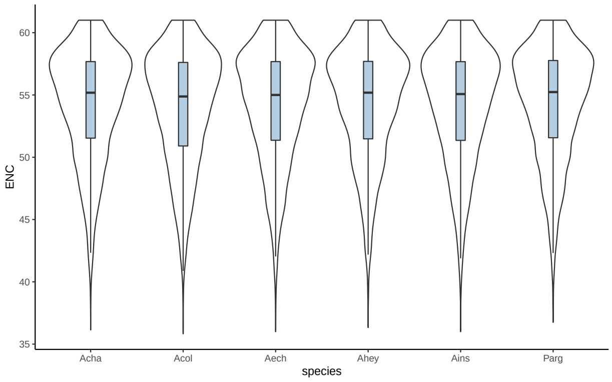

Supplementary Figure 20: Distribution of the effective number of codons (ENC) in different species across single-copy orthologs. Boxplot centers show the median, hinges show the first and third quantiles, and whiskers show the 1.5 x inter-quantile range.

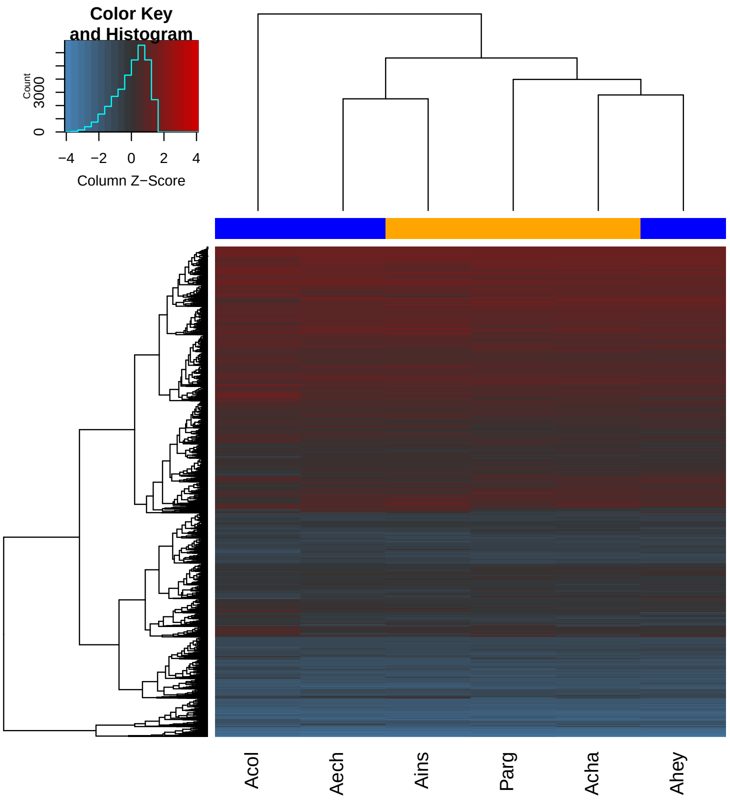

Supplementary Figure 21: Heatmap and hierachical clustering of ENC across single copy-orthologs. Species labelled in orange are inquiline social parasites.

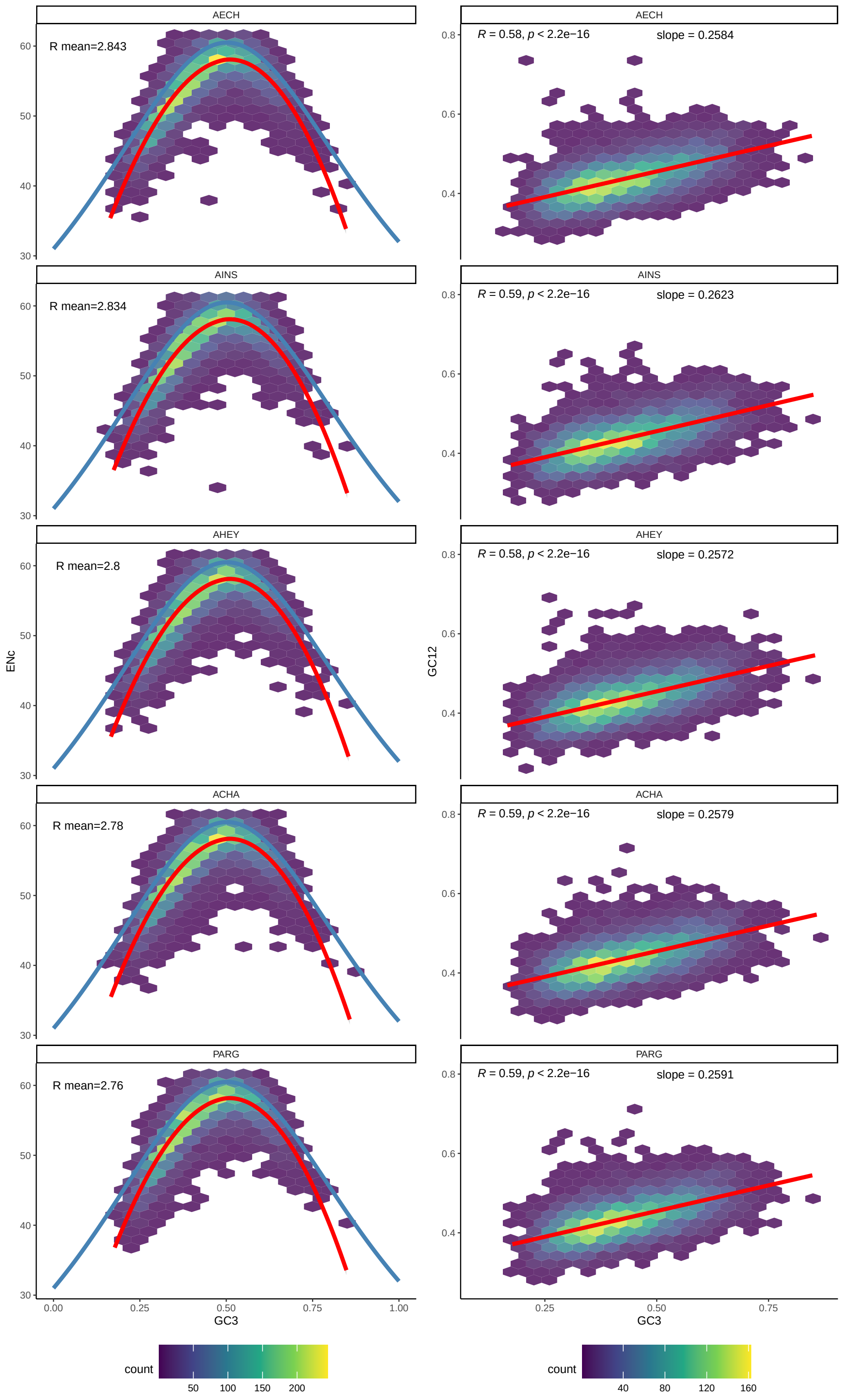

Supplementary Figure 22: $ENC \sim GC3$ (left panels) and neutrality plots (right panels) for *Acromyrmex* hosts and their inquiline parasites. Mean R values in the $ENC \sim GC3$ plots quantify the average deviation from the relationship between ENC and GC3 expected under strict neutrality. Slopes given in the neutrality plots show correlation coefficients for $GC3 \sim GC12$. Values closer to 1 are indicative of a weaker effect of natural selection. R- and p-values for neutrality plots according to Pearson correlations.

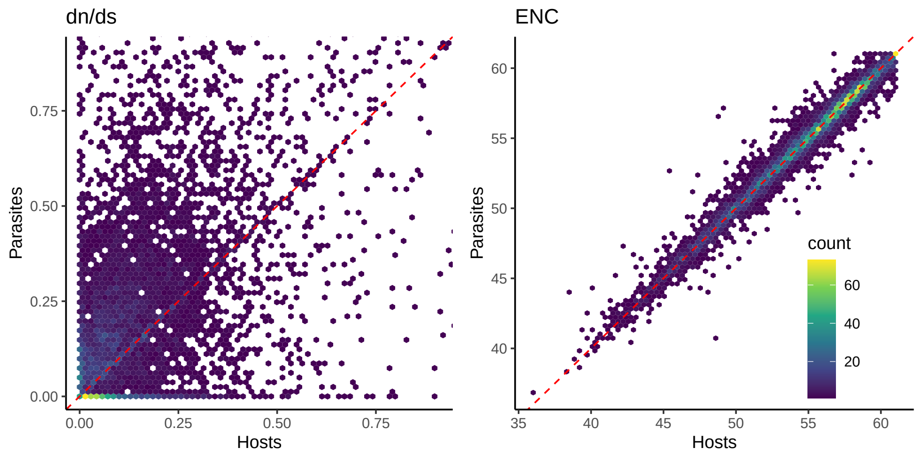

Supplementary Figure 23: Average dN/dS (left plot) and ENC (right plot) for single-copy orthologs in hosts (x-axis) and parasites (y-axis).

15 Gene family size evolution

15.1 Gene family clustering with MCL

We computed gene family clusters between all available genomes from *Acromyrmex* species and *Atta colombica* using `blastp` all-vs-all searches across predicted proteomes and subsequent MCL clustering (`MCL v.14-137`). `blastp v.2.6.0+` was run using a e-value cutoff of $1e-5$. We tested different inflation parameters, ranging from 1.0 to 3.0 and eventually opted for setting `I=1.5` for the final clustering. We restricted our analysis on *Acromyrmex* species and *Atta colombica* as these genomes were sequenced using identical approaches (long-range Illumina libraries, while *At. cephalotes* was assembled from 454 data only). We also excluded non-leafcutting species of attines to reduce parameterization complexity of the modelling. Following gene family clustering, we excluded gene families that contained proteins identified as transposable element (TE) proteins or TE-derived proteins, based on annotations of all proteomes done with `TransposonPSI v.08222010` (39).

Supplementary Table 39: Number of genes coding for transposable-element related proteins, according to annotation with TransposonPSI.

Species	Number of TE-related proteins
**Acep**	385
**Acol**	943
**Aech**	1068
**Acha**	1172
**Ahey**	1269
**Ains**	990
**Ccos**	1022
**Parg**	411
**Pcor**	953
**Tsep**	1544
**Mzet**	1707

The final set of gene families comprised 16,988 clusters. The largest gene family contained 286 proteins, of which 123 were annotated as `IPR013604; 7TM chemoreceptor`, according to IPRscan annotations. 5479 of the gene families were single-copy-orthologs present in each of the analysed

genomes. We furthermore identified gene families with two (n=254), three (n=63), four (n=23), five (n=7), six (n=4), seven (n=1; annotated as `IPR011705; BTB/Kelch-associated`), eight (n=2), and nine (n=1; annotated as `IPR018000; Neurotransmitter-gated ion-channel`) copies. We finally removed one cluster with more than 100 members in a single species (Cluster31 with 115 genes in *P. argentina* and a total cluster size of 140), following recommended practices for gene family size evolution analyses. Using `CAFE v4.0`, we modeled gene family size evolution in *Acromyrmex* host and social parasite genomes based on gene families clusters predicted with MCL. `CAFE` allows to account for assembly and annotation errors of the analysed genomes, by iteratively estimating error rates in the input data sets based on likelihood score optimizations under varying error models. We ran error estimation several times in order to identify global optima in the likelihood landscape. Final error estimates used for the subsequent modelling of gene family size evolution are:

```
Final error estimates by species:
PARG                            0.129265136719
ACOL                            0.0
AINS                            0.0430883789063
AHEY                            0.0478759765625
ACHA                            0.0287255859375
AECH                            0.0526635742187
Score with individual errors:   27970.041073
Lambda with individual errors:  0.00048795590293
=====================================================================
***********************************
Score with no errormodel:       30193.176929
Lambda with no errormodel:      0.00095486428317
***********************************
Global Error Estimation:        0.0478759765625
Score with global errormodel:   28615.716574
Lambda with global errormodel:  0.00052122244019
```

`CAFE` was run on the dataset comprising the ultrametric species divergence tree (generated with `MCMCtree`), the estimated species-specific error rates, and the gene family clustering. After fully parameterized models repeatedly failed to converge in `CAFE`, we decided to run all possible variations of 2-parameter models, to estimate branch-specific λ and μ values. All 2-parameter models were run independtly eight times to produce robust parameter estimates.

We subsequently selected λ and μ estimates for each branch from the best-scoring run (given in Supplementary Table 40). Estimates were highly consistent for most branches across all iterations of modelling (Fig. 24).

The `cafescript` for each 2-parameter models was ran as given in the following example for model `(((2,(1,1)1)1,(1,1)1)1,1)`:

```
tree
↪ (((AHEY:0024.983,(ACHA:0016.335,PARG:0016.335):0008.648):0027.567,(AECH:0009.605,AINS:0009.605):0042.945):0065.115,ACOL:0117.665);
```

Supplementary Table 40: λ and μ parameter estimates from best-scoring Maximum Likelihood modelling runs for all eleven branches. lambda1 and mu1 give estimates for all but the branches of interest. lambda2 and mu2 provide optimized estimates for the branches of interest. See also Supplementary Figure 24.

Node	lambda1	lambda2	mu1	mu2	likelihood score	tree
ACHA	0.0005417	0.0015091	0.0003920	0.0006382	27893.5	(((1,(2,1)1)1,(1,1)1)1,1)
ACHA/PARG	0.0005950	0.0000702	0.0003664	0.0027303	27830.8	(((1,(1,1)2)1,(1,1)1)1,1)
ACOL	0.0006378	0.0002086	0.0008387	0.0000000	27239.5	(((1,(1,1)1)1,(1,1)1)1,2)
ACRO	0.0005468	0.0006655	0.0004670	0.0003129	27935.3	(((1,(1,1)1)1,(1,1)1)2,1)
AECH	0.0005267	0.0024262	0.0003967	0.0014861	27842.8	(((1,(1,1)1)1,(2,1)1)1,1)
AECH/AINS	0.0006333	0.0002444	0.0004382	0.0003243	27906.5	(((1,(1,1)1)1,(1,1)2)1,1)
AHEY	0.0004672	0.0019799	0.0003724	0.0009411	27732.9	(((2,(1,1)1)1,(1,1)1)1,1)
AHEY/ACHA/PARG	0.0006201	0.0002469	0.0004021	0.0004770	27929.3	(((1,(1,1)1)2,(1,1)1)1,1)
AINS	0.0005533	0.0007415	0.0003944	0.0033389	27828.8	(((1,(1,1)1)1,(1,2)1)1,1)
PARG	0.0005397	0.0003262	0.0002638	0.0075786	26886.4	(((1,(1,2)1)1,(1,1)1)1,1)

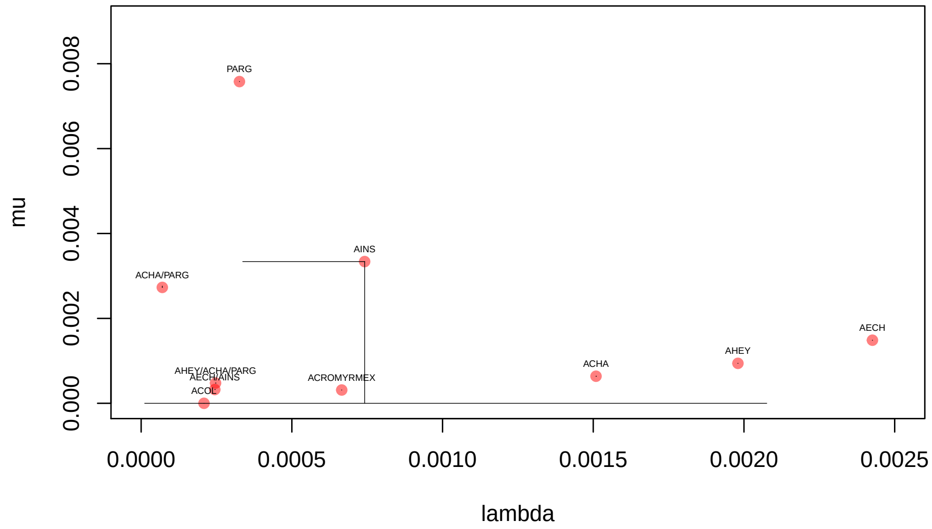

Supplementary Figure 24: λ and μ estimates generated from 2-parameter models for all ten analysed branches. Red dots show λ and μ estimates from best scoring models for each branch. Black bars show range of estimates across all replicates. Only branches AINS and ACOL vary considerably across runs.

```
load -i
↪ /usr/local/home/lschrader/data/inqGen18/geneFamilies/results/mcl.clean/largeQC.TEpso.nofilter.MCL.I01.5.cafe.filter100.tsv
↪ -t 20 -l /usr/local/home/lschrader/data/inqGen18/geneFamilies/CAFE/RUN5/RUN5.txt -p 0.05 -filter

#assign error models

errormodel -model cafe_errormodel_0.0526635742187.txt -sp AECH
errormodel -model cafe_errormodel_0.0478759765625.txt -sp AHEY
errormodel -model cafe_errormodel_0.0430883789063.txt -sp AINS
errormodel -model cafe_errormodel_0.0287255859375.txt -sp ACHA
errormodel -model cafe_errormodel_0.0.txt -sp ACOL
errormodel -model cafe_errormodel_0.129265136719.txt -sp PARG

#(((AHEY,(ACHA,PARG)),(AECH,AINS)),ACOL);
#calculate lambdamu
lambdamu -s -t (((1,(1,1)1)1,(1,1)1)1,1);
report null
lambdamu -s -t (((2,(1,1)1)1,(1,1)1)1,1);
lambdamu -s -t (((2,(1,1)1)1,(1,1)1)1,1);
```

```
lambdamu -s -t (((2,(1,1)1)1,(1,1)1)1,1);
lambdamu -s -t (((2,(1,1)1)1,(1,1)1)1,1);
lambdamu -s -t (((2,(1,1)1)1,(1,1)1)1,1);
lambdamu -s -t (((2,(1,1)1)1,(1,1)1)1,1);
lambdamu -s -t (((2,(1,1)1)1,(1,1)1)1,1);
lambdamu -s -t (((2,(1,1)1)1,(1,1)1)1,1);
```

The best-scoring λ and μ parameter estimates were clustered by Hartigan-Wong k-means clustering ($k = 2$ to $k = 8$), choosing each time 1000 random sets as initial centers (nstart=1000). Clustering results are shown in Supplementary Table 41 and Supplementary Figure 25.

Supplementary Table 41: Kmeans clustering (k=2 to k=8) of λ and μ parameter estimates from best-scoring Maximum Likelihood modelling runs for all ten branches.

Node	k2	k3	k4	k5	k6	k7	k8
**ACHA**	2	2	1	1	6	3	3
**ACHA/PARG**	2	1	4	4	5	1	4
**ACOL**	2	2	2	3	2	6	2
**ACRO**	2	2	2	3	2	6	5
**AECH**	2	2	1	5	3	5	8
**AECH/AINS**	2	2	2	3	2	6	2
**AHEY**	2	2	1	1	6	2	7
**AHEY/ACHA/PARG**	2	2	2	3	2	6	2
**AINS**	2	1	4	4	1	7	6
**PARG**	1	3	3	2	4	4	1

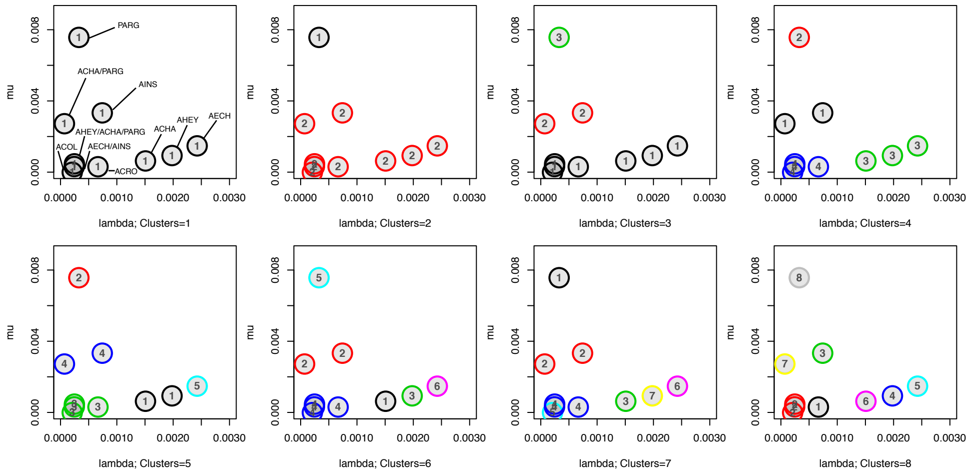

Supplementary Figure 25: Clustering of λ and μ estimates generated from 2-parameter models for all ten analysed branches. Plots show clustering results for k=2 to k=8 clusters. Coloring of dots indicates membership to the same cluster in each plot.

An analysis of λ and μ estimates by partitioning around medoids (using the `pamk` function in R (library `fpc`)), suggested an optimal number of four clusters (see Supplementary Table 42). Hierachical clustering based on euclidean distances of λ and μ estimates suggested between four and five clusters (Fig. 26).

Supplementary Table 42: Optimal clustering according to `pamk`.

ClusterID	**Members**
**Cluster1**	ACOL,ACRO,AECH/AINS
**Cluster2**	ACHA,AECH,AHEY
**Cluster3**	ACHA/PARG,AINS
**Cluster4**	PARG

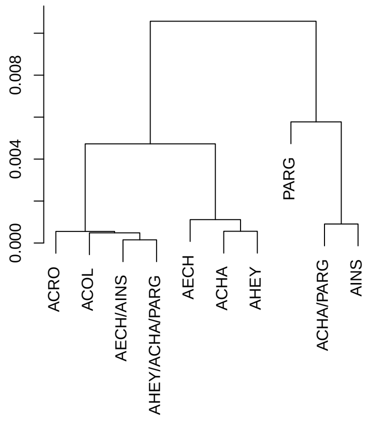

Supplementary Figure 26: Euclidean distance-based hierachical clustering of λ and μ estimates generated from 2-parameter models for all ten analysed branches.

We continued with running CAFE on higher-parameter models, with either four, five, six, or seven independent λ and μ estimates or on the full models with independent estimates for λ and μ for all ten branches. Since the full model did not converge properly and produced likelihood scores significantly worse than less parameterized models, we continued with lower parameter models. In total, we ran over 200 different models, rerunning each model at least eight times each to ensure convergent and robust likelihood scores, and λ and μ estimates. Maximum likelihood scores were compared for nested models using likelihood ratio tests in R (function `lr.test()` in the library `library("extRemes")`) to identify best fitting model(s). Supplementary Table 43 provides a summary of all the different models run in CAFE. The best fit to the data was a model with six discrete λ and μ (`(((2,(3,6)4)5,(1,4)5)5,5)`, maximum likelihood score = 26346.4, equivalent to the six clusters inferred using k-means clustering; see Supplementary Figure 25). The second best model was a five parameter model with a largely identical clustering (`(((3,(3,1)5)2,(4,5)2)2,2)`, maximum likelihood score = 26365.1).
Figure 27 shows the final inferred rates λ and μ for the best scoring model with six clusters. Note that the two nodes defining the origins of social parasites (Nodes 2 and 9) cluster together and show an increased frequency of gene losses. Similarly, node 10 (Parg) shows the highest frequency of gene losses but a very rate of gene gains.

All non-2-parameter CAFE models were run using the following script structure:

```
tree (((AHEY:0024.983,(ACHA:0016.335,PARG:0016.335):0008.648):0027.567,
↪  (AECH:0009.605,AINS:0009.605):0042.945):0065.115,ACOL:0117.665);
load -i /usr/local/home/lschrader/data/inqGen18/geneFamilies/results/mcl.clean/
↪  largeQC.TEpso.nofilter.MCL.I01.5.cafe.filter100.tsv -t 20 -l
↪  /usr/local/home/lschrader/data/inqGen18/geneFamilies/CAFE/RUN4C/RUN4C.txt -p 0.05 -filter

#assign error models
errormodel -model cafe_errormodel_0.0526635742187.txt -sp AECH
errormodel -model cafe_errormodel_0.0478759765625.txt -sp AHEY
errormodel -model cafe_errormodel_0.0430883789063.txt -sp AINS
errormodel -model cafe_errormodel_0.0287255859375.txt -sp ACHA
errormodel -model cafe_errormodel_0.0.txt -sp ACOL
errormodel -model cafe_errormodel_0.129265136719.txt -sp PARG

#(((AHEY,(ACHA,PARG)),(AECH,AINS)),ACOL);
#calculate lambdamu
lambdamu -s -t (((2,(3,6)4)5,(1,4)5)5,5);
report m6a
lambdamu -s -t (((2,(3,6)4)5,(1,4)5)5,5);
report m6b
lambdamu -s -t (((2,(3,6)4)5,(1,4)5)5,5);
report m6c
lambdamu -s -t (((2,(3,6)4)5,(1,4)5)5,5);
report m6d
lambdamu -s -t (((2,(3,6)4)5,(1,4)5)5,5);
report m6e
lambdamu -s -t (((2,(3,6)4)5,(1,4)5)5,5);
report m6f
lambdamu -s -t (((2,(3,6)4)5,(1,4)5)5,5);
report m6g
lambdamu -s -t (((2,(3,6)4)5,(1,4)5)5,5);
report m6h
lambdamu -s -t (((2,(3,6)4)5,(1,4)5)5,5);
report m6i
```

To ensure that the analysis of gene family size evolution was not biased by incomplete gene annotations (see above), particularly in *P. argentina*, we repeated the analysis using strictly homology-based annotations produced with GeMoMa v1.7.1. We used protein-coding gene annotations of the following 10 myrmicine genomes available in NCBI as reference for the annotation of *At. colombica* (Acol), *A. echinatior* (Aech), *A. insinuator* (Ains),*A. heyeri* (Ahey), *P. argentina* (Parg), and *A. charruanus* (Acha):

```
GCF_000187915.1 Pogonomyrmex barbatus
GCF_000188075.2 Solenopsis invicta
GCF_000949405.1 Vollenhovia emeryi
GCF_000956235.1 Wasmannia auropunctata
GCF_001594055.1 Trachymyrmex zeteki
GCF_001594065.1 Cyphomyrmex costatus
GCF_001594075.1 Trachymyrmex cornetzi
```

Supplementary Table 43: Overview of all models run in `CAFE` to infer gene family evolutionary rates in *Acromyrmex* ants.

model name	cluster tree
null model	(((1,(1,1)1)1,(1,1)1)1,1)
2-par. model	(((1,(1,1)1)1,(1,1)1)1,2)
2-par. model	(((1,(1,1)1)1,(1,1)1)2,1)
2-par. model	(((1,(1,1)1)1,(1,1)2)1,1)
2-par. model	(((1,(1,1)1)1,(1,2)1)1,1)
2-par. model	(((1,(1,1)1)1,(2,1)1)1,1)
2-par. model	(((1,(1,1)1)2,(1,1)1)1,1)
2-par. model	(((1,(1,1)2)1,(1,1)1)1,1)
2-par. model	(((1,(1,2)1)1,(1,1)1)1,1)
2-par. model	(((1,(2,1)1)1,(1,1)1)1,1)
2-par. model	(((2,(1,1)1)1,(1,1)1)1,1)
4-par. model	(((1,(1,3)4)2,(1,4)2)2,2)
4-par. model	(((4,(4,2)3)1,(4,3)1)1,1)
5-par. model	(((3,(3,1)4)2,(3,5)2)2,2)
5-par. model	(((3,(3,1)5)2,(3,5)2)4,2)
5-par. model	(((3,(3,1)5)2,(4,5)2)2,2)
5-par. model	(((3,(4,1)5)2,(3,5)2)2,2)
6-par. model	(((2,(3,6)4)5,(1,4)5)5,5)
6-par. model	(((6,(6,4)5)2,(3,1)2)2,2)
7-par. model	(((2,(3,4)1)6,(5,7)6)6,6)
full model	(((1,(2,3)4)5,(6,7)8)9,10)

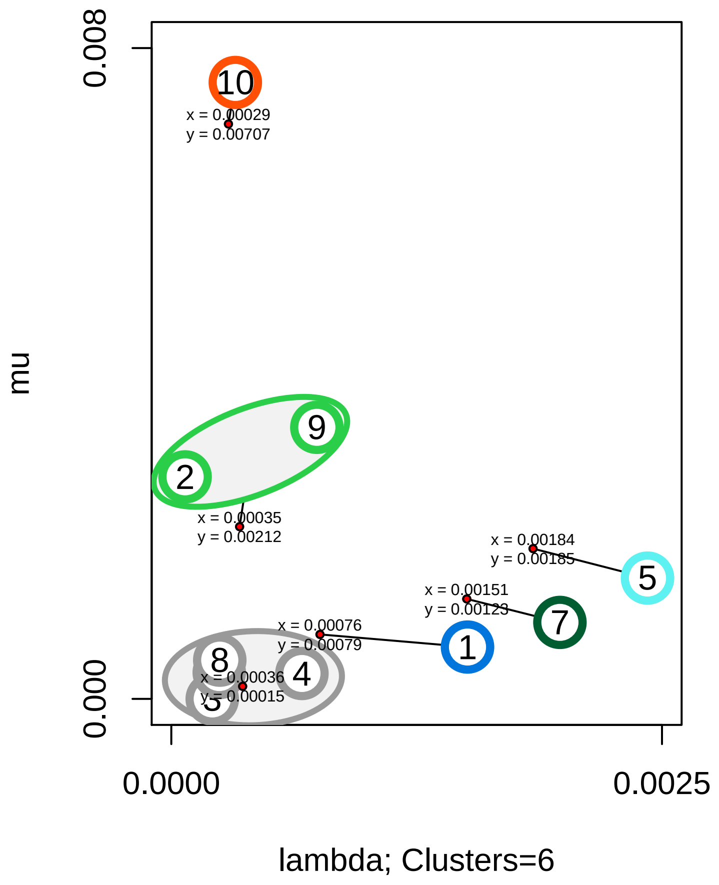

Supplementary Figure 27: Best fitting model with six clusters of λ and μ estimates. λ and μ estimates for each cluster are indicated with small red dots and "x=" and "y=" notations. λ and μ estimates for individual branches inferred from two-parameter models are indicated by colored and numbered dots. Coloring of dots indicates membership to the same cluster in each plot. 1=Acha, 2=Acha/Parg, 3=Acol, 4=Acromyrmex, 5=Aech, 6=Aech/Ains, 7=Ahey, 8=Ahey/Acha/Parg, 9=Ains,10=Parg.

`GCF_001594115.1 Trachymyrmex septentrionalis`

```
GCF_003070985.1 Temnothorax curvispinosus
GCF_003260585.2 Monomorium pharaonis
```

The strictly homology-based annotations produced very high BUSCO scores (run in `protein` mode against `hymenotpera_odb9`) for all species, ranging between 95.8% (Ahey) to 97.8% (Acha and Ains).

```
ACHA: C:97.8%[S:97.3%,D:0.5%],F:1.0%,M:1.2%,n:4415
AECH: C:97.5%[S:97.0%,D:0.5%],F:1.2%,M:1.3%,n:4415
AHEY: C:95.8%[S:95.3%,D:0.5%],F:2.4%,M:1.8%,n:4415
PARG: C:97.2%[S:96.8%,D:0.4%],F:1.4%,M:1.4%,n:4415
AINS: C:97.8%[S:97.3%,D:0.5%],F:1.0%,M:1.2%,n:4415
ACOL: C:97.5%[S:97.3%,D:0.2%],F:1.2%,M:1.3%,n:4415
```

Based on these annotations, we subsequently used the same approach as described above to filter and cluster protein coding gene sets and analyze gene family size evolution. As expected from higher BUSCO scores, CAFE error estimates for these annotations were lower than those calculated for the original annotations:

```
PARG: 0.022
ACOL: 0.000
AINS: 0.008
AHEY: 0.010
ACHA: 0.007
AECH: 0.014
```

Two-parameter models calculated with CAFE (see Supplementary Figure 28) largely agreed with the findings from the initial analysis, in particular in regard to the very high rates of gene loss in *P. argentina*, confirming that CAFE properly accounts for incomplete annotations with error modelling. Note however, that gene loss estimates for *A. insinuator* were lower in this second iteration of the analysis.

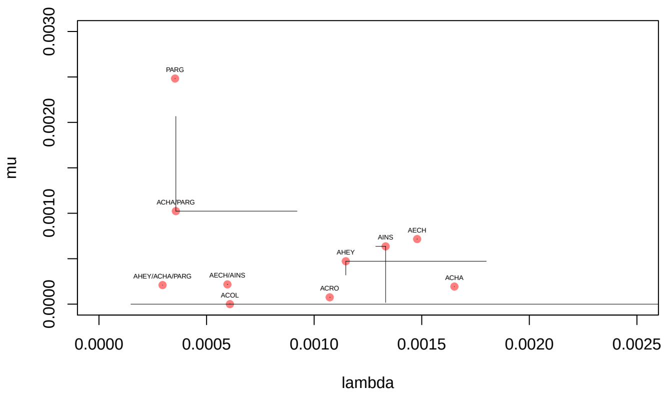

Supplementary Figure 28: λ and μ estimates generated from 2-parameter models using homology-only gene annotations. Red dots show λ and μ estimates from best scoring models for each branch. Black bars show range of estimates across all replicates (n=234). Four branches (AINS, ACHA/PARG, AHEY and ACOL) vary considerably across runs.

16 Gene Ontology term enrichment of gene families

We tested for significant enrichment of Gene Ontology (GO) terms in gene families that underwent significant size changes in leaf-cutting ants with `topGO`. For this, we retrieved GO annotations for each gene cluster from the individual proteins belonging to a given cluster. We tested for GO enrichment across all gene families that are smaller in social parasites than in the respective host species (AINS $<$ AECH, PARG $<$ AHEY & ACHA $<$ AHEY, see Supplementary Table 44 for results with parent-child tests not correcting for multiple testing and Supplementary Table 45 for Fisher tests ("classic" algorithm in `TopGO`) with FDR correction). We furthermore tested for gene families that are smaller in hosts than in their respective parasite species (AINS $>$ AECH, PARG $>$ AHEY & ACHA $>$ AHEY), which did not reveal any GO terms to be enriched, with or without correcting for multiple testing. In both analyses, we used all identified clusters as background set to test for GO enrichment.

Supplementary Table 44: Gene Ontology term enrichment ($p < 0.05$) of gene families consistently smaller in inquiline parasites than in their respective hosts. `GOSOURCEDATE`: 2020-05-02.

GO.ID	Term	Annotated	Significant	Expected	parentChild
**GO:0005549**	odorant binding	42	7	0.37	1.50E-08
**GO:0060089**	molecular transducer activity	127	8	1.12	1.00E-05
**GO:0004984**	olfactory receptor activity	31	7	0.27	0.00032
**GO:0046906**	tetrapyrrole binding	30	3	0.26	0.00129
**GO:0003824**	catalytic activity	1497	21	13.19	0.00634
**GO:0072341**	modified amino acid binding	1	1	0.01	0.00782
**GO:0031177**	phosphopantetheine binding	1	1	0.01	0.01064
**GO:0016491**	oxidoreductase activity	249	8	2.19	0.01473
**GO:0048037**	cofactor binding	127	4	1.12	0.01551
**GO:0034061**	DNA polymerase activity	14	2	0.12	0.0311
**GO:0005506**	iron ion binding	34	2	0.3	0.0343
**GO:0016787**	hydrolase activity	541	12	4.77	0.03913
**GO:0020037**	heme binding	30	3	0.26	0.04076
**GO:0004527**	exonuclease activity	19	3	0.17	0.04385
**GO:0032501**	multicellular organismal process	89	8	1.05	5.60E-06
**GO:0006260**	DNA replication	34	2	0.4	0.0032
**GO:0046189**	phenol-containing compound biosynthetic ...	1	1	0.01	0.0043
**GO:0018958**	phenol-containing compound metabolic pro...	1	1	0.01	0.0052
**GO:0032324**	molybdopterin cofactor biosynthetic proc...	2	1	0.02	0.0059
**GO:0003008**	system process	49	8	0.58	0.0064
**GO:0044550**	secondary metabolite biosynthetic proces...	1	1	0.01	0.0066
**GO:0019748**	secondary metabolic process	1	1	0.01	0.0112
**GO:0055114**	oxidation-reduction process	197	6	2.33	0.0178
**GO:0006508**	proteolysis	173	5	2.04	0.0181
**GO:0051189**	prosthetic group metabolic process	5	1	0.06	0.0267
**GO:0043545**	molybdopterin cofactor metabolic process	5	1	0.06	0.0335
**GO:1901617**	organic hydroxy compound biosynthetic pr...	8	1	0.09	0.043
**GO:0042440**	pigment metabolic process	4	1	0.05	0.0442
**GO:0006979**	response to oxidative stress	5	1	0.06	0.0459
**GO:0016020**	membrane	706	15	7.18	7.30E-05
**GO:0005839**	proteasome core complex	8	1	0.08	0.019
**GO:0000502**	proteasome complex	13	1	0.13	0.026

17 Odorant receptor gene annotation

Odorant receptors (OR) were annotated in all available attine genomes. We initially retrieved manually curated OR annotations for *A. echinatior*, *At. cephalotes*, and *Solenopsis invicta* published by McKenzie et al.[13]. Gene annotations for *A. echinatior* were again manually refined using unpublished *A. echinatior* antennal RNAseq data (pers. comm. Bitao Qiu). Most of these manual refinements were

Supplementary Table 45: Gene Ontology term enrichment after correcting for multiple testing ($FDR < 0.05$) of gene families consistently smaller in inquiline parasites than in their respective hosts. GOSOURCEDATE: 2020-05-02.

GO.ID	Term	Annotated	Significant	Expected	classic	FDR
**GO:0004984**	olfactory receptor activity	31	7	0.27	5.00E-09	5.56E-06
**GO:0005549**	odorant binding	42	7	0.37	4.80E-08	2.67E-05
**GO:0004888**	transmembrane signaling receptor activit...	118	8	1.04	5.90E-06	0.00218693
**GO:0038023**	signaling receptor activity	127	8	1.12	1.00E-05	0.002224
**GO:0060089**	molecular transducer activity	127	8	1.12	1.00E-05	0.002224
**GO:0007606**	sensory perception of chemical stimulus	45	8	0.53	2.40E-08	5.10E-05
**GO:0007600**	sensory perception	47	8	0.55	3.50E-08	7.43E-05
**GO:0050877**	nervous system process	47	8	0.55	3.50E-08	7.43E-05
**GO:0007608**	sensory perception of smell	31	7	0.37	3.50E-08	7.43E-05
**GO:0003008**	system process	49	8	0.58	4.90E-08	0.00010388
**GO:0032501**	multicellular organismal process	89	8	1.05	5.60E-06	0.0118664

adding short first exons that were intentionally left out in the original curated gene set (McKenzie pers. comm.). We used these manually curated annotations to manually annotate OR genes in *A. insinuator*.

Based on the manual curated gene sets from *A. echinatior*, *A. insinuator*, and *At. cephalotes*, we annotated OR genes in genomes using a gene annotation pipeline that combines three different lines of evidence.

- We adopted a pipeline based on a combination of `tblastn` and the gene prediction software `exonerate v2.2.0`, developed by Zhou et al.[14]: First, potential OR loci are identified using `tblastn` alignments of OR reference protein sequences against the target genome. Then, gene models are developed with `exonerate` for each locus using reference ORs with homology to a given locus.

- We used `GeMoMa v1.5.2` to predict OR gene models. GeMoMa is a homology-based gene prediction program that uses amino acid sequence and intron position conservation of a reference genome (using a gff and fasta file of the reference genome) to annotate protein-coding genes in a target genome.

  For the GeMoMa annotation, each target genome was first annotated independently with each of the reference genomes using the following parameters:

```
GeMoMa CLI Extractor: f=false r=true Ambiguity=AMBIGUOUS

tblastn -query cds-parts.fasta -db blastdb -evalue 100.0 -out tblastn.txt -outfmt "6 std sallseqid
↪  score nident positive gaps ppos qframe sframe qseq sseq qlen slen salltitles" -db_gencode 1
↪  -matrix BLOSUM62 -seg no -word_size 3 -comp_based_stats F -gapopen 11 -gapextend 1
↪  -num_threads 20

GeMoMa CLI GeMoMa t=tblastn.txt c=cds-parts.fasta a=assignment.tabular tg=target  outdir=. p=60
↪  ct=0.1 rt=0.1 m=20000
```

  `GeMoMa` predictions for each species were extracted with `GeMoMa GAF`

```
GeMoMa CLI GAF g=predicted_annotation.gff r=-10000 c=F
```

  Further, OR predictions were filtered and combined with `GeMoMa GAF` using three different settings.

```
GeMoMa CLI GAF g=../q1/predicted_annotation.gff g=../q2/predicted_annotation.gff
↪  g=../q3/predicted_annotation.gff c=false r=0 m=true cbf=0

GeMoMa CLI GAF g=../q1/predicted_annotation.gff g=../q2/predicted_annotation.gff
↪  g=../q3/predicted_annotation.gff c=true

GeMoMa CLI GAF g=../q1/predicted_annotation.gff g=../q2/predicted_annotation.gff
↪  g=../q3/predicted_annotation.gff r=0 c=false
```

- We used `tblastx` and blasted all reference OR exons against the target genome and generated gffs of OR-exon homologous regions.

```
tblastx -db genome.fa -query ORexons.fa -out ORexons.bls -evalue 1e-2 -outfmt 6
```

We combined results from the different gene/exon predictions using `Evidence Modeler v1.1.1`, using the following settings:

```
partition_EVM_inputs.pl --genome genomeFa \
     --gene_predictions gemoma.prot.gff \
     --protein_alignments blast.exonerate.gff \
     --segmentSize 100000 --overlapSize 40000 --partition_listing partitions_list.out

write_EVM_commands.pl --genome $genomeFa --weights $base/$species/EVM/weights.txt \
      --gene_predictions gemoma.prot.gff \
      --protein_alignments blast.exonerate.gff \
      --search_long_introns 1000 \
      --re_search_intergenic 4000 \
      --output_file_name evm.out  --partitions partitions_list.out >  commands.list

 convert_EVM_outputs_to_GFF3.pl  --partitions partitions_list.out --output evm.out  --genome $genomeFa
 gff3_file_to_proteins.pl  $f $genomeFa > $f.prot.fa
```

We subsequently added all gene models predicted by `GeMoMa` for reference 1 (*A.echinatior*) not overlapping models included in the final set produced by `Evidence Modeler`. These models largely comprise incomplete gene models without canonical start and/or stop-codons. We divided predicted genes into those coding and those not coding for a complete ”7tm_6” (PF02949) pfam domain, which is characteristic for insect ORs.

```
 pfam_scan.pl -fasta fullORannotation.fa -dir ~/data/pfam/ -outfile fullORannotation.domains.out
```

Finally, we used `blastp` to identify fragmented and incomplete predictions that show significant homology to reference ORs among the set of predicted models lacking the ”7tm_6” domain.

```
makeblastdb -in referenceORfa -dbtype prot
nice blastp -db referenceORfa -query OR.no7tm6.fa -out OR.no7tm6.bls -evalue 1e-5 -outfmt 6
```

For each species, the final set of predicted ORs comprises models coding for an intact 7tm_6 domain and models with significant similarity to known ORs. These models were finally renamed using the following conventions.

- NTE = missing start, i.e. missing N-terminus.
- CTE = missing stop, i.e. missing C-terminus.
- NC = missing start and stop, i.e. missing N- and C-terminus.
- NC = missing start and stop, i.e. missing N- and C-terminus.
- fd = domain putatively incomplete at n-terminus.
- df = domain putatively incomplete at c-terminus.
- fdf = domain putatively incomplete at n- and c-terminus.
- dH = domain missing, but protein homologous to other ORs (with blastp $e - value < 1e - 10$).

Table 46 summarizes the number of gene models annotated in each species.

Supplementary Table 46: Summary of odorant receptor genes annotated in attine ants.

Species	**Total**	**incompl.**	**N/C-term. missing**	**NC**	**NTE**	**CTE**	**dH**	**domain put. incomplete**	**complete**
**Acep**	568	173	139	32	55	52	114	113	454
**Acha**	501	101	85	37	18	30	72	75	429
**Acol**	589	138	106	50	24	32	117	72	472
**Aech**	594	178	163	100	22	41	138	81	456
**Ahey**	648	248	225	114	35	76	181	158	467
**Ains**	489	78	66	33	14	19	65	52	424
**Ccos**	468	72	66	28	15	23	53	52	415
**Parg**	336	93	69	33	17	19	73	73	263
**Pcor**	568	149	136	76	25	35	111	70	457
**Tsep**	517	127	113	64	13	36	106	60	411
**Mzet**	481	114	94	32	25	37	86	50	395

In order to confirm gene losses in the social parasites independent of gene annotations, we aligned OR protein sequences annotated in *At. colombica* against the raw unfiltered genome assemblies of *P. argentina*, *A. charruanus*, *A. heyeri* and *A. insinuator* as well as the published assembly of *A. echinatior* with `mmseqs2` (version `de06950ff733478fc87195f9c57683c7dd7207e8`). HSPs over 200 bp were extracted and merged into discrete OR loci using `bedtools merge` (with `-d 1500`). This approximate, but annotation-independent approach confirmed the reduction of the OR repertoires in the in social parasites, yielding 523 loci in *A. echinatior*, 572 in *A. heyeri*, 484 loci in *A. insinuator*, 502 loci in *A. charruanus*, and 316 loci in *P. argentina*.

18 Phylogenetic reconstruction of attine odorant receptor genes

We reconstructed the OR gene phylogeny for eleven attine species using amino acid translations of all predicted genes. First, we assigned proteins to subfamilies by blasting protein sequences against a gene family assignment database (McKenzie pers. comm.) built from previously reconstructed ant OR phylogenies[15]. We assigned ORs to subfamilies according to the best hit against the assignment

database. We created subfamily-wide alignments using `linsi v7.307` and subsequently merged all subfamily alignments using `mafft v7.307`. The phylogenetic tree was computed using `FastTreeMP v2.1.10`. The tree was rooted in `FigTree v1.4.3`, setting the clade containing Orco (odorant receptor co-receptor) as outgroup to all other ORs. We corrected blast-based subfamily assignments for two genes, following phylogenetic inference of subfamily membership. AheyOr-372-NTEfd was reassigned from subfamily H to E, and AchaOr-402-NCfd was reassigned from subfamily 9E to G.

Clades were defined using the `R` function `getCladesofSize()` (`clade.size` = 5) from the `phytools` library. We defined 321 clades (median size = 11) belonging to 27 different subfamilies (Fig. 29). We identified 80 clades containing fewer paralogs in *P. argentina* than in *A. heyeri*. Five clades contained fewer genes in both *P. argentina* and *A. charruanus* than in *A. heyeri*. In all 85 clades, *A. heyeri* did not contain fewer paralogs than *A. echinatior*, suggesting gene loss in the parasites as the cause of the gene count differences within these clades. Ten clades contained fewer genes in *P. argentina* and *A. insinuator* and six clades showed fewer paralogs in all three parasite species compared to their hosts. Figure 34 shows the phylogenetic tree computed for OR genes across the eleven attine species. Supplementary Table 47 lists all subfamilies and the respective gene counts in the different species. The table also contains information about the number of complete and fragmented gene models. Supplementary Table 48 provides a more detailed summary of the annotations. Supplementary Figure 35 summarized OR subfamily sizes across the different species.

Supplementary Table 47: Overview of odorant receptor genes assigned to subfamilies and their annotation quality in attine ants. Fragmented here means that a gene model lacks a C-terminus, an N-terminus or that the domain is incomplete.

subfamily	**Acep**	**Acha**	**Acol**	**Aech**	**Ahey**	**Ains**	**Ccos**	**Parg**	**Pcor**	**Tsep**	**Mzet**
**9E.complete**	105	117	147	121	89	124	82	40	114	109	105
**9E.dH**	23	3	10	5	14	1	5	5	5	5	7
**9E.fragment**	43	24	29	28	54	16	17	22	13	13	12
**A.complete**	3	6	3	4	5	5	12	0	8	6	7
**A.dH**	5	4	5	6	5	6	1	0	1	4	4
**A.fragment**	1	0	1	3	0	2	1	0	1	1	0
**B.complete**	1	1	1	1	1	1	1	1	2	1	0
**B.fragment**	0	0	0	0	0	0	0	0	0	0	1
**C.complete**	1	1	1	1	1	1	0	1	1	0	1
**C.dH**	0	0	0	0	0	0	1	0	0	0	0
**C.fragment**	0	0	0	0	0	0	1	0	0	2	0
**D.complete**	3	4	4	4	4	4	4	3	4	4	4
**D.fragment**	1	0	0	0	0	0	0	0	0	0	0
**E.complete**	31	35	35	34	26	34	40	23	40	35	35
**E.dH**	3	2	2	1	2	1	0	1	2	3	3
**E.fragment**	8	4	5	5	13	5	0	5	5	5	3
**F.complete**	5	5	5	5	6	4	6	3	8	8	6
**F.dH**	0	0	0	0	0	1	0	1	0	0	0
**F.fragment**	1	1	1	1	1	1	0	0	0	1	0
**G.fragment**	1	2	1	1	2	1	1	1	1	1	0
**H.complete**	11	11	10	13	7	12	14	9	11	11	11
**H.dH**	0	1	1	1	4	1	0	2	1	1	1
**H.fragment**	5	4	5	2	5	3	2	3	5	4	4
**I.fragment**	1	1	1	1	1	1	1	1	1	1	1
**J.complete**	2	2	2	2	2	2	2	1	2	2	1
**J.fragment**	0	0	0	0	0	0	0	1	0	0	1
**K.complete**	2	1	2	2	2	2	2	2	2	2	2
**K.fragment**	0	1	0	0	0	0	0	1	0	1	0
**L.complete**	61	61	72	66	59	61	63	42	65	58	54
**L.dH**	0	3	2	1	4	0	0	2	1	1	1
**L.fragment**	14	14	7	6	21	7	6	6	8	7	10
**M.complete**	4	3	5	4	5	5	6	3	5	5	5
**M.dH**	0	1	0	0	0	0	0	0	0	0	0
**M.fragment**	1	2	0	2	3	1	1	2	0	1	0
**N.complete**	6	5	6	6	4	6	4	3	5	6	7
**N.fragment**	1	2	1	1	5	1	2	3	2	1	1
**O.complete**	1	1	1	1	1	1	1	1	1	1	2
**Orco.complete**	1	1	1	1	1	1	1	1	1	1	1
**P.complete**	11	11	11	12	11	10	5	3	12	11	8
**P.dH**	0	1	0	0	1	0	1	0	1	0	0
**P.fragment**	0	1	0	3	1	2	1	4	0	1	0
**Q.complete**	0	0	0	0	0	0	0	0	0	1	0
**Q.dH**	0	0	0	0	0	0	1	0	0	0	0
**Q.fragment**	1	1	1	1	1	1	1	1	1	0	1
**R.complete**	2	3	3	3	3	3	2	0	3	3	1
**R.fragment**	2	0	0	0	0	0	0	1	0	0	1
**S.complete**	2	2	1	2	1	2	2	0	3	2	2
**S.fragment**	0	0	1	0	1	0	0	1	1	0	0
**T.complete**	5	6	6	7	4	7	9	4	7	7	7
**T.fragment**	1	1	1	0	3	0	0	2	0	0	0
**U.complete**	29	26	32	29	25	32	45	15	35	30	30
**U.dH**	1	0	0	0	0	1	0	0	0	0	0
**U.fragment**	12	8	5	8	11	3	6	7	6	4	5
**V.complete**	44	46	49	53	47	51	58	29	54	46	50
**V.dH**	1	0	0	0	1	0	3	1	1	1	2
**V.fragment**	9	3	3	4	6	5	4	8	3	5	1
**W.fragment**	1	1	1	1	1	1	1	1	1	1	1
**XA.dH**	0	0	0	0	0	1	1	0	0	0	0
**XA.fragment**	1	1	1	1	1	0	0	0	1	1	1
**Z.complete**	1	1	1	1	1	1	1	1	1	1	1

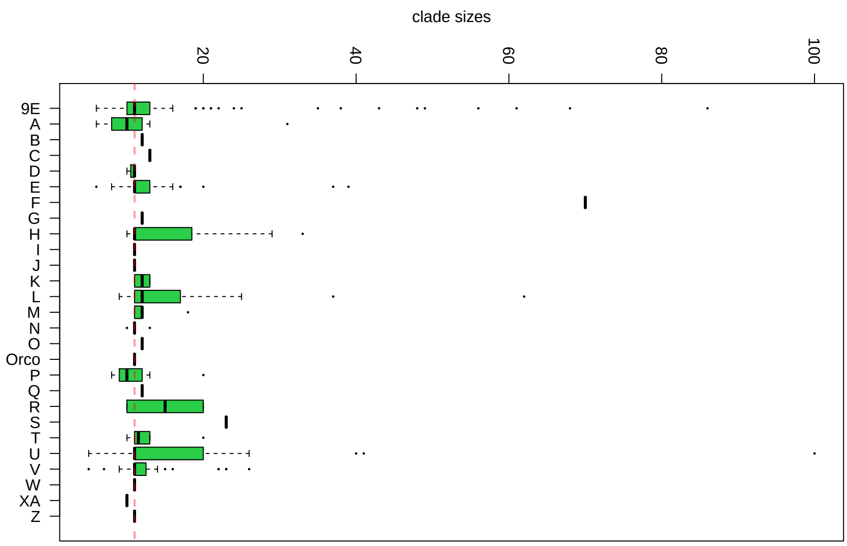

Supplementary Figure 29: Boxplots showing clade sizes across different subfamilies for odorant receptor genes in eleven attine species, with boxplot centers showing the median, hinges showing the first and third quantiles, and whiskers showing the 1.5 x inter-quantile range. The red dashed line indicates the overall median clade size (11).

We performed a hierachical clustering analysis (Fig. 30), comparing gene counts across different subfamilies in the eleven attine species. This analysis emphasized the dramatic change in *P. argentina* compared to the other leaf-cutting ant species. Furthermore, our analysis suggests that odorant receptor repertoires changed convergently in *A. insinuator* and *A. charruanus* as the two species cluster together. Similarly, the two host species *A. echinatior* and *A. heyeri* formed a cluster.

Gene tree reconciliation was done with `dlcpar 2.0.1` for each OR clade. For this, we retrieved CDS sequences for all genes of a given clade with `GenomeTools`' (v1.5.9) `extractfeat`. We trimmed CDS sequences to multiples of three while maintaining the ORF with `TransDecoder.LongOrfs`. CDS sequences were aligned with `prank v.150803` in `codon` mode and a phylogeny inferred with `FastTree -nt` . Gene trees were midpoint rooted with `gotree reroot v0.4.0`. For Clade285, we used outgroup rooting at OR `CcosOr-363`. We ran `dlcpar` in `search` mode and inferred tree-relations with `dlcoal v1.0`.

We further analysed patterns of OR evolution in the social parasites by running `HYPHY`'s `RELAX` test on OR clades. We only included complete gene models to avoid including pseudogenes and other gene fragments. We aligned CDS translated sequences of all genes of a given clade with `prank v.150803` and created CDS alignments with `pal2nal v.14`. We ran `RELAX` on terminal branches, defining leaf nodes of AINS, ACHA, and PARG as the test set and leaf nodes of ACOL, ACEP, AHEY, and AECH as the reference set. We did not find a significant deviation from zero for the distribution of $log_2(k)$. We further used `HyPhy`'s `absREL` algorithm to compute dN and dS individually for all branches. We calculated $dN/(dN+dS)$ for all terminal branches to compare evolutionary rates of ORs between leaf-

cutting species (LC; i.e. Acep, Acol, Aech, and Ahey), non-leaf-cutting species (NLC, i.e. Tsep, Mzet, Pcor, and Ccos), and social parasites (P; i.e. Acha, Parg, Ains). We found that average evolutionary rates are significantly inreased in parasites ("P") compared to the other groups. Differences between leaf-cutters and non-leaf-cutters were not significant (Fig. 33).

$log_2(k)$ was strongly negative for several OR clades, suggesting strong relaxation of selection in these clades (Fig. 32). In total, we identified 26 clades with highly negative $log_2(k)$ (9-exon: 11 clades, V: 5, U: 3, L: 2, P: 2, A: 1, Z: 1, N: 1). However, we found no evidence for relaxation ($k < 1$) or intensification ($k > 1$) of selection in the test set after correcting for multiple testing ($fdr < 0.05$). At $p < 0.1$, two clades (L-Clade-182, 9E-Clade-043) showed evidence for relaxed selection ($k < 1$) and five clades (9E-Clade-159, 9E-Clade-043, 9E-Clade-134, E-Clade-029, L-Clade-182, L-Clade-278, V-Clade-088) showed evidence for intensification of selection ($k > 1$) in the social parasite compared to the non-parasite species.

OGid	sf	klog2
9E-Clade-043	9E	-40.79245
9E-Clade-096	9E	-Inf
9E-Clade-137	9E	-42.37791
9E-Clade-160	9E	-69.00000
9E-Clade-165	9E	-Inf
9E-Clade-191	9E	-70.00000
9E-Clade-215	9E	-74.00000
9E-Clade-219	9E	-Inf
9E-Clade-284	9E	-46.19188
9E-Clade-296	9E	-35.97840
9E-Clade-311	9E	-22.80350
A-Clade-022	A	-40.07086
L-Clade-186	L	-40.21174
L-Clade-239	L	-37.94331
N-Clade-199	N	-Inf
P-Clade-193	P	-39.74895
P-Clade-223	P	-35.85716
U-Clade-110	U	-36.16397
U-Clade-113	U	-36.60044
U-Clade-244	U	-Inf
V-Clade-145	V	-Inf
V-Clade-150	V	-Inf
V-Clade-209	V	-Inf
V-Clade-225	V	-Inf
V-Clade-231	V	-Inf
Z-Clade-015	Z	-Inf

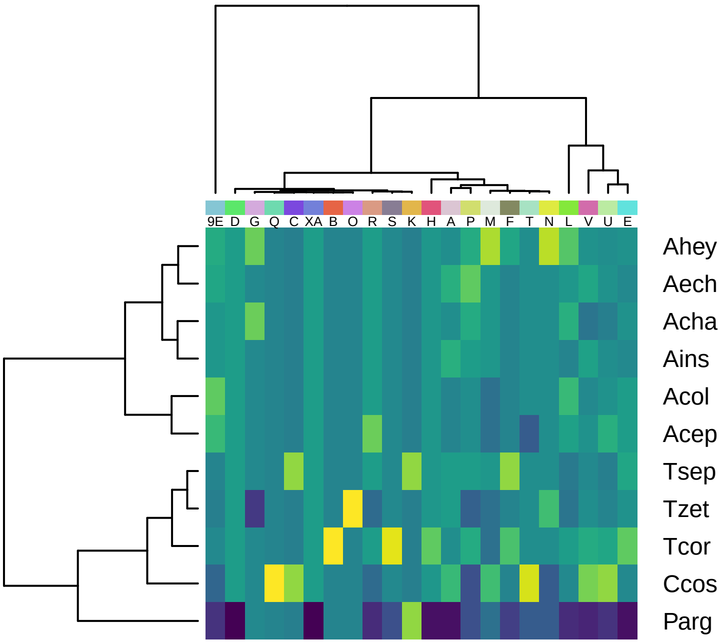

Supplementary Figure 30: Heatmap and hierachical cluster comparing OR subfamily sizes across eleven attine species. Heatmap scaled by column.

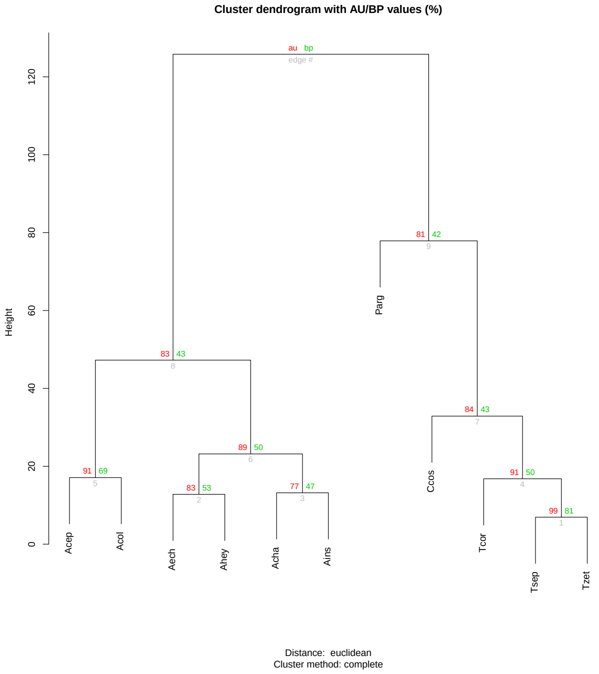

Supplementary Figure 31: Bootstrapped hiercachical cluster on OR subfamily sizes across eleven attine species. The split between Acha/Ains and Ahey/Aech is supported at 50% of the bootstrap replicates (n=10000) with an approximately unbiased (AU) p-value of 89%.

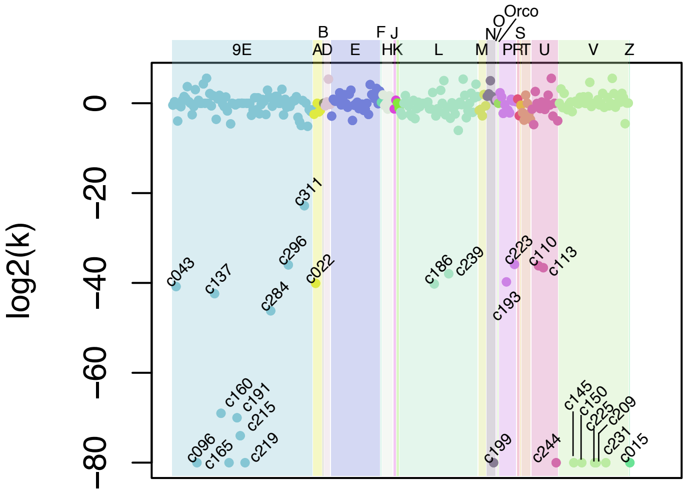

Supplementary Figure 32: Summary of the selection intensification/relaxation parameter k (as $log_2(k)$) for all analysed odorant receptor clades. Shown are $log_2(k)$ values for each clade, with clades labelled individually, if $log_2(k) < -20$. Clades with $k = 0$ where plotted here as $log_2(k) = -80$.

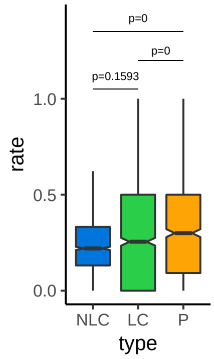

Supplementary Figure 33: Comparison of average evolutionary rates ($dN/(dN + dS)$) in terminal branches of the OR phylogeny. P = Social parasite species, LC = leaf-cutting ant species, NLC = non-leaf-cutting species. We tested for statistically significant differences in average evolutionary rates using bonferroni-corrected, two-sided `coin::wilcox_test` in `R` with 20000 samples. Boxplot centers show the median, hinges show the first and third quantiles, and whiskers show the 1.5 x inter-quantile range.

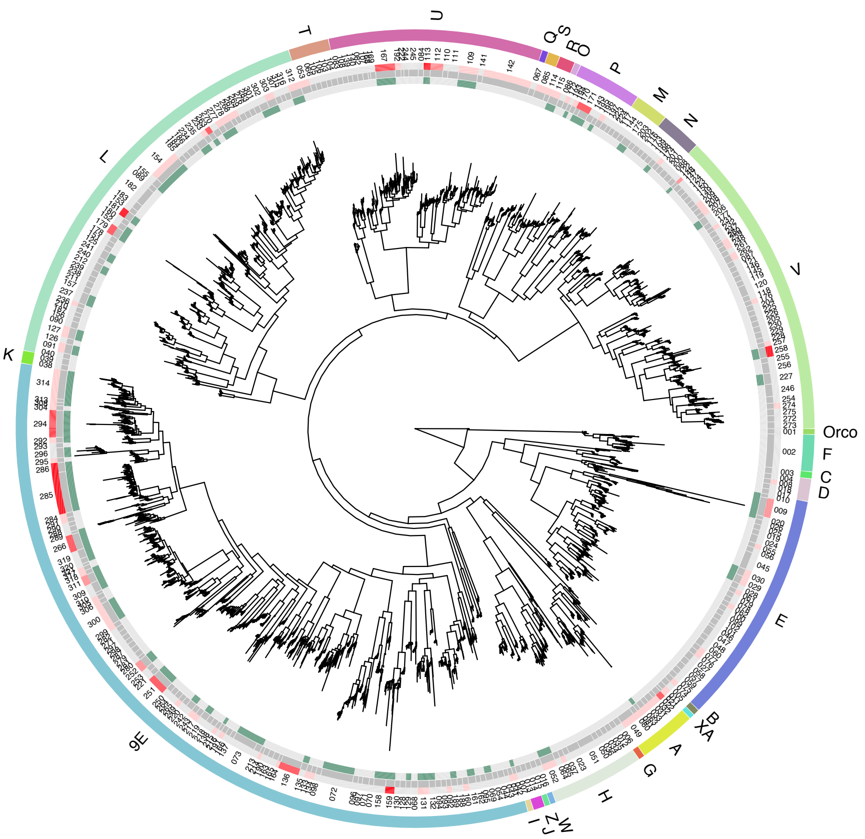

Supplementary Figure 34: Odorant receptor (OR) gene phylogeny for eleven attine species. The outermost circle shows subfamilies, the second circle shows in red those clades containing fewer OR genes in inquiline parasite species. The third circle show clades (corresponding numbers in black). The innermost circle shows in green those clades that have on average more paralogs in the leaf-cutting ant species than in *Trachymyrmex*, *Paratrachymyrmex*, *Mycetomoellerius* and *Cyphomyrmex* species.

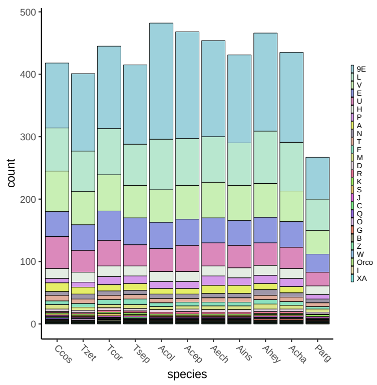

Supplementary Figure 35: Barplot summarizing OR gene subfamily sizes in different attine ant species.

Supplementary Table 48: Overview of odorant receptor gene models in attine ant genomes.

Species	# of genes	# of exons	# of introns	of CDS	Overlapping genes	Contained genes	Total gene length	Total exon length	Total intron length	Shortest gene	Shortest exon	Shortest intron	Shortest CDS	Longest gene	Longest mRNA	Longest exon	Longest intron	mean gene length	mean exon length	mean intron length	mean CDS length	mean exons per mRNA	mean introns per mRNA
Acep	568	3338	2770	568	12	6	2378414	550936	1833018	132	6	29	132	45775	45775	1061	28654	4187	165	662	970	6	5
Acha	501	2965	2464	501	0	0	1879925	511823	1373030	120	6	29	120	52496	52496	986	51926	3752	173	557	1022	6	5
Acol	589	3560	2971	589	0	0	2216534	587786	1634690	90	4	29	90	34526	34526	956	32276	3763	165	550	998	6	5
Aech	594	3143	2549	594	0	0	1940873	544480	1401491	45	6	32	45	53372	53372	986	52778	3267	173	550	917	5	4
Ahey	648	3197	2549	648	6	3	1981933	550728	1436303	51	3	29	51	49891	49891	1117	49321	3059	172	563	850	5	4
Ains	489	2929	2440	489	0	0	1951827	511870	1444837	150	6	29	147	55504	55504	986	54931	3991	175	592	1047	6	5
Ccos	468	2735	2267	468	0	0	2040786	491257	1554063	105	6	30	105	68814	68814	998	47371	4361	180	686	1050	6	5
Parg	336	1886	1550	336	2	1	1495320	320761	1177659	150	6	29	150	59439	59439	986	49318	4450	170	760	955	6	5
Pcor	568	3141	2573	568	0	0	1998180	547755	1455571	102	3	29	102	43950	43950	956	22209	3518	174	566	964	6	5
Tsep	517	2862	2345	517	2	1	1926972	498787	1432875	75	6	29	75	67972	67972	910	38915	3727	174	611	965	6	5
Mzet	481	2778	2297	481	0	0	1649171	480176	1173589	87	6	29	87	60372	60372	893	30481	3429	173	511	998	6	5

Supplementary Table 49: Overview of odorant receptor gene arrays.

species	genes contained	arrays	avg. gene count	avg. length
**Acep**	409	13	24	227 kb
**Acha**	353	11	27	173 kb
**Acol**	427	16	20.5	162 kb
**Aech**	339	13	21	136 kb
**Ahey**	355	13	22	143 kb
**Ains**	345	11	26	171 kb
**Parg**	215	9	20	129 kb

19 Olfactory receptor tandem array evolution

To gain a deeper understanding about the mutational events underlying the reduction in OR repertoires in social parasites, we identified OR gene tandem arrays in all annotated species of *Atta*, *Acromyrmex*, and *Pseudoatta*. We defined an OR tandem array as regions containing three or more OR genes with intergenic distances below 30 kb. We identified between 9 (*P. argentina*) and 16 (*At. colombica*) arrays that contain between 215 (*P. argentina*) and 427 genes (*At. colombica*) in total. The average number of genes contained in each array ranged from 20 in *P. argentina* to 27 in (*A. charruanus*) with an average length between 129 kb (*P. argentina*) to 227 kb (*At. cephalotes*). Supplementary Table 49 and Supplementary Figure 36 provide a detailed summary of these results. Our analysis shows that the social parasite species have retained the fewest arrays, with 9 in *P. argentina*, 11 in *A. insinuator* and *A. charruanus* compared to the other species of *Atta* and *Acromyrmex*. It is unclear whether the loss of arrays in the social parasites is a consequence of single mutational events deleting entire tandem arrays (particularly in *P. argentina*) or whether successive gene losses reduced OR tandem arrays (so they eventually contain fewer than 3 genes). The fact that the remaining arrays do however contain on average more genes in *A. charruanus* and in *A. insinuator* compared to non parasitic species could hint towards single mutational events removing entire arrays, as array degradation appears to not occur to the same extent in the remaining arrays.

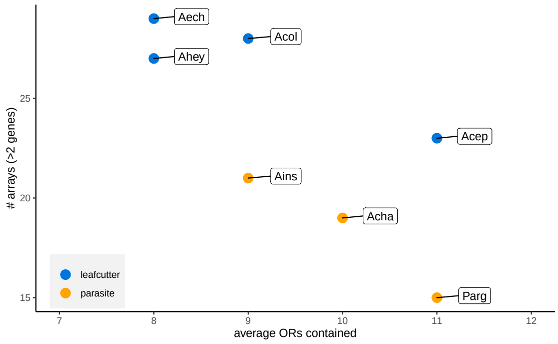

Supplementary Figure 36: OR gene tandem array diversity in leaf-cutting ants.

We used whole genome alignments (see below) to establish orthology between different tandem arrays, aiming to explore whether certain orthologous arrays are reduced in parallel across all parasite species. In general, we find that orthologous arrays are highly divergent across species, regardless of whether they are parasitic or non-parasitic (Fig. 37). We identified a single tandem array (array24) that showed parallel reductions in all three parasite species. Reductions in two of the three parasites were more common, but we also found cases where arrays were expanded in parasites relative to their hosts. Together, these findings suggest that OR tandem array evolution is a highly dynamic process, reflecting the evolutionary complexity of this gene family in ants.

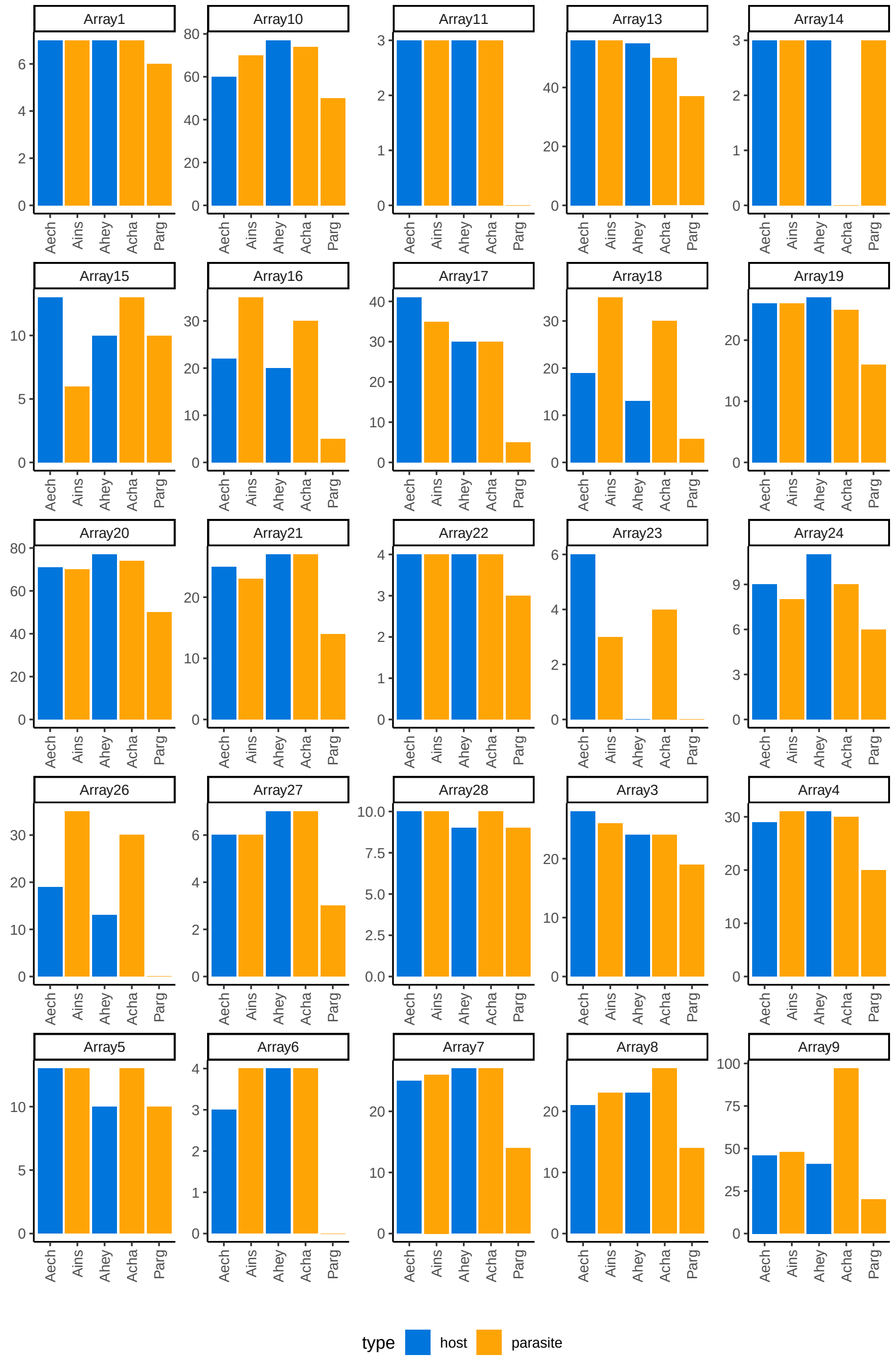

Supplementary Figure 37: Gene count diversity in orthologous OR gene arrays in *Acromymrex* hosts (blue) and parasites (orange).

20 X-ray Microtomography and Olfactory Lobe Volume Estimation

Ethanol-preserved samples of leaf-cutting ants were used for X-ray Microtomography (microCT) to visualize three-dimensional brain morphology and quantify olfactory lobe volume. Samples were stained in Lugol's solution (2.5% potassium iodide, 2.5% iodine) for 2–8 days and destained in 70% ethanol for 2–6 hours. Specimens were scanned wet in 70% ethanol, mounted inside a polyimide tube sealed on one side with hot glue and on the other with mounting putty. MicroCT was performed with a General Electric phoenix v—tome—x m x-ray computed tomography scanner (GE Sensing Inspection Technologies, Wunstorf, Germany), equipped with a 180 kv nanoCT tube at the Smithsonian Institution National Museum of Natural History. Scanning parameters are presented in Supplementary Table 50. Reconstruction of 3D images was completed with GE datos—x v 2.4.0.1199 and resulted in a stack of DICOM images with voxel sizes of 1.99–2.41 m. Segmentation and visualization of ant specimens was performed in Amira (FEI v5.5.0) and brain and olfactory lobe volumes were calculated using the MaterialStatistics module.

Supplementary Table 50: Overview of odorant receptor gene models in attine ant genomes.

Species	Specimen ID	Voltage (kV)	Current (mA)	Exposure (msec)	Number of Projections	Voxel Size (mm)	Magnif.
A. insinuator	LB28.1	70	180	1000	2700	2.1	95.2
A. echinatior	Ae529-215	60	230	1000	2600	2.0	100.6
P. argentina	CRL130225	80	120	500	1800	2.35	85.3
A. heyeri	CR131031-10	80	120	500	2700	2.3	87.1
A. charruanus	CR130225-03	80	120	500	2700	2.3	87.1

21 Evolution of the ”Major royal jelly protein/protein yellow” gene family

Major royal jelly protein (MRJP) and yellow-like genes were annotated manually in all leaf-cutting ant species. For this, we retrieved information for all genes from ant genomes annotated as ”Major royal jelly protein/protein yellow” in NCBI's databases using `eutils v.5.80`.

```
# retrieve all ant genes annotated as mrjps
esearch -db protein -query "(IPR017996 OR MRJP OR pf03022 OR (major AND royal AND jelly AND protein))
↪ AND txid34695[Organism:exp] AND refseq[filter]" | elink -target gene|efetch -format tabular >
↪ ant.mrjps.tsv

# get details about completeness of gene models and gene lengths
```

```
esearch -db protein -query "(IPR017996 OR MRJP OR pf03022 OR (major AND royal AND jelly AND protein))
↪  AND txid34695[Organism:exp] AND refseq[filter]" | efetch -format gb|egrep
↪  "\/gene=.*|COMPLETENESS.*|LOCUS.*|   ORGANISM.*" |tr "\n" "\t" |perl -pe 's/(gene\=.*?)\t/$1\n/g'
↪  |perl -pe 's/  +/\t/g'|cut -f 2,3,7,9|perl -pe "s/ aa//g"|perl -pe 's/\/gene=\"(.+?)\"/$1/g'>
↪  ant.mrjps.details.tsv

# select only complete  MRJPs between 300-750 aa
cat ant.mrjps.details.tsv|grep "full length"|awk '{if ($2<=500 && $2>=300) print $0}'|cut -f 4 >
↪  ant.mrjps.selection.lst
grep -f ant.mrjps.selection.lst ant.mrjps.tsv > ant.mrjps.selection.tsv
```

From these data, we retrieved gene annotation information and fasta sequences for all genes for each species. We used these ant-wide MRJP/yellow gene predictions to annotate each leaf-cutting ant genome with `GeMoMa v1.5.3` (`CLI GeMoMaPipeline threads=10 Extractor.p=T GAF.c=F GeMoMa.prefix=MRJP`). All predicted MRJPs/yellow genes were analysed with `signalP v4.1` to identify putative N-terminal signal peptides in the encoded proteins.

We generated between 25 to 37 gene models per species:

- *Acromyrmex charruanus* 33
- *Acromyrmex echinatior* 37
- *Acromyrmex heyeri* 31
- *Acromyrmex insinuator* 35
- *Pseudoatta argentina* 33
- *Atta cephalotes* 25
- *Atta colombica* 25

We manually curated all predicted MRJP/yellow gene models in `webapollo v2.1.0`. Manual curation resolved several inaccuracies of the automatically generated gene models, including gene fusions, missing or shortened exons, annotation of entirely false gene models (i.e. genes not coding for MRJP/yellow-like proteins), and missing genes. It also revealed some assembly-related misannotations (frame-shift misassemblies or fragmented gene models at scaffold boundaries) in the genomes. After manual curation, the final gene sets contained between 13 (in *P. argentina*) and 24 (in *A. echinatior*) complete genes (Tab. 51).

Supplementary Table 51: Overview of manual curation of MRJP/yellow gene models in leaf-cutting ant genomes.

Species	Annotation	Number
Acromyrmex charruanus	ASSEMBLY ERROR	2
Acromyrmex charruanus	GENE	22
Acromyrmex echinatior	ASSEMBLY ERROR	2
Acromyrmex echinatior	GENE	24
Acromyrmex heyeri	FRAGMENT	2
Acromyrmex heyeri	GENE	22
Acromyrmex insinuator	ASSEMBLY ERROR	3
Acromyrmex insinuator	GENE	21
Atta cephalotes	ASSEMBLY ERROR	3
Atta cephalotes	FRAGMENT	1
Atta cephalotes	GENE	17
Atta cephalotes	PSEUDOGENE	3
Atta cephalotes	SEQUENCING ERROR	1
Atta colombica	FRAGMENT	1
Atta colombica	GENE	20
Atta colombica	PSEUDOGENE	6
Pseudoatta argentina	ASSEMBLY ERROR	4
Pseudoatta argentina	FRAGMENT	4
Pseudoatta argentina	GENE	13
Pseudoatta argentina	PSEUDOGENE	1
Pseudoatta argentina	RETROGENE	1

22 Phylogenetic reconstruction of attine major royal jelly protein genes

We inferred the phylogenetic relationship of major royal jelly (MRJP) and yellow genes in leaf-cutting ants and *Apis mellifera*. For this we first aligned protein sequences of the manually curated MRJP and yellow genes from leaf-cutting ants with `prank v.150803`. Then, we retrieved the longest annoated isoform of each MRJP/yellow gene annotated in *A. mellifera* using eutils v.5.80 and again aligned predicted protein sequences with `prank`. Both alignments were merged with prank and a phylogenetic tree was calculated with *raxML v.8.2.12* with `-f a -m PROTGAMMALG -p 12345 -x 12345 -# autoMRE`. We rerooted the tree at the split between yellow and MRJP genes in `FigTree`. Using `getCladesofSize(t1,5)`, we identified 21 different clades in the phylogeny (one clade of nine *A. mellifera* MRJP genes, nine MRJP clades containing homologs of ant MRJPs and eleven clades of *yellow*-like genes). Supplementary Figure 38 shows the phylogeny of yellow/MRJP gene families.

According to our manual annotation of this gene family, *P. argentina* lost orthologs of *MRJP01*, *MRJP07*, *MRJP08*, and *y-f*. All other *Acromyrmex* genomes contain genes coding for orthologs of these clades. The two *Atta* species both independently lost a single MRJP gene (*MRJP07* in *At. cephalotes* and *MRJP06* in *At. colombica*).

Supplementary Table 52: Overview of all identified MRJP/yellow gene families.

clade	Acep	Acha	Acol	Aech	Ahey	Ains	Amel	Parg
**A. mellifera MRJPs**	0	0	0	0	0	0	10	0
**Mrjp01**	1	2	1	1	2	1	0	0
**Mrjp02**	1	1	1	1	1	1	0	1
**Mrjp03**	1	1	1	1	1	1	0	2
**Mrjp04**	1	1	1	1	1	1	0	1
**Mrjp05**	1	1	2	4	2	2	0	1
**Mrjp06**	1	1	0	1	1	1	0	1
**Mrjp07**	0	1	1	1	1	1	0	0
**Mrjp08**	1	1	1	2	1	2	0	0
**Mrjp09**	1	4	1	3	2	3	0	1
**Y-e1**	1	1	1	1	1	1	1	1
**Y-e3**	1	1	1	1	1	1	1	1
**Y-f**	1	1	1	1	1	1	1	0
**Y-g1**	1	1	1	1	1	1	1	1
**Y-g2**	1	1	1	1	1	1	1	1
**Y-g3**	1	1	1	1	1	1	0	1
**Y-h**	1	1	1	1	1	1	1	1
**Y-like**	2	1	1	1	1	1	1	1
**Y-x1**	3	1	1	1	2	1	1	1
**Y-x2**	1	1	1	1	1	1	1	1
**Y-y**	1	1	1	1	1	1	1	1
**All Mrjp**	8	13	9	15	12	13	10	7
**All yellow**	14	11	11	11	12	11	10	10

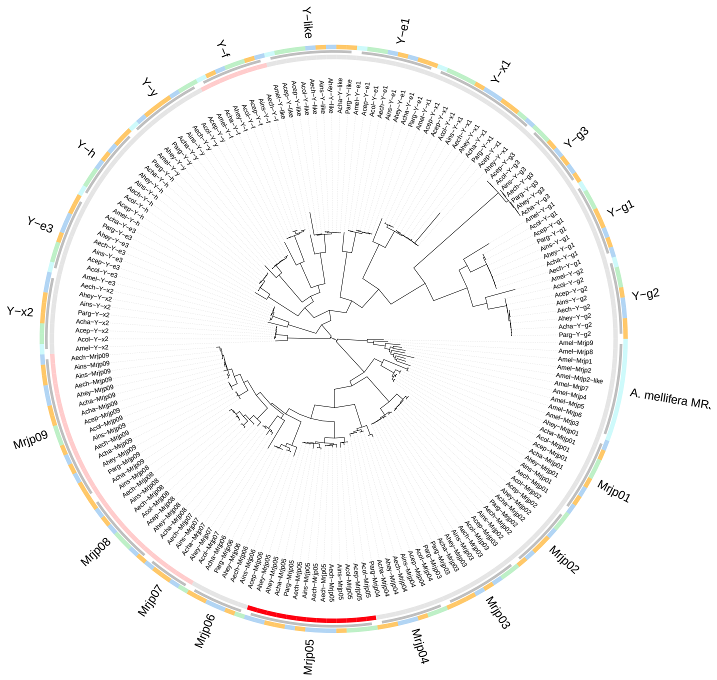

Supplementary Figure 38: MRJP/yellow gene phylogeny of leaf-cutting ant species and *Apis mellifera*.

23 Automated targeted annotation and phylogenetic tree inference of gene families

We annotated additional gene families using a fully automated approach. We used `eutils v.5.80` to retrieve all genes of a given family annotated in ants and removed any incomplete an too short or long annotations. We then used `GeMoMa v1.5.2` to produce gene models in the different attine genomes. All predicted genes were filtered for presence of conserved domains and for completeness (i.e. presence of start- and stop-codons). We annotated cuticular proteins (CPR), elongases, and gustatory receptors (GR). We used the following commands to retrieve genes for the respective families:

```
# CPRs
esearch -db protein -query "(IPR000618 OR PF00379.21 OR Chitin_bind_4 OR (Insect AND cuticle AND
↪  protein) AND txid34695[Organism:exp] AND refseq[filter]"
# Elongases
esearch -db protein -query "(IPR002076 OR PF01151 OR (ELO)) AND txid34695[Organism:exp] AND
↪  refseq[filter]"
# GRs
esearch -db protein -query "(IPR013604 OR PF08395 OR (gustatory AND receptor)) AND
↪  txid34695[Organism:exp] AND refseq[filter]"
```

We retained only those gene models coding for proteins between 100-1500 amino acids (for CPRs), 50-400 aa (for elongases), and 50-500 aa (for GRs), respectively. GeMoMa pipeline was run using the following options: `threads=10 Extractor.p=T GAF.c=F`. Domain scans were performed with `pfam_scan`, checking for presence of the domains Chitin_bind_4 in CPRs, ELO in elongases, and 7_TM7 in gustatory receptors. Gene trees were inferred for each family as follows. We first produced gene-family wide amino acid alignments with `prank v.150803` and then computed phylogenies with `FastTreeMP v2.1.10`, using default options for both programs.

Tables 53,54, and 55 provide an overview of all annotated gene models. For each gene family, we generated phylogenies (CPR: Supplementary Figure 39, elongases: Supplementary Figure 42, GRs: Supplementary Figure 45), hierachical clustering of clade sizes (CPR: Supplementary Figure 41, elongases: Supplementary Figure 44, GRs: Supplementary Figure 47), and barplots summarizing gene family sizes by species (CPR: Supplementary Figure 40, elongases: Supplementary Figure 43, GRs: Supplementary Figure 46). Gene family phylogenies were further processed in `R`, with clades again inferred using `getCladesofSize()` (with minimal clade size = 4). We included only complete gene models for GRs and elongases. The phylogeny for CPRs also contains incomplete gene models (i.e. those without an annotated start- and/or stop-codon).

Supplementary Table 53: Summary of cuticular proteins annotated in leaf-cutting ants.

Type	Acep	Acha	Acol	Aech	Ahey	Ains	Parg
**complete**	32	33	34	32	31	31	31
**CTE**	0	1	0	1	0	0	0
**NTE**	7	7	8	7	8	8	10
**NC**	0	1	0	0	1	0	0

Supplementary Table 54: Summary of elongases annotated in leaf-cutting ants.

Type	Acep	Acha	Acol	Aech	Ahey	Ains	Parg
**complete**	13	12	14	14	11	12	11
**CTE**	0	0	0	0	2	0	0
**NTE**	1	1	0	2	2	3	3
**NC**	0	0	0	0	0	0	0

Supplementary Table 55: Summary of gustatory receptors annotated in leaf-cutting ants.

Type	Acep	Acha	Acol	Aech	Ahey	Ains	Parg
**complete**	62	60	62	62	61	61	27
**CTE**	0	3	3	3	2	2	2
**NTE**	5	6	6	4	6	3	2
**NC**	0	0	0	0	0	0	0

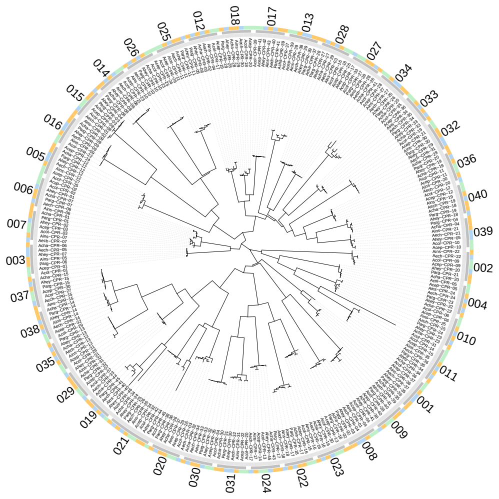

Supplementary Figure 39: Cuticular protein gene phylogeny of leaf-cutting ant species.

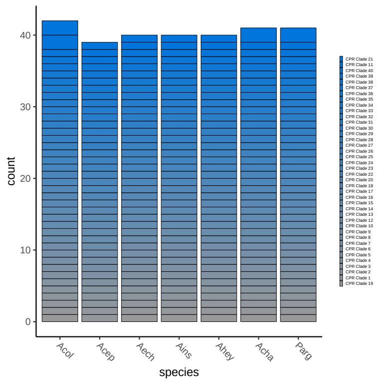

Supplementary Figure 40: Barplot summarizing CPR gene family sizes in different leaf-cutting ant species.

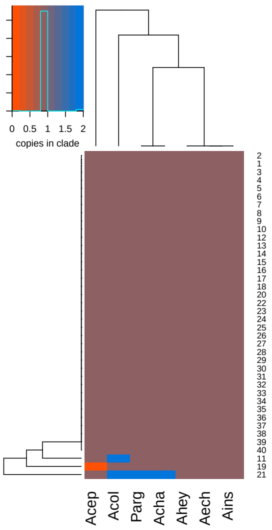

Supplementary Figure 41: Hierachical clustering of clade sizes for the CPR gene family in leaf-cutting ants.

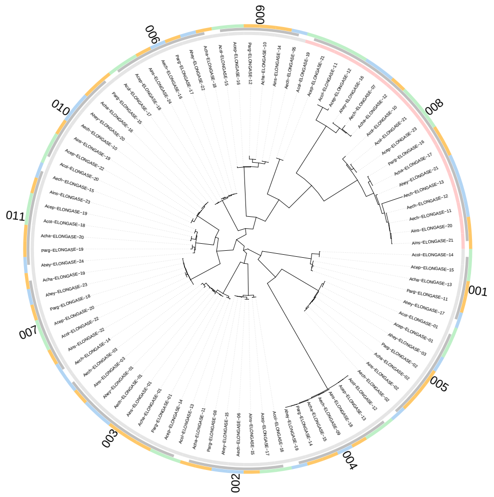

Supplementary Figure 42: Elongase gene phylogeny of leaf-cutting ant species.

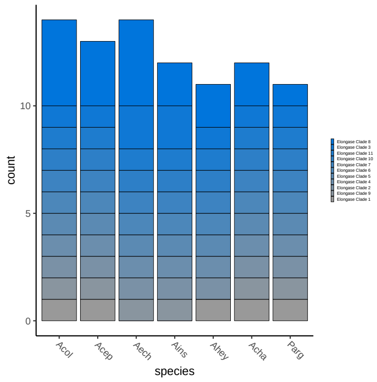

Supplementary Figure 43: Barplot summarizing elongase gene family sizes in different leaf-cutting ant species.

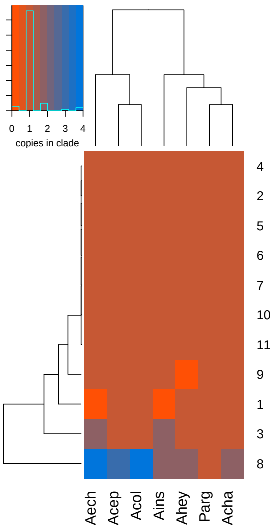

Supplementary Figure 44: Hierachical clustering of clade sizes for the elongase gene family in leaf-cutting ants.

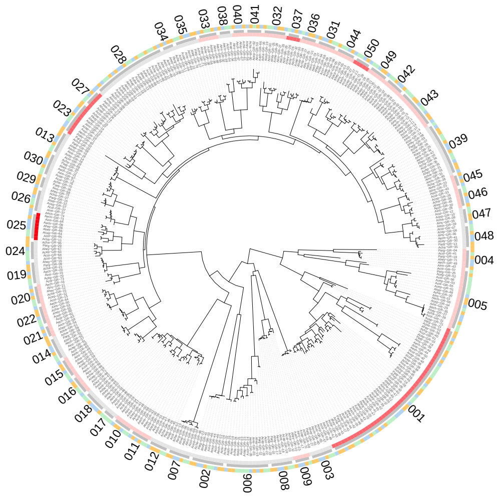

Supplementary Figure 45: Gustatory receptor gene phylogeny of leaf-cutting ant species.

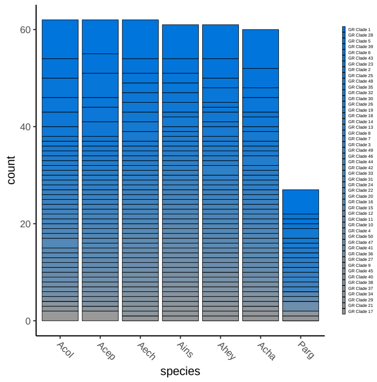

Supplementary Figure 46: Barplot summarizing gustatory receptor gene family sizes in different leaf-cutting ant species.

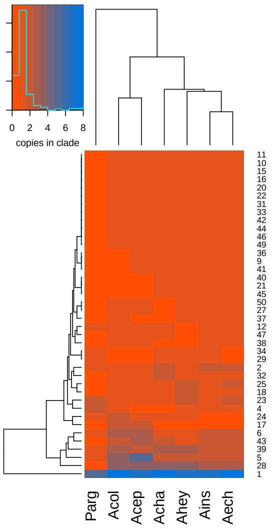

Supplementary Figure 47: Hierachical clustering of clade sizes for the gustatory receptor gene family in leaf-cutting ants.

24 Whole genome alignments

We generated whole genome alignments for all attine species with the phylogeny-aware aligner `progressiveCactus v0.1` (default settings).

```
(((TCOR:0.158672,(TSEP:0.137711,(((AHEY:0.024983,(ACHA:0.016335,PARG:0.016335):0.008648):0.027567,
↪  (AECH:0.009605,AINS:0.009605):0.042945):0.065115,(ACEP:0.020492,ACOL:0.020492):0.097173):0.020045)
↪  :0.020962):0.067305,MZET:0.225977):0.094428,CCOS:0.320405);
ACHA Acromyrmex_charruanus.v2.1.fa
AHEY Acromyrmex_heyeri.v2.1.fa
AINS Acromyrmex_insinuator.v2.1.fa
PARG Pseudoatta_argentina.v2.1.fa
*AECH Acromyrmex_echinatior.v2.0.fa
ACEP Atta_cephalotes.v2.0.fa
ACOL Atta_colombica.v2.0.fa
CCOS Cyphomyrmex_costatus.v2.0.fa
TCOR Trachymyrmex_cornetzi.v2.0.fa
TSEP Trachymyrmex_septentrionalis.v2.0.fa
TZET Trachymyrmex_zeteki.v2.0.fa
```

We then used `halSummarizeMutations` to summarize inferred mutations at each branch of the underlying attine phylogeny. We calculated transposition (P), insertion (I), deletion (D), inversion (V), and duplication (U) events per million years (Ma) of evolution, based on inferred divergence estimates from the phylogeny. We subsequently grouped mutation events by size (i.e. number of affected bases) for each branch in the phylogeny. Finally, we calculated for each mutation type the relative number of events per Ma as the percent increase or decrease compared to the mean number of mutational events across all branches. Supplementary Figure 48 shows details for Inversions, Transpositions, and Deletions that are most abundant in social parasite lineages. Supplementary Figures 49, 50, and 51 provide information about the size (in bp) of the different mutation types.

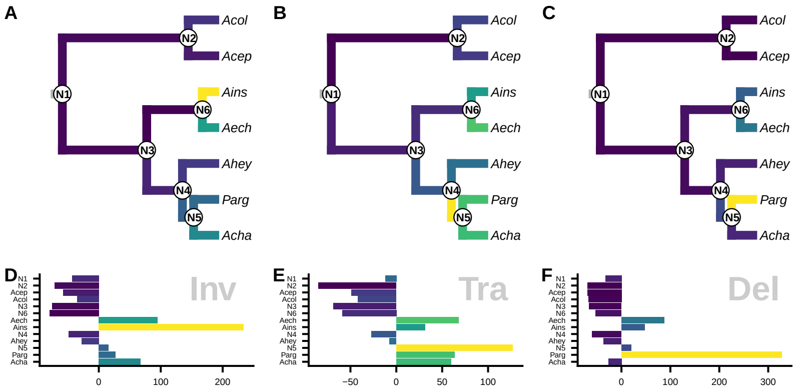

Supplementary Figure 48: Relative increase/decrease (in percent) of Inversions (A,D), Transpositions (B,E), and Deletions (C,F) across the leaf-cutting ant phylogeny. Branches colored based on relative changes from low (purple) to high (yellow).

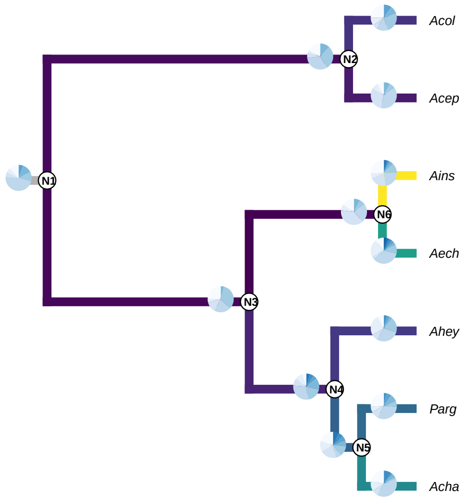

Supplementary Figure 49: Relative increase/decrease of inversions inferred from whole-genome alignment of attine genomes. The pie charts summarize the length (in bp) affected by each mutation event from short (light blue) to long (dark blue)

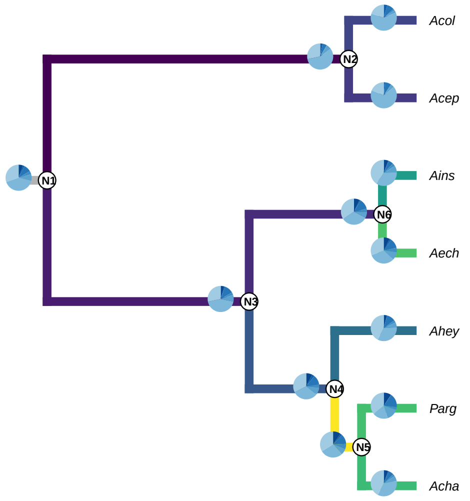

Supplementary Figure 50: Relative increase/decrease of transpositions inferred from whole-genome alignment of attine genomes. The pie charts summarize the length (in bp) affected by each mutation event from short (light blue) to long (dark blue)

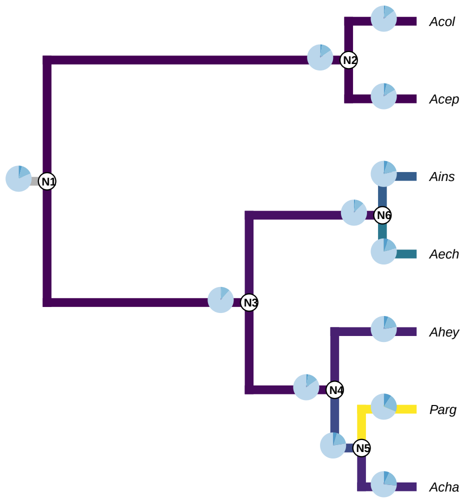

Supplementary Figure 51: Relative increase/decrease of deletions inferred from whole-genome alignment of attine genomes. The pie charts summarize the length (in bp) affected by each mutation event from short (light blue) to long (dark blue)

Supplementary Table 56: Overview of the number of genes affected by genome rearrangements, relative to either the species' own gene coordinates (columns ”own coordinates”) or coordinates liftover from the sister species (columns ”liftoveer coordinates”).

	own coordinates			liftover coordinates		
	**Deletions**	**Inversions**	**Transpositions**	**Deletions**	**Inversions**	**Transpositions**
Ains	2	7	75	717	12	99
Aech	2	1	122	874	2	121
Ahey	0	0	6	685	5	131
Parg	6	6	155	1509	0	9
Acha	1	5	131	553	0	6
N5	22*	2	169	-	0	7

*: gene coordinates at N4 inferred from annotations in *A. heyeri*.

25 Gene level effects of genomic rearrangements

To analyse to what extent genes are affected by genomic rearrangements, we overlapped (using `bedtools intersect` (v2.20.1)) protein-coding gene annotations with inferred coordinates of inversions, deletions and transpositions from our whole genome alignment. For estimating overlap of deletions we inferred gene coordinates in the ancestral reconstructed genome, i.e. liftover (with `haltools` v2.1) of gene annotations from e.g. *P. argentina* to the reconstructed genome at N5:

```
halLiftover attines.hal PARG Parg.genes.bed N5 N5.Parg.genes.bed
```

Similarly, estimates for inversions, and transpositions at N5 are based on gene coordinates inferred from *A. charruanus*. For deletions at N5, we inferred gene coordinates at the Acha/Parg/Ahey stem group (N4), using a liftover from *A. heyeri* gene annotations. Genes overlapping identified repeats were removed. Given the low syntenic conversion of the ant genomes and the consequential fragmentation of the whole genome alignment, we emphasize that this analysis is only exploratory and its results expected to have a high degree of uncertainty.

Relative to a species' own gene coordinates, we find between 0 (in Ahey) to 7 (in Ains) genes affected by inversions, between 6 (Ahey) to 169 genes (N5) affected by transpositions, and 0 (Ahey) to 22 genes (N5) affected by deletions. When using gene coordinates created by liftover from a sister species, we find substantially more genes affected by deletions (between 553 in Acha to 1509 in Parg). The numbers are also very different for transpositions (6 to 131 genes affected) and inversions (0 to 12 genes affected). See Table 56 for details.

The estimated gene-level effects of genomic rearrangements are largely congruent with the differences in the relative frequency of inversions, transpositions and deletions in the socially parasitic lineages. Inversion, which were most frequent during the evolution of *A. insinuator* also had the largest effect on genes in this species. Similarly, Transpositions were affecting most genes in N5, the presumably socially parasitic stem group of *A. charruanus* and *P. argentina*. Finally, deletions showed the strongest effect at the gene level in *P. argentina*.

26 Synteny analysis

We inferred synteny between ortholog genes across *At. colombica* and the five *Acromyrmex* species using `i-ADHoRe v3.0`. First, we inferred orthology between protein coding genes using `orthofinder v2.2.6`. Based on the inferrred orthogroups, we ran `i-ADHoRe` using settings that allowed us to detect regions of imperfect synteny:

```
blast_table=data/Orthologues.list
table_type=family
prob_cutoff=0.001
anchor_points=3
number_of_threads=16
visualizeAlignment=true
write_stats=true
output_path= output2
alignment_method=gg2
gap_size=50
cluster_gap=55
level_2_only=true
q_value=.05
```

We subsequently processed the inferred pairwise multiplicons in `R`, restricting our analyses to longer regions spanning at least 25 genes in both genomes.

This filtering reduced the number of analysed syntencic regions from 5,425 to 1,803. We calculated Kendall's rank correlation coefficients (τ) for each syntenic region. A perfectly syntenic region has a correlation coefficient of 1. Similarly, regions only differing by gene gains/losses but otherwise conserved synteny will have a τ =1 as well, due to Kendall's correlation being rank-based (see Supplementary Figure 52). Supplementary Figure 53 shows different syntenic regions and their difference in τ. We here use τ as a quanititative measure of syntenic conservation across all inferred multiplicons. To analyse whether syntenic changes are more frequent in the inquiline species than in the hosts, we compared τ between the *At. colombica* and the different *Acromyrmex* species for 128 syntenic regions between ACOL and AHEY, 125 between ACOL and AECH, 121 between ACOL and PARG, 125 between ACOL and ACHA, and 111 between ACOL and AINS. We tested for a significant decrease in syntenic conservation relative to ACOL comparing the two host genomes (AECH and AHEY) to the three inquline social parasite species (AINS, ACHA, and PARG) using a one-sided approximate (Monte Carlo) Wilcoxon-Mann-Whitney Test (`wilcox_test()` from the `R` package `coin`). Our analysis showed that synteny in inquiline genomes is significantly reduced compared to the two non-parasitic *Acromyrmex* species (Fig. 54).

```
wilcox_test(c(hosts,parasites)~f,distribution = approximate(nresample = 1000000),alternative="greater")
```

```
Approximative Wilcoxon-Mann-Whitney Test
data:  c(hosts, parasites) by f (hosts_f, parasite_f)
Z = 2.6715, p-value = 0.003796
alternative hypothesis: true mu is greater than 0
```

Comparing host-parasite pairs individually, the results are as follows:

```
AECH vs AINS: test statistic = 2.0573, p-value = 0.01914
AHEY vs ACHA: test statistic = 0.4632, p-value = 0.3223
AHEY vs PARG: test statistic = 2.3559, p-value = 0.00907
```

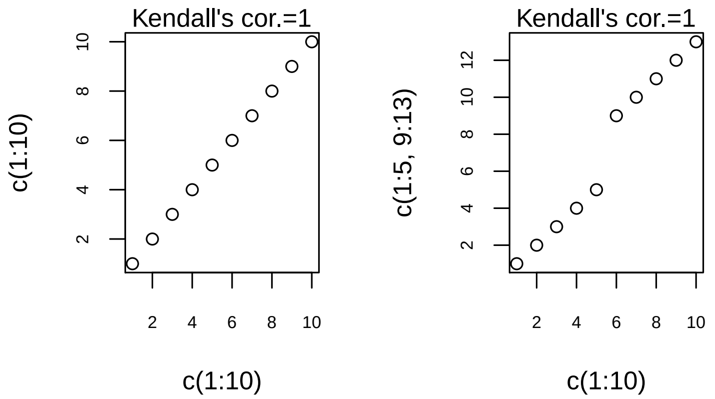

Supplementary Figure 52: Illustration of Kendalls'r rank correlation (τ) for a syntenic region containing gene losses/gains and a perfectly syntenic region.

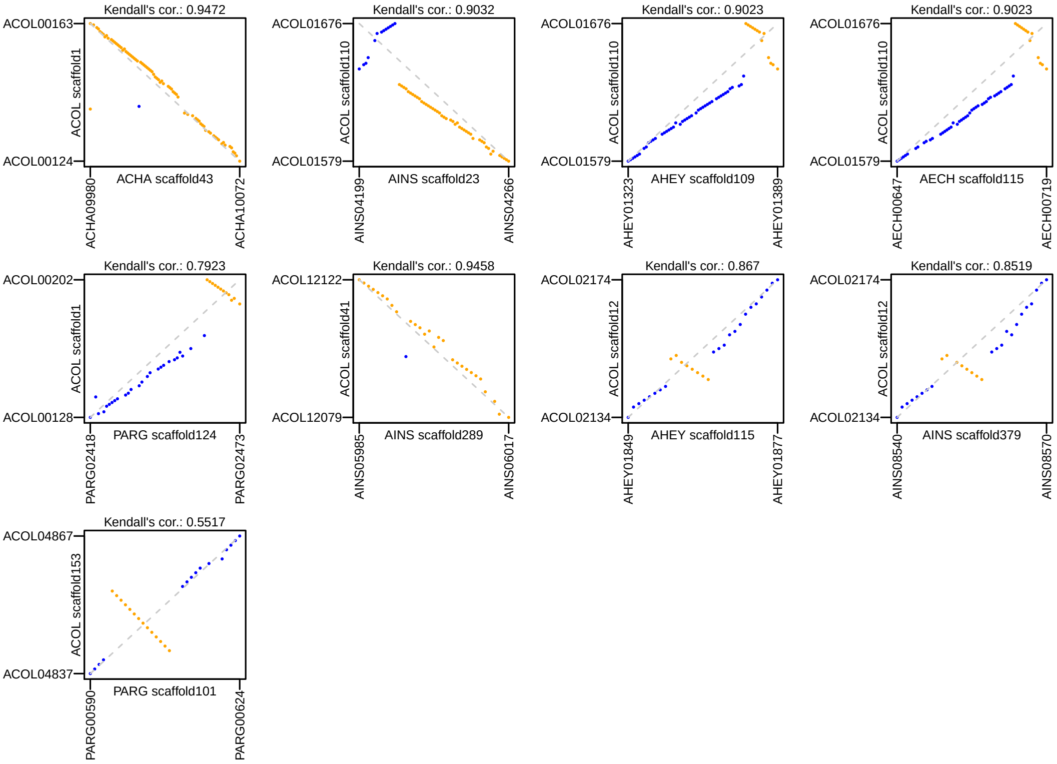

Supplementary Figure 53: Dot plots showing examples of syntenic regions between different species and the inferred Kendalls'r rank correlation (τ) as a measure of syntenic conservation. Blue dots show genes with same strandedness between both genomes, orange dots show genes with inverse strandedness.

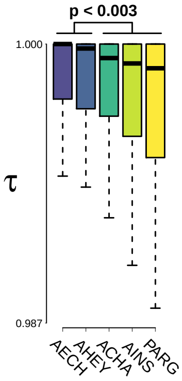

Supplementary Figure 54: Comparison of τ as a measure of syntenic conservation between the genome of *At. colombica* and host (AECH, AHEY) and inquiline parasite genomes (ACHA, AINS, PARG). Statistically significance was assessed using one-sided Mann-Whitney tests.

27 Supplementary References

1. Nygaard, S., Hu, H., Li, C., Schiøtt, M., Chen, Z., Yang, Z., et al. Reciprocal genomic evolution in the ant–fungus agricultural symbiosis. *Nature Communications*, 7(1), 12233. http://doi.org/10.1038/ncomms12233 (2016)
2. Li, H., Sosa-Calvo, J., Horn, H. A., Pupo, M. T., Clardy, J., Rabeling, C., et al. Convergent evolution of complex structures for ant-bacterial defensive symbiosis in fungus-farming ants. *Proceedings of the National Academy of Sciences of the United States of America*, 62(42), 201809332–6. http://doi.org/10.1073/pnas.1809332115 (2018)
3. Nygaard, S., Zhang, G., Schiøtt, M., Li, C., Wurm, Y., Hu, H., et al. The genome of the leaf-cutting ant *Acromyrmex echinatior* suggests key adaptations to advanced social life and fungus farming. *Genome Research*, 21(8), 1339–1348. http://doi.org/10.1101/gr.121392.111 (2011)
4. Mikheyev, A. S., Mueller, U. G., Abbot, P. Comparative Dating of Attine Ant and Lepiotaceous Cultivar Phylogenies Reveals Coevolutionary Synchrony and Discord. *The American Naturalist*, 175(6), E126–E133. http://doi.org/10.1086/652472 (2010)
5. Schultz, T. R., Brady, S. G. Major evolutionary transitions in ant agriculture. *Proceedings of the National Academy of Sciences*, 105(14), 5435–5440. http://doi.org/10.1073/pnas.0711024105 (2008)
6. Ješovnik, A., González, V. L., Schultz, T. R. Phylogenomics and Divergence Dating of Fungus-Farming Ants (Hymenoptera: Formicidae) of the Genera *Sericomyrmex* and *Apterostigma*. *PLoS ONE*, 11(7), e0151059–18. http://doi.org/10.1371/journal.pone.0151059 (2016)
7. Branstetter, M. G., Ješovnik, A., Sosa-Calvo, J., Lloyd, M. W., Faircloth, B. C., Brady, S. G., Schultz, T. R. Dry habitats were crucibles of domestication in the evolution of agriculture in ants. *Proceedings of the Royal Society B: Biological Sciences*, 284(1852), 20170095–10. http://doi.org/10.1098/rspb.2017.0095 (2017)
8. Schiffels S, Wang K. MSMC and MSMC2: The Multiple Sequentially Markovian Coalescent. Methods Mol Biol. 2090:147-166. http://doi.org/10.1007/978-1-0716-0199-0$_{7}(2020)$
9. Liu, H., Jia, Y., Sun, X., Tian, D., Hurst, L. D., Yang, S. Direct Determination of the Mutation Rate in the Bumblebee Reveals Evidence for Weak Recombination-Associated Mutation and an Approximate Rate Constancy in Insects. *Molecular Biology and Evolution*, 34(1), 119–130. http://doi.org/10.1093/molbev/msw226 (2017)
10. Wright, F. The 'effective number of codons' used in a gene. Gene 87(1): 23-29. http://doi.org/10.1016/0378-1119(90)90491-9 (1990)
11. Sueoka, N. Translation-coupled violation of Parity Rule 2 in human genes is not the cause of heterogeneity of the DNA G+ C content of third codon position. Gene, 238(1), 53-58. http://doi.org/10.1016/s0378-1119(99)00320-0 (1999)
12. Rubin, B. E. and C. S. Moreau. Comparative genomics reveals convergent rates of evolution in ant-plant mutualisms. Nat Commun 7: 12679. http://doi.org/10.1038/ncomms12679 (2016)
13. McKenzie, S. K., Fetter-Pruneda, I., Ruta, V., Kronauer, D. J. C. Transcriptomics and neuroanatomy of the clonal raider ant implicate an expanded clade of odorant receptors in chemical communication. Proceedings of the National Academy of Sciences of the United States of America, 113(49), 14091–14096. http://doi.org/10.1073/pnas.1610800113 (2016)
14. Zhou, X., Slone, J. D., Rokas, A., Berger, S. L., Liebig, J., Ray, A., et al. Phylogenetic and Transcriptomic Analysis of Chemosensory Receptors in a Pair of Divergent Ant Species Reveals Sex-Specific Signatures of Odor Coding. *PLOS Genetics*, 8(8), e1002930–18. http://doi.org/10.1371/journal.pgen.1002930 (2012)
15. McKenzie, S. K., Kronauer, D. J. C. The genomic architecture and molecular evolution of ant odorant receptors. *Genome Research*, 28(11), 1757–1765. http://doi.org/10.1101/gr.237123.118 (2018)
