# Supplementary material for: Relaxed selection underlies genome erosion in socially parasitic ant species
Source: Nat Commun. 2021 May 18;12:2918. doi: 10.1038/s41467-021-23178-w (PMC8131649; doi:10.1038/s41467-021-23178-w)
Supplement: Supplementary file 3 — Reporting Summary [file 41467_2021_23178_MOESM3_ESM.pdf]

nature research

Corresponding author(s): Lukas Schrader, Jacobus J. Boomsma, Christian Rabeling

Last updated by author(s): Mar 30, 2021

Reporting Summary

Nature Research wishes to improve the reproducibility of the work that we publish. This form provides structure for consistency and transparency in reporting. For further information on Nature Research policies, see our Editorial Policies and the Editorial Policy Checklist.

Statistics

For all statistical analyses, confirm that the following items are present in the figure legend, table legend, main text, or Methods section.

n/a	Confirmed	
☐	☒	The exact sample size (*n*) for each experimental group/condition, given as a discrete number and unit of measurement
☐	☒	A statement on whether measurements were taken from distinct samples or whether the same sample was measured repeatedly
☐	☒	The statistical test(s) used AND whether they are one- or two-sided<br>*Only common tests should be described solely by name; describe more complex techniques in the Methods section.*
☒	☐	A description of all covariates tested
☐	☒	A description of any assumptions or corrections, such as tests of normality and adjustment for multiple comparisons
☐	☒	A full description of the statistical parameters including central tendency (e.g. means) or other basic estimates (e.g. regression coefficient) AND variation (e.g. standard deviation) or associated estimates of uncertainty (e.g. confidence intervals)
☐	☒	For null hypothesis testing, the test statistic (e.g. F, t, r) with confidence intervals, effect sizes, degrees of freedom and P value noted<br>*Give P values as exact values whenever suitable.*
☐	☒	For Bayesian analysis, information on the choice of priors and Markov chain Monte Carlo settings
☐	☒	For hierarchical and complex designs, identification of the appropriate level for tests and full reporting of outcomes
☒	☐	Estimates of effect sizes (e.g. Cohen's *d*, Pearson's *r*), indicating how they were calculated

Our web collection on statistics for biologists contains articles on many of the points above.

Software and code

Policy information about availability of computer code

Data collection	SOAPdenovo Init V1.06, V2.04, GapCloser v1.12, TRF v4.07b, Wu-BlastX 2.0, RepeatMasker v4.0.6, LTR-FINDER v1.06, PALS v1.0, glean v1.0.1, genewise v2.0, augustus v2.5.5, Tophat v2.1.0, Cufflinks v2.0.2, quast v5.0.2, BUSCO v3, GAG v2.0.1, bwa v0.7.17-r1188, trimmomatic 0.39, exonerate v2.2.0, GeMoMa v1.5.2, EvidenceModeler v1.1.1, mmseqs2 v. de06950ff733478fc87195f9c57683c7dd7207e8, bedtools v2.20.1, additional information is available at https://github.com/schraderL/inquilineGenomics
Data analysis	orthofinder v.2.2.6, prank v.150803, pal2nal v.14, jModelTest v.2.1.10, PAUP* v.4a163, MrBayes v3.2.6, RaxML v8.2.12, PAML v4.9h, MSMC2 v2.1.1, HYPHY v2.3.14, MCL v.14-137, blastp v.2.6.0+, TransposonPSI v.08222010, CAFE v4.0, FigTree v1.4.3, mafft v7.307, FastTreeMP v2.1.10, dlcpar 2.0.1, GenomeTools v1.5.9, prank v.150803, gotree-reroot v0.4.0, dlcoal v1.0, eutils v.5.80, signalP v4.1, webapollo v2.1.0, progressiveCactus v0.1, , haltools v2.1, i-ADHoRe v3.0, additional information is available at https://github.com/schraderL/inquilineGenomics

For manuscripts utilizing custom algorithms or software that are central to the research but not yet described in published literature, software must be made available to editors and reviewers. We strongly encourage code deposition in a community repository (e.g. GitHub). See the Nature Research guidelines for submitting code & software for further information.

Data

Policy information about availability of data

All manuscripts must include a data availability statement. This statement should provide the following information, where applicable:

- Accession codes, unique identifiers, or web links for publicly available datasets
- A list of figures that have associated raw data
- A description of any restrictions on data availability

Raw sequencing data, genome assemblies and annotations have been deposited in GenBank with BioProject accession codes PRJNA552756, PRJNA605929, and

PRJNA714946 and in the CNGB Sequence Archive with accession codes CNP0000530 and CNP0000905. Further data is available in figshare with identifier doi:10.6084/m9.figshare.14224253. Previously published ant genomes were retrieved from GenBank.

Field-specific reporting

Please select the one below that is the best fit for your research. If you are not sure, read the appropriate sections before making your selection.

☐ Life sciences ☐ Behavioural & social sciences ☒ Ecological, evolutionary & environmental sciences

For a reference copy of the document with all sections, see nature.com/documents/nr-reporting-summary-flat.pdf

Ecological, evolutionary & environmental sciences study design

All studies must disclose on these points even when the disclosure is negative.

Study description	Comparative genomic analysis of inquiline socially parasitic ants and related free-living species.
Research sample	Individuals of three inquiline socially parasitic ant species (Acromyrmex charruanus from Uruguay, Acromyrmex insinuator from Panama, Pseudoatta argentina from Uruguay) and their two host species (Acromyrmex echinatior from Panama, Acromyrmex heyeri from Uruguay) were collected for genome and transcriptome sequencing. For each species, only individuals from a single colony were used. For DNA extractions, we used mature workers (i.e. non-reproductive females) for host species, to ensure obtaining sufficient DNA. For social parasite species, we extracted DNA from gynes (i.e. young reproductive females), as only few or no workers are produce by these species. For RNA extractions, we pooled available castes (workers and gynes), sexes (males and females), and developmental stages (eggs, larvae and pupae) for the different species to get a representative sample of gene expression for subsequent gene annotations.
Sampling strategy	Individuals were collected from field collected colonies and divided by species (host and parasite). For genome and transcriptome sequencing, we selected a single representative colony for each species in our comparison, following common practices in comparative genomics.
Data collection	Samples were collected by Martin Bollazzi, Christian Rabeling in Uruguay and by Jacobus Boomsma, and Morten Schiøtt in Panama by excavating colonies of the respective host species using standard digging equipment.
Timing and spatial scale	Samples were collected during the southern hemisphere fall in 2013 for the A. charruanus, P. argetina and A. heyeri and in 2014 for A. insinuator. Sampling times were determined by mating flight season of the different species, to increase likelihood of finding a sufficiently large number of gynes and males in the colonies.
Data exclusions	No data was excluded for the study.
Reproducibility	Reproducability of all analyses was ensured by documenting and reporting bioinformatic pipelines underlying the study. All required code and data to replicate our findings are made available online, in the Supplementary Material and the github code repository accompanying the paper.
Randomization	Individuals selected for sequencing were selected randomly.
Blinding	All analyses were performed blind in respect to the outcome.

Did the study involve field work? ☒ Yes ☐ No

Field work, collection and transport

Field conditions	Not relevant for the current study because we focus on genetic data.
Location	Colonies of Acromyrmex heyeri and its social parasites Acromyrmex charruanus and Pseudoatta argentina were collected near Cerro Colorado, Department of Florida, in Uruguay (-33.9042, -55.5941). Acromyrmex insinuator was collected from colonies of its host Acromyrmex echinatior in Gamboa, Panama (9.1203, -79.6971).
Access & import/export	Ant nests were carefully excavated and transferred to a sorting table. Ants were sorted by species before sampling in RNAlater or liquid nitrogen. The Visitor's Office of the Smithsonian Tropical Research Institute provided logistic help and facilities to work in Gamboa, Panama. The Direccion General de Recursos Naturales Renovables and the Autoridad Nacional del Ambiente y el Mar (ANAM) gave permission to sample and export ants from Uruguay and Panama, respectively. Permits by the Direccion General de Recursos Naturales Renovables in Uruguay were issued in 2012 for the years 2012-2014 to Martin Bollazzi and Christian Rabeling. Permits by ANAM for the Panamanian species were issued in 2014 to the research program of Jacobus J. Boomsma.
Disturbance	Excavation sites were carefully closed after extraction of the colonies.

Reporting for specific materials, systems and methods

We require information from authors about some types of materials, experimental systems and methods used in many studies. Here, indicate whether each material, system or method listed is relevant to your study. If you are not sure if a list item applies to your research, read the appropriate section before selecting a response.

Materials & experimental systems

n/a	Involved in the study
☒	☐ Antibodies
☒	☐ Eukaryotic cell lines
☒	☐ Palaeontology and archaeology
☐	☒ Animals and other organisms
☒	☐ Human research participants
☒	☐ Clinical data
☒	☐ Dual use research of concern

Methods

n/a	Involved in the study
☒	☐ ChIP-seq
☒	☐ Flow cytometry
☒	☐ MRI-based neuroimaging

Animals and other organisms

Policy information about studies involving animals; ARRIVE guidelines recommended for reporting animal research

Laboratory animals	The study did not involve laboratory animals.
Wild animals	Mature colonies (presumably >3 years old) of the two Acromyrmex species A. heyeri and A. echinatior were identified at the respective field site in Uruguay and Panama. Subterranean nest cavities containing the fungus garden were carefully opened and the fungus garden and the majority of all ants including males, queens, gynes, workers and brood were collected into fluon coated buckets. After transferring colonies to field station laboratories, host and parasite individuals were separated and subbsequently killed by sampling in RNAlater or liquid nitrogen for subsequent DNA and RNA extraction for genome and transcriptome sequencing.
Field-collected samples	Any animals collected for the study were killed shortly after sampling for subsequent DNA/RNA extraction.
Ethics oversight	This study followed the ASAB/ABS Guidelines for the Use of Animals in Research. Animals were kept in the best possible conditions based on the biology of the species. We also adhered to the legal requirements of the University of Rochester and Arizona State University in the United States of America, the University of Copenhagen in Denmark, and legal requirements of Panama and Uruguay.

Note that full information on the approval of the study protocol must also be provided in the manuscript.
